# SIPA1L2 controls trafficking and local signaling of TrkB-containing amphisomes at presynaptic terminals

**DOI:** 10.1038/s41467-019-13224-z

**Published:** 2019-11-29

**Authors:** Maria Andres-Alonso, Mohamed Raafet Ammar, Ioana Butnaru, Guilherme M. Gomes, Gustavo Acuña Sanhueza, Rajeev Raman, PingAn Yuanxiang, Maximilian Borgmeyer, Jeffrey Lopez-Rojas, Syed Ahsan Raza, Nicola Brice, Torben J. Hausrat, Tamar Macharadze, Silvia Diaz-Gonzalez, Mark Carlton, Antonio Virgilio Failla, Oliver Stork, Michaela Schweizer, Eckart D. Gundelfinger, Matthias Kneussel, Christina Spilker, Anna Karpova, Michael R. Kreutz

**Affiliations:** 1 Leibniz Group ‘Dendritic Organelles and Synaptic Function’, Center for Molecular Neurobiology, ZMNH, University Medical Center Hamburg-Eppendorf, 20251 Hamburg, Germany; 2 RG Neuroplasticity, Leibniz Institute for Neurobiology, 39118 Magdeburg, Germany; 3 Department of Genetics & Molecular Neurobiology, Institute of Biology, Otto-von-Guericke University, 39120 Magdeburg, Germany; 4 Paradigm Therapeutics Ltd. Cambridge, UK Cerevance, Cambridge, UK; 5 Institute of Molecular Neurogenetics, Center for Molecular Neurobiology, ZMNH, University Medical Center Hamburg-Eppendorf, 20251 Hamburg, Germany; 6 UKE Microscopy Imaging Facility, University Medical Center Hamburg-Eppendorf, 20246 Hamburg, Germany; 7 Center for Behavioral Brain Sciences, Otto von Guericke University, 39120 Magdeburg, Germany; 8 ZMNH Core Facility Morphology and Electron Microscopy, Center for Molecular Neurobiology, ZMNH, University Medical Center Hamburg-Eppendorf, 20251 Hamburg, Germany; 9 Department of Neurochemistry and Molecular Biology, Leibniz Institute for Neurobiology, 39118 Magdeburg, Germany; 10 Medical Faculty, Otto von Guericke University, 39120 Magdeburg, Germany

**Keywords:** Organelles, Neuroscience, Cellular neuroscience, Synaptic plasticity, Synaptic transmission

## Abstract

Amphisomes are organelles of the autophagy pathway that result from the fusion of autophagosomes with late endosomes. While biogenesis of autophagosomes and late endosomes occurs continuously at axon terminals, non-degradative roles of autophagy at boutons are barely described. Here, we show that in neurons BDNF/TrkB traffick in amphisomes that signal locally at presynaptic boutons during retrograde transport to the soma. This is orchestrated by the Rap GTPase-activating (RapGAP) protein SIPA1L2, which connects TrkB amphisomes to a dynein motor. The autophagosomal protein LC3 regulates RapGAP activity of SIPA1L2 and controls retrograde trafficking and local signaling of TrkB. Following induction of presynaptic plasticity, amphisomes dissociate from dynein at boutons enabling local signaling and promoting transmitter release. Accordingly, *sipa1l2* knockout mice show impaired BDNF-dependent presynaptic plasticity. Taken together, the data suggest that in hippocampal neurons, TrkB-signaling endosomes are in fact amphisomes that during retrograde transport have local signaling capacity in the context of presynaptic plasticity.

## Abstract (web summary)

There is growing evidence that autophagy might serve specialized functions in neurons besides its role in protein homeostasis. In this study, authors demonstrate that axonal retrograde transport of BDNF/TrkB in neuronal amphisomes is involved in plasticity-relevant local signaling at presynaptic boutons and that SIPA1L2, a member of the SIPA1L family of neuronal RapGAPs, associates via LC3b to TrkB-containing amphisomes to regulate its motility and signaling at the axon terminals

## Introduction

BDNF/TrkB signaling is required for a variety of neuronal functions including neurotransmission and synaptic plasticity. Upon binding to BDNF, BDNF/TrkB is internalized via pinocytosis^1,2^, or in a clathrin-dependent manner^3^ to vesicular compartments called signaling endosomes that might allow for local signaling. Signaling endosomes are predominantly generated at axon terminals by different mechanisms and constitute long-lived organelles that persist while undergoing transport from nerve terminals to the neuronal soma^4–6^. In neurons, retrograde trafficking of TrkB signaling endosomes constitutes an important long-range signaling mechanism that conveys information on presynaptic activity to the cell soma^7^. How endosomal sorting is accomplished in these organelles and how they escape from the degradative pathway remains still unclear.

A key consequence of TrkB signaling is sustained ERK activation, a process achieved via activation of the small GTPase Rap1 (Supplementary Fig. 1a)^8^. Accordingly, prenylated Rap1 is associated with TrkB signaling endosomes and regulation of its GTPase activity is, therefore, a likely mechanism to control local TrkB signaling.

A regulator of Rap1 is SIPA1L2 (also known as SPAR2), a member of the SIPA1L family of neuronal RapGAPs (Supplementary Fig. 1b–d)^9^. The protein is most abundant in granule cells of the dentate gyrus (DG) and cerebellum and shows RapGAP activity for Rap1 and 2^10^. This RapGAP activity promotes the intrinsic GTPase activity of Rap1/2 that catalyzes the hydrolysis of GTP to GDP and inactivates Rap1/2 and consequently, ERK signaling. Here, we report that *sipa1l2* knockout (ko) mice show impaired long-term potentiation (LTP) at mossy fiber (MF) synapses and spatial pattern separation, which requires MF plasticity. MF-LTP is an NMDA receptor-independent form of LTP that is expressed presynaptically and depends upon local BDNF/TrkB signaling^11^. Accordingly, we found that SIPA1L2 directly binds to TrkB and application of a TAT-peptide encompassing the binding region for TrkB in SIPA1L2 induces a similar phenotype in vivo and in vitro in wild-type (wt) mice like those observed in *sipa1l2* ko mice. We found that SIPA1L2 links the receptor tyrosine kinase to a dynein motor via a direct interaction with the adaptor protein Snapin which allows retrograde transport. Interestingly, SIPA1L2 concurrently interacts with Light chain 3 (LC3), a marker for autophagosomes that is involved in substrate selection, and this interaction promotes SIPA1L2 RapGAP activity. While autophagosomes are continuously generated at axon terminals, very little is known about the synaptic role of autophagy. Here we show that SIPA1L2 associates with amphisomes, organelles from the autophagic pathway that result from the fusion of autophagosomes with late endosomes, that are positive for the late endosome marker Rab7 as well as LC3 and TrkB. This configuration allows LC3 to tightly control TrkB signaling via interaction with SIPA1L2, which increases the RapGAP activity and promotes Rap1/ERK inactivation. We show that these amphisomes traffick retrogradely along axons, stop at presynaptic boutons and both motility and signaling are controlled by SIPA1L2’s RapGAP activity that reduces the velocity of amphisome transport. Presynaptic LTP induces a protein kinase A (PKA)-dependent dissociation of the SIPA1L2/Snapin complex from dynein intermediate chain (DIC). This increases dwelling time of the amphisome at presynaptic boutons and PKA phosphorylation of SIPA1L2 reduces RapGAP activity, therefore, enabling local TrkB signaling at boutons, which in turn promotes neurotransmitter release. Collectively, the data suggest that retrograde axonal transport of BDNF/TrkB occurs in neuronal amphisomes that allow local control of TrkB signaling and are involved in plasticity-relevant local signaling at presynaptic boutons.

## Results

### MF-LTP and pattern separation deficits in sipa1l2^−/−^ mice

**Fig. 1:**
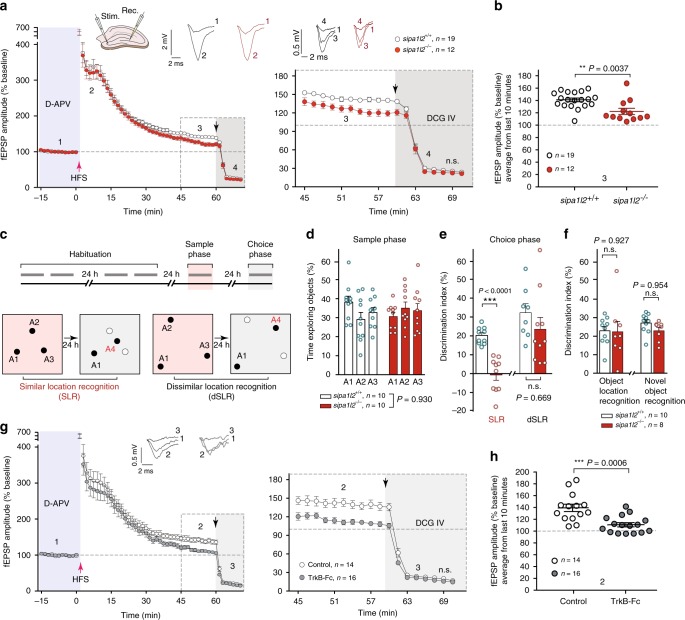
*sipa1l2*^−/−^ mice exhibit impaired MF plasticity and deficits in pattern separation. **a** MF-LTP was induced in acute slices using a high-frequency stimulus (HFS) protocol. The NMDA receptor antagonist D-APV (50 μM) was present during baseline recordings and LTP induction (shaded blue). Bath application of the group II mGluR agonist DCG-IV (2 μM) that suppresses synaptic transmission at the MF pathway was performed during the last 10 min (shaded gray) of each experiment to control input specificity. Left, the average values of fEPSP amplitudes upon MF-LTP induction. Right, fEPSP amplitudes during the last 45–70 min following MF-LTP induction. **b** Averaged fEPSP amplitudes recorded during the last 10 min of **a** before DCG-IV application (Mann-Whitney *U* test). **c** Timeline (upper panel) and schematic representation of the object distribution (lower panel) of the spatial pattern separation test. Gray bars indicate 10-min intervals. During the sample phase (red-shaded) objects (A1–3) in the similar location recognition group (SLR) were placed closer while objects in the dissimilar location recognition group (dSLR) (A1–3) were placed farther away from each other. During choice phase (gray-shaded), a new object (A4) was introduced. Animals from the SLR find A4 closer to positions A2–A3 and have a higher demand for pattern separation than those from the dSLR. Filled circles (A1–4) represent object location. Open circles indicate the absence of objects. **d** Exploration time of *sipa1l2* wt and ko animals in A1–3 during the sample phase (two-way ANOVA). **e** Discrimination index during choice phase in the SLR and dSLR groups (unpaired Student’s *t* test). **f** Discrimination index of *sipa1l2* wt and ko animals during the novel object location recognition and object recognition test (unpaired Student’s *t* test). **g**, **h** Left, average fEPSP amplitudes upon MF-LTP induction performed as explained in **a** in control and BDNF-depleted slices (TrkB-Fc, 5 µg/mL). Right, a close-up representation from the last 45–70 min. In **h**, averaged fEPSP amplitudes obtained during the last 10 min of **g** prior DCG-IV application (Mann-Whitney *U* test). Bars and error bars depict mean ± SEM in all graphs. Circles represent mean values from individual subjects (**d**–**f**) or slices (**a**, **b, g**, **h**). n.s. not significant. "**" indicates *P* ≤ 0.01; "***" indicates *P* ≤ 0.001.

To study the neuronal function of SIPA1L2, we generated *sipa1l2* ko mice (Supplementary Fig. 2a–d). No major morphological abnormalities were observed in the cerebellum and DG (Supplementary Fig. 2e–k), motor learning or coordination (Supplementary Fig. 2l–m). The number of adult-born granule cells and measures of general DG excitability and postsynaptic function were all normal in *sipa1l2*^−/−^ mice (Supplementary Figs. 2n–o and 3). However, a significant deficit was found when we assessed MF LTP, a form of plasticity presynaptically expressed at MF boutons of DG granule cells (Fig. 1a, b). Accordingly, we also observed a strong impairment in spatial pattern separation, a cognitive process associated with proper DG function and MF LTP^12–16^, that is responsible for the disambiguation and independent storage of similar memories (Fig. 1c–e). No changes were found in novel object location and recognition paradigms that are sensitive to perturbation of synaptic function in hippocampal CA1 neurons (Fig. 1f). Spatial pattern separation as well as MF LTP have been shown to depend on BDNF/TrkB signaling in the DG^11,13^ and we indeed found a reduction in MF LTP when we chelated endogenous BDNF during perfusion of slices with TrkB-Fc bodies (Fig. 1g, h), resembling the decline in late phase LTP found in *sipa1l2*^−/−^ mice.

### The cytoplasmic domain of TrkB directly binds to SIPA1L2

**Fig. 2:**
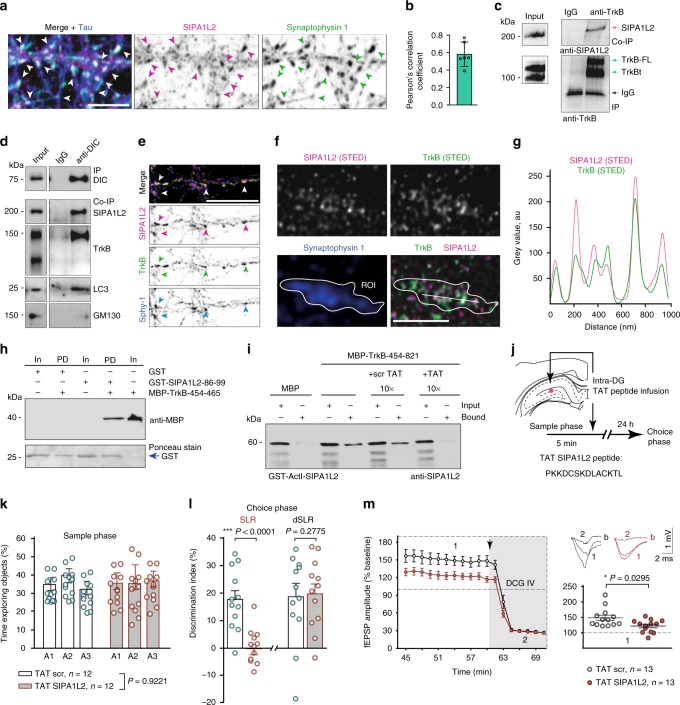
SIPA1L2/TrkB interaction at presynapses is crucial for the function of the DG. **a** Confocal images of rat hippocampal neurons immunostained against SIPA1L2, Synaptophysin 1 and Tau. Scale bar is 5 μm. **b** Pearson’s correlation coefficient calculated for SIPA1L2 and Synaptophysin 1 from **a**. Circles represent averaged values per region of interest (ROI) analyzed from three independent images. **c** Endogenous TrkB coimmunoprecipitates SIPA1L2 from rat hippocampal lysates. The two bands in TrkB correspond to the full length (TrkB-FL) and the truncated form (TrkBt). Goat anti-TrkB antibody (R&D) is used for precipitation and detection. **d** DIC coimmunoprecipitates SIPA1L2 and TrkB but not GM130 from mouse whole brain crude light membrane fraction. Note that the band revealing TrkB corresponds to the full-length form. **e** Confocal images from rat hippocampal neurons immunostained against SIPA1L2, TrkB and Synaptophysin 1 (Sphy1). Scale bar is 10 μm. **f** STED images from hippocampal neurons immunostained against SIPA1L2, TrkB and Synaptophysin 1 (confocal). Line profiles (**g**) indicate relative intensities for STED channels along 1 µm. Scale bar is 1 µm. **h** Pull-down assay between the juxtamembrane region of TrkB and the 14aa of the binding interface in ActI-SIPA1L2. **i** Pull-down assay between TrkB and ActI-SIPA1L2 in the presence of 10× TAT binding peptide (TAT) or 10× scrambled peptide (TAT-scr). **j** Cartoon representing the timeline used for the pattern separation test performed in wt animals infused with TAT peptides and the location of the infusion (red dot). **k** Exploring time from mice injected with TAT-SIPA1L2 or TAT-scr during the sample phase (two-way ANOVA). **l** Discrimination indexes obtained during the choice phase in SLR and dSLR groups injected with either TAT-SIPA1L2 or TAT-scr. The number of subjects is depicted in **k** (unpaired Student’s *t* test). **m** fEPSP amplitudes recorded during the last 45–70 min after MF-LTP upon bath perfusion of TAT-SIPA1L2 or TAT-scr peptides. Right, averaged values of fEPSP amplitudes during last 10 min of LTP recording before DCG IV application (Mann-Whitney *U* test). Bars and error bars represent data as mean ± SEM in all graphs. Circles represent mean values of individual subjects (**k**–**l**) or slices (**m**). "*" indicates *P* ≤ 0.05; "***" indicates *P* ≤ 0.001.

These experiments raised the question of whether SIPA1L2 might be involved in presynaptic BDNF-TrkB signaling. BDNF/TrkB are internalized at distal axons and transported retrogradely in a dynein-dependent manner as signaling endosomes^7,17,18^ associated with Rap1^4,8^. We found SIPA1L2 prominently present at presynaptic terminals of primary neurons labeled by the presynaptic marker Synaptophysin 1 and the axonal marker Tau (Fig. 2a, b). We, therefore, wondered whether SIPA1L2 and TrkB might be part of a single protein complex in vivo. SIPA1L2 showed an overlapping distribution with Rap1 as well as with TrkB in subcellular fractionation experiments (Supplementary Fig. 4a). Coimmunoprecipitation experiments from extracts of rat hippocampi revealed that the protein is indeed present in immunoprecipitates generated with a TrkB-specific antibody and vice versa, TrkB could be precipitated with a SIPA1L2 antibody (Fig. 2c; Supplementary Fig. 4b). Moreover, full-length TrkB and SIPA1L2 are present in transport complexes immunoprecipitated by an antibody directed against DIC (Fig. 2d) and both proteins are localized to axon terminals in hippocampal primary neurons (Fig. 2e) where they were found in very close association as revealed by STED imaging (Fig. 2f, g).

Next, we mapped the binding region in both proteins with Yeast-Two Hybrid (YTH) and found that the ActI-domain of SIPA1L2 interacts in the TrkB cytosolic domain with the first 12 juxtamembraneous amino acids (aa) (454–465) and the first 23 aa of the tyrosine kinase domain (537–559) (Supplementary Fig. 4c–d). Bacterially expressed GST- and MBP-fusion proteins revealed a direct interaction of cytoplasmic TrkB with the ActI but not the ActII domain of SIPA1L2 in pull-down assays (Supplementary Fig. 4e). We isolated 14 aa in SIPA1L2-ActI that are crucial for the association with TrkB (Fig. 2h; Supplementary Fig. 4f–h) and that happen to be unique to SIPA1L2 when compared to other SIPA1L family members (Supplementary Fig. 1c) and designed a TAT-peptide containing this region. This TAT-peptide competed for binding to TrkB in a pull-down assay (Fig. 2i) and when infused into the DG of wt animals (Fig. 2j; Supplementary Fig. 4i) induced a deficit in spatial pattern separation (Fig. 2k, l) similar to the one observed in *sipa1l2*^*−/−*^ mice (Fig. 1e). In addition, the bath perfusion of the TAT-peptide also reduced the amplitude of MF-LTP in acute slices (Fig. 2m). Thus, interruption of the SIPA1L2-TrkB interaction in wt mice mimics the deficits of *sipa1l2*^−/−^ mice.

### SIPA1L2 interacts with Snapin and enables TrkB trafficking

**Fig. 3:**
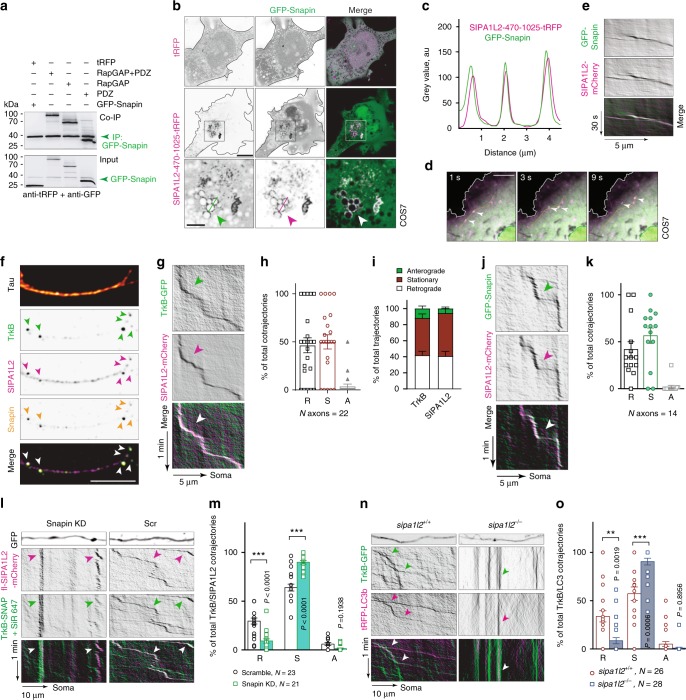
SIPA1L2 associates and cotrafficks with Snapin and TrkB. **a** GFP-Snapin coimmunoprecipitates RapGAP- and PDZ-SIPA1L2-tRFP from HEK293T cells extracts. The upper blot shows the signal from the anti-tagRFP and anti-GFP antibodies. Green arrow indicates immunoprecipitated GFP-Snapin. The lower blot represents the input. Note that the anti-tRFP antibody used also recognizes GFP. **b**, **c** Representative images (**b**) from the recruitment assay performed in COS7 cells using SIPA1L2-470-1025-tRFP or tRFP as control and GFP-Snapin. Insets are shown in the lower panel. The line profile (**c**) was generated from the line in the inset. **d**, **e** Snap-shots of fl-SIPA1L2-mCherry and GFP-Snapin cotrafficking in COS7 cells (**d**) and corresponding kymograph (**e**). Orange line in **d** represents the trajectory used for kymograph generation (**e**). **f** Confocal images from rat hippocampal neurons stained against TrkB, SIPA1L2, Snapin, and Tau. **g**, **h** Representative kymographs from neurons overexpressing TrkB-GFP and fl-SIPA1L2-mCherry. Relative motility of SIPA1L2/TrkB cotrajectories is indicated in **h**. Total number of cotrajectories is 84. **i** Relative motility of TrkB and SIPA1L2 as a percentage of total trajectories (TrkB = 284 trajectories; SIPA1L2 = 146; the number of axons is depicted in **h**). **j**, **k** Representative kymographs from neurons overexpressing GFP-Snapin and fl-SIPA1L2-mCherry. Relative motility of SIPA1L2/Snapin cotrajectories is represented in **k**. Total number of cotrajectories is 60. **l**, **m** Representative kymographs from neurons overexpressing either shRNA-based Snapin KD or Scr control together with fl-SIPA1L2-mCherry and TrkB-SNAP (+SiR647). GFP represents transfection control. Relative motility of SIPA1L2/TrkB cotrajectories is depicted in **m**. *N* = analyzed axons (nonparametric Kolmogorov-Smirnov test). **n**, **o** Representative kymographs and quantification of a percentage of TrkB-GFP/tRFP-LC3b cotrajectories in *sipa1l2*^−/−^ as compared to *sipa1l2*^+/+^ mouse hippocampal neurons. (nonparametric Kolmogorov-Smirnov test; *N* number corresponds to axons). Representative kymographs (**g**, **j**, **l**, **n**) were generated from axons of rat hippocampal neurons. Relative motility (**h**, **k**, **m**, **o**) is shown as percentage of total cotrajectories. Bars and error bars represent data as mean ± SEM. *N* number in the graph corresponds to the number of axons. Symbols in columns represent values from single axons. Arrows indicate cotrajectories. R retrograde, S stationary, A anterograde. Scale bars are 10 μm (**b**) and 5 μm (**b**, inset; **d**–**f**). "**" indicates *P* ≤ 0.01; "***" indicates* P* ≤ 0.001.

Dynein-dependent retrograde trafficking of TrkB-signaling endosomes in axons requires Snapin, an adaptor protein that recruits dynein by interacting with DIC^18^. However, whether Snapin directly or indirectly interacts with its cargo is still unclear. Heterologous coimmunoprecipitation experiments using tag-specific antibodies revealed that Snapin associates with the central region of SIPA1L2 encompassing the RapGAP and PDZ domains (Fig. 3a; Supplementary Fig. 5a). The tagged RapGAP-PDZ domain corecruits GFP-Snapin (Fig. 3b, c) and both proteins cotraffick in COS7 cells (Fig. 3d, e). Importantly, Snapin, TrkB and SIPA1L2 colocalize in axons of primary neurons (Fig. 3f) and live-imaging experiments using fluorescence-tagged proteins revealed a high percentage of cotrajectories in axons whose motility was predominantly retrograde (Fig. 3g–k; Supplementary Fig. 5b). Analysis of single TrkB and SIPA1L2 trajectories showed similar motility profiles for both proteins individually, in agreement with their cotrafficking (Fig. 3i). Of note, SIPA1L2 imaged in dendrites remained immobile (Supplementary Fig. 5c–d). We further confirmed this by shRNA-based knockdown (KD) of Snapin which reduced the motility of TrkB and SIPA1L2 and resulted in largely stationary complexes at the expense of less retrograde movement (Fig. 3l, m; Supplementary Movies 1–2). Further, TrkB was found to remain mainly stationary in *sipa1l2*^−/−^ neurons, supporting the role of SIPA1L2 in retrograde TrkB trafficking by mediating attachment to dynein via Snapin (Fig. 3n, o).

### LC3b direct association to SIPA1L2 modulates RapGAP activity

Autophagosomes are continuously generated at distal axons and are proposed to acquire Snapin and the dynein motor complex from late endosomes. The fusion of autophagosomes with late endosomes generates amphisomes and facilitates the retrograde trafficking of autophagosomes^19^. In MRC5 cells we observed a prominent colocalization of SIPA1L2 with the autophagosomal marker LC3b but not with markers for lysosomes (LAMP1) and multivesicular bodies (CHMP4B) (Supplementary Fig. 5e–f). Immunostaining of SIPA1L2, TrkB and LC3 in primary hippocampal neurons revealed extensive colocalization of all three proteins in axons (Supplementary Fig. 5g) that was further enhanced by incubation with BDNF as quantified by Manders’ coefficient calculated for protein pairs (Supplementary Fig. 5h–i). In addition, live imaging revealed largely retrograde cotrafficking of TrkB-GFP and tRFP-LC3b in a SIPA1L2-dependent manner (see above, Fig. 3n, o).

**Fig. 4:**
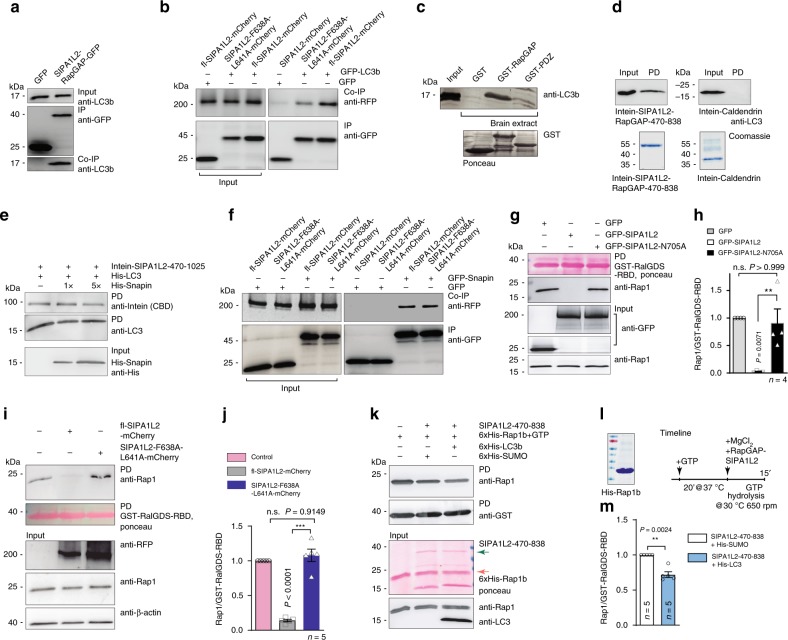
LC3 interacts with the RapGAP domain of SIPA1L2 and promotes RapGAP activity. **a** RapGAP-GFP but not GFP immunoprecipitates endogenous LC3 from a HEK293T cell extract. **b** Heterologous Co-IP from HEK293T cells showing a reduced interaction between GFP-LC3b and SIPA1L2-F638A-L641A-mCherry, which harbors a mutation within the LIR motif (FxxL/AxxA), when compared to fl-SIPA1l2-mCherry. **c** GST-RapGAP but not GST-PDZ domain of SIPA1L2 pulled down endogenous LC3 from rat brain extracts. The Ponceau staining indicates protein loading. **d** Intein-RapGAP domain of SIPA1L2 (SIPA1L2-470-838) pulls down bacterially expressed His-LC3b. Intein-Caldendrin was used as a nonrelated control. Coomassie blue staining is shown below. **e** Pull-down assay performed in the presence of Snapin. Note that an excess (5×) of Snapin does not interfere with the binding of LC3b to the RapGAP domain (SIPA1L2-470-1025). Intein tag is detected using anti-CBD antibodies against the Chitin Binding Domain of Intein and is depicted as anti-Intein (CBD). **f** Both fl-SIPA1L2-mCherry and SIPA1L2-F638A-L641A-mCherry coimmunoprecipitates GFP-Snapin from HEK293T cell extracts. **g**, **h** RapGAP activity assay shows decreased pull-down of Rap1-GTP from HEK293T cell extracts in the presence of GFP-SIPA1L2 but not GFP-SIPA1L2-N705A. The bar graph in **h** shows the quantification of the Rap1/RalGDS-RBD ratio from four independent experiments (one-way ANOVA with Bonferroni’s correction). **i**, **j** SIPA1L2-mCherry but not SIPA1L2-F638A-L641A-mCherry reduces Rap1-GTP pull-down with GST-RalGDS-RBD when expressed in HEK293T cells. Quantification of the Rap1/GST-RalGDS-RBD ratio is shown in **j** (one-way ANOVA with Bonferroni’s correction). **k**–**m** Rap-GAP activity assay performed with recombinant SIPA1L2-470-838 in the presence of His-LC3b or His-SUMO as a negative control. In **l**, time line of the RapGAP activity assay and purity of His-Rap1b confirmed by SDS-PAGE and Coomassie  blue staining. In **m**, quantification of the Rap1-GST-RalGDS-RBD ratio shows the effect of LC3b on enhancing RapGAP activity of SIPA1L2 (paired Student’s *t* test). Bars and error bars represent data as mean ± SEM. *N* number in the graph corresponds to number of independent experiments. n.s. not significant. "**" indicates* P* ≤ 0.01; "***" indicates* P* ≤ 0.001.

Interestingly, the RapGAP domain of SIPA1L2 contains a LIR-motif (FxxL-motif) for LC3-binding that is highly conserved in mammals and among all SIPA1L family members (ref. ^20^; Supplementary Fig. 1c). In endogenous coimmunoprecipitation experiments performed with an anti-DIC antibody to isolate transported complexes, we found that LC3 coprecipitates with SIPA1L2 and TrkB (Fig. 2d). In addition, heterologous coimmunoprecipitation experiments conducted with tag-specific antibodies revealed an interaction of the RapGAP domain with endogenous LC3 in HEK293T cell lysates (Fig. 4a; Supplementary Fig. 5a). Binding to LC3 was weaker when we expressed SIPA1L2 carrying point mutations within the LIR motif (Fig. 4b). Moreover, the RapGAP domain fused to GST efficiently pulled down endogenous LC3 from a rat brain lysate (Fig. 4c). The catalytically active RapGAP domain of SIPA1L2 (ref. ^21^; aa 470–838; Supplementary Figs. 1d; 5j–k) also showed a direct interaction with bacterially expressed His-LC3b in a pull-down assay (Fig. 4d) and this interaction was not affected by the inclusion of Snapin in the pull-down buffer (Fig. 4e). Reciprocally, the association with Snapin was not reduced by mutation of the LIR motif (Fig. 4f). Thus, it seems that both Snapin and LC3b concomitantly bind to SIPA1L2 and that the presence of Snapin does not prevent the binding of SIPA1L2 to LC3b.

In the next set of experiments, we determined whether the binding of LC3b might modulate RapGAP activity. To this end, we extracted SIPA1L2 expressed in HEK293T cells and pulled down endogenous GTP-bound Rap1 with RalGDS-RBD coupled to GST-matrix. As a negative control, we used an SIPA1L2 mutant carrying a point mutation within the Asn-thumb (SIPA1L2-N705A) that renders the RapGAP domain inactive (Fig. 4g, h; Supplementary Fig. 1c). RapGAP activity of SIPA1L2 was abolished when we expressed the SIPA1L2 LIR-mutant (Fig. 4i, j). On the contrary, RapGAP activity was enhanced in the presence of recombinant His-LC3b (Fig. 4k–m). Collectively, these data suggest that the interaction of LC3b with the RapGAP domain modulates the catalytic activity of SIPA1L2.

### SIPA1L2 controls retrograde trafficking of TrkB/LC3b

**Fig. 5:**
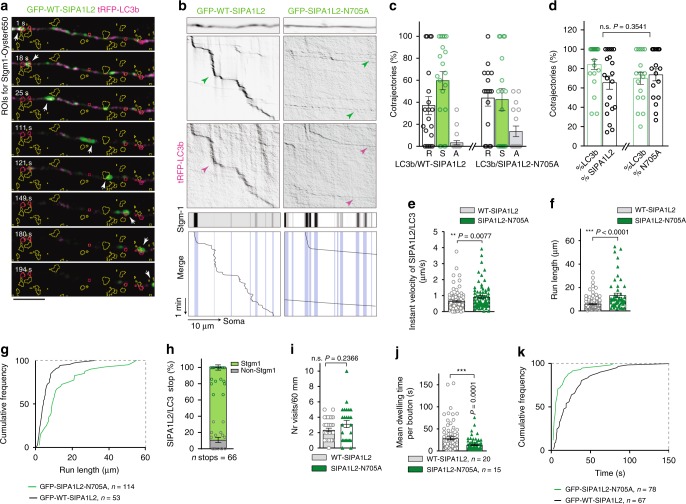
The RapGAP activity of SIPA1L2 controls the motility of SIPA1L2-amphisomes. **a** Time-lapse representation of GFP-SIPA1L2/tRFP-LC3b visiting presynaptic boutons from rat primary hippocampal neurons live-labeled by Stgm-1^Oyster650^ (yellow ROIs). Note that only ROIs in red are within the axon of interest and considered for analysis. Imaging was performed in conditioned neuronal media and three-channel time-lapse acquired for 5 min. Scale bar = 5 μm. **b** Representative kymographs from the experiment described in **a**. Neurons expressed tRFP-LC3b and GFP-SIPA1L2 or GFP-SIPA1L2-N705A. Below, stationary Stgm-1^Oyster650^ indicates presynaptic boutons. Sketch drawing represents traces of cotrajectories aligned with positions of presynaptic boutons (shaded blue lines). **c**, **d** Relative motility of SIPA1L2/LC3b and SIPA1L2-N705A/LC3b cotrajectories as percentage of total cotrajectories shows predominantly retrograde (R) movement (S, stationary; A, anterograde). In **d**, cotrajectories showed as a percentage of total LC3b or SIPA1L2 trajectories. Circles in bar graphs show values per analyzed kymograph (GFP-SIPA1L2: *n* = 20 axons, GFP-SIPA1L2-N705A: *n* = 17 axons). One-way ANOVA with Bonferroni’s posthoc test. **e** Instant velocity (μm/s) of GFP-SIPA1L2/tRFP-LC3b and GFP-SIPA1L2-N705/tRFP-LC3b cotrajectories. GFP-SIPA1L2: *n* = 102 instant measures from 20 axons; GFP-SIPA1L2-N705A: *n* = 77 instant measures from 14 axons. Mann−Whitney *U* test for the number of instant measures. **f**, **g** Quantification (**f**) and cumulative distribution (**g**) of the run-length; GFP-SIPA1L2: *n* = 114 measures from 20 axons; GFP-SIPA1L2-N705A: *n* = 53 measures from 14 axons (Mann-Whitney *U* test for the number of measures). **h** Percentage of LC3b/SIPA1L2 stopovers occurring in presynaptic boutons labeled by a Synaptotagmin 1 (Stgm1) antibody shows the preferential occurrence of these stopovers at boutons. Stops occurring outside Stgm1 labeling are depicted as non-Stgm1. Numbers of stops from 13 axons is depicted under the graph. **i** Average number of visited boutons in 60 μm axon lengths of SIPA1L2/LC3b and SIPA1L2-N705A/LC3b vesicles. GFP-SIPA1L2: *n* vesicles = 21 from 20 axons; GFP-SIPA1L2-N705A: *n* vesicles = 21 from 14 axons cultures (Mann-Whitney *U* test for number of vesicles). **j**, **k** Mean synaptic dwelling time (GFP-SIPA1L2: *n* = 78 stops from 20 axons; GFP-SIPA1L2-N705A: *n* = 67 stops from 15 axons) calculated from imaging of neurons in conditioned neuronal media (Mann-Whitney *U* test for *n* of stops). Data in bar graphs are depicted as mean ± SEM. n.s. - not significant. "**" indicates *P* ≤ 0.01; "***" indicates *P* ≤ 0.001.

Rap signaling is necessary for sustained activation of ERK^22–24^ which in turn has been shown to recruit Dynein and to promote retrograde trafficking of TrkB^25^. Therefore, we asked whether the RapGAP activity of SIPA1L2 might modulate trafficking of the complex. Live-imaging of rat hippocampal neurons confirmed cotrafficking of LC3b and SIPA1L2 in a predominantly retrograde direction (Fig. 5a–c). LC3b trajectories were in the majority of cases positive for SIPA1L2 (Fig. 5d). A SIPA1L2-RapGAP dead mutant that is incapable of terminating Rap-signaling also showed cotrajectories with LC3b (Fig. 5c, d). However, under these conditions, the instant velocity and the run-length of the SIPA1L2-LC3b complexes were significantly enhanced (Fig. 5b, e–g), in agreement with an increased ERK activation and recruitment of Dynein^25^. Because of the effect of the RapGAP activity on the run-length of the complex, we hypothesized that the SIPA1L2/LC3b stops might occur at presynaptic boutons and that this might be controlled by the RapGAP activity of SIPA1L2. Live-imaging of short axonal segments (30–40 µm) where active presynaptic terminals were identified using Synaptotagmin1^Oyster650^ live-antibody labeling demonstrated that SIPA1L2/LC3b complexes preferentially stopped at single, active boutons (Fig. 5a, b, h). While no changes in the number of visited boutons per axonal segment between WT- and RapGAP-dead SIPA1L2/LC3b trajectories was observed (Fig. 5i), we found that the lack of RapGAP activity strongly diminished the dwelling times of SIPA1L2/LC3b at presynaptic boutons (Fig. 5j, k). Taken together, these data indicate that the RapGAP activity of SIPA1L2 controls the trafficking of the complex which retrogradely trafficks stopping at synaptic boutons.

### cLTP prolongs the stopovers of TrkB-amphisomes at boutons

**Fig. 6:**
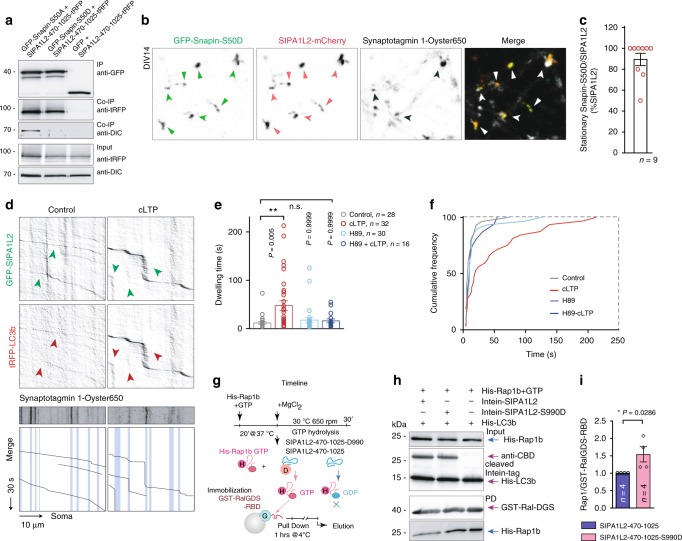
cLTP prolongs dwelling time of SIPA1L2-amphisomes at presynaptic boutons. **a** Heterologous coimmunoprecipitation experiments using SIPA1L2-470-1025-tRFP (RapGAP-PDZ) and a phospho-deficient (GFP-Snapin-S50A) or a phosphomimetic Snapin mutant (GFP-Snapin-S50D). Both forms of Snapin coimmunoprecipitates with SIPA1L2, but only the phospho-deficient form of Snapin coimmunoprecipitates with DIC. **b**, **c** Snapshots in **b** obtained from time-lapse imaging in rat hippocampal cells overexpressing GFP-Snapin-S50D together with fl-SIPA1L2-mCherry where mostly immobile (**b**, arrows) vesicles were found at presynaptic terminals labeled with anti-Synaptotagmin 1-Oyster650 (black arrows). Scale bar is 10 μm. In **c** quantification of the percentage of stationary cotrajectories (note the difference with Fig. 3k). **d**–**f** Kymographs generated from axons coexpressing GFP-SIPA1L2 and tRFP-LC3b and labeled in vivo with Stgm-1^Oyster650^ after control or chemical long-term potentiation (cLTP) induction. Imaging was performed in nonconditioned, extracellular imaging buffer. Below, merge images represent traces of cotrajectories aligned with positions of presynaptic boutons (shaded blue lines). Dwelling time of the SIPA1L2-LC3b-amphisomes at boutons is represented in **e** and corresponding cumulative distribution diagram in **f**. Data represented as mean ± SEM. *N* numbers in **e**, **f** correspond to vesicles analyzed from 11 axons (control), 14 axons (cLTP), 8 axons (H89) and 6 axons (H89 + cLTP) (one-way ANOVA on ranks with Dunn’s multiple comparison test for vesicles). **g**–**i** In **g**, the timeline for the RapGAP activity assays performed in **h**, **i**. Recombinant intein-tagged SIPA1L2-470-1025 as well as SIPA1L2-470-1025-S990D (phosphomimetic SIPA1L2 mutant in a potential PKA phosphosite) were used to hydrolize recombinant Rap1b loaded with GTP in the presence of LC3b. Quantification of four independents experiments in **i** (Mann-Whitney *U* test). Bar graphs depict data as mean ± SEM. "*" indicates* P* ≤ 0.05; "***" indicates* P* ≤ 0.001.

Presynaptic LTP results in activation of PKA at MF boutons and enhanced synaptic function that is largely mediated by PKA-dependent phosphorylation of different components of the presynaptic release machinery^26–30^. PKA-mediated phosphorylation of Snapin at Ser50 is crucial for its dissociation from the DIC and a phosphomimetic Snapin-S50D mutant is largely immobile^31^. Heterologous coimmunoprecipitation experiments revealed that both phosphomimetic (S50D) as well as phospho-deficient (S50A) Snapin interact with tRFP tagged SIPA1L2-470-1025 (Fig. 6a). However, in contrast to the phospho-deficient protein, DIC did not coimmunoprecipitate with phosphomimetic Snapin (Fig. 6a), indicating that PKA-dependent phosphorylation induces the dissociation of the protein from DIC. Consistent with previous work^31^, time-lapse imaging revealed that phosphomimetic Snapin is largely stationary and was often found in proximity to immobile fl-SIPA1L2-mCherry at axon terminals (Fig. 6b, c). We therefore next wondered whether changes in synaptic activity could affect amphisome trafficking among presynaptic terminals. Trafficking of SIPA1L2/LC3b among presynaptic terminals labeled by anti-Synaptotagmin 1^Oyster650^ antibodies revealed an enhancement in dwelling time upon induction of chemical LTP (cLTP) (Fig. 6d–f). The PKA inhibitor H89 prevented this effect (Fig. 6e, f).

It has been reported that PKA-dependent phosphorylation of Ser499 of Rap1GAP negatively regulates RapGAP activity^32^. Sequence analysis of SIPA1L2 revealed a high scoring PKA phosphorylation motif (S990-RxxpS motif/ 0,757/ Supplementary Fig. 1d) that is located close to the region that corresponds to the regulatory PKA sites (pSer 499) of Rap1GAP and that is well preserved between SIPA1L family members (Supplementary Fig. 1c). When we tested the hypothesis that PKA in analogy to Rap1GAP might negatively regulate RapGAP activity of SIPA1L2, we found that phosphomimetic SIPA1L2-470-1025-S990D indeed hydrolyzed very little recombinant Rap1b-GTP (Fig. 6g–i), indicating a negative regulation of RapGAP activity by PKA. Collectively these data suggest that following induction of presynaptic plasticity, PKA phosphorylation of Snapin induces the dissociation of the amphisome from Dynein and enhances its residing time at presynaptic boutons. Concomitant phosphorylation of SIPA1L2 diminishes its RapGAP activity and thereby potentially facilitates ERK signaling.

### Amphisomes activate ERK at boutons and enhance release

**Fig. 7:**
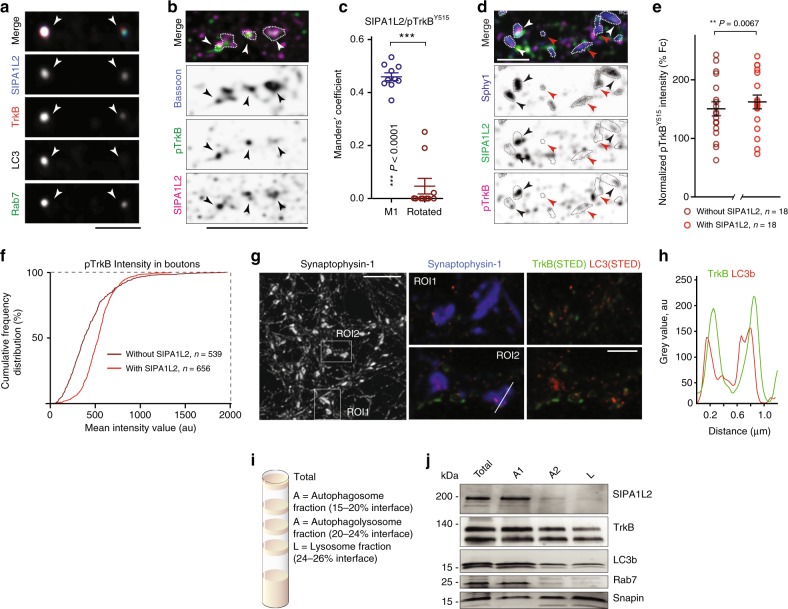
TrkB-LC3b-Rab7-containing amphisomes are positive for SIPA1L2. **a** Confocal images of quadruple immunofluorescence performed in rat primary hippocampal neurons stained for SIPA1L2, LC3, TrkB with Rab7. Scale bar is 1 μm. **b** Confocal images of primary neurons treated with BDNF and stained for pTrkB^Y515^, SIPA1L2, and Bassoon show colocalization of SIPA1L2 and pTrkB ^Y515^ in presynaptic boutons. Scale bar is 5 µm. **c** Mander’s coefficient calculated for pTrkB^Y515^ and SIPA1L2 in boutons detected by Bassoon staining. For control measurements, the same images were rotated 90º to the right (paired Student’s *t* test). **d**, **e** Representative images in **d** of rat hippocampal neurons treated with BDNF and stained for pTrkB^Y515^, SIPA1L2 and Synaptophysin1. Arrows indicate boutons where SIPA1L2 is present (black) or absent (red). Note that in the presence of SIPA1L2, pTrkB^Y515^ intensity is higher compared to those from boutons where SIPA1L2 is absent. Quantification is shown in **e**, circles represent averaged intensities per image (paired Student’s *t* test for average per image). Averaged intensities are normalized to images acquired from Fc-TrkB-treated neurons. The cumulative frequency distribution is shown in **f** (*n* = synaptic boutons). Scale bar is 2 μm. **g**, **h** Super-resolution STED imaging performed in rat hippocampal neurons revealed association of TrkB with LC3 at the presynaptic boutons. In **h**, line profiles from the line in the ROI2 in **g**. Scale bars 5 µm (overview) and 1 µm (inserts). **i**, **j** In **i**, scheme depicting fractionation protocol performed from rat brain that results in autophagosome (A1), autophagolysosome (A2) and lysosome (L) fraction. Note that Rab7 and SIPA1L2 are only present in the total and A1 fraction (**j**) according to their presence in amphisomes but not in later stages of the autophagosomal pathway. Lines in dot plot graphs depict data as mean ± SEM. "**" indicates *P* ≤ 0.01; "***" indicates *P* ≤ 0.001.

Amphisomes are believed to be transient intermediate organelles that in nonneuronal cells rapidly enter a degradative lysosomal pathway. However, lysosomes are not abundant if at all present in distal axons^33^. In primary cultures, STED imaging revealed that the lysosomal marker LAMP1 was indeed mostly absent from presynaptic boutons, rendering unlikely that SIPA1L2-TrkB-LC3b associates with a degradative organelle at axon terminals (Supplementary Fig. 6a). A recent study showed that TrkB is transported on autophagosomes to the soma where it regulates gene expression^34^. This transport requires association of the Clathrin adaptor AP2 with LC3 and DIC^34^. Therefore, we speculated that amphisomes could constitute more persistent entities in axons where they could serve signaling functions at presynaptic boutons. Accordingly, we found that SIPA1L2/LC3/TrkB complexes are positive for the late endosome marker Rab7 (Fig. 7a; Supplementary Fig. 6b–c). Moreover, kinase-active pTrkB^Y515^ showed a significant degree of colocalization with SIPA1L2 at presynaptic boutons in line with the high association of LC3, SIPA1L2 and TrkB observed before (Fig. 7b, c). This is expected to be higher when assessing colocalization in only those boutons in which SIPA1L2 is present. Accordingly, intensities of pTrkB^Y515^ are higher in SIPA1L2-positive boutons (Fig. 7d–f). This tight association of SIPA1L2 with pTrkB^Y515^ as well as TrkB with LC3 (Fig. 7g, h) and Rap1 with SIPA1L2 and LC3 (Supplementary Fig. 6d) at presynapses indicate that SIPA1L2 might be part of a signaling TrkB amphisome. We also found an extensive colocalization with AP2 in axons and synaptic boutons (Supplementary Fig. 6e–g), suggesting that SIPA1L2-TrkB amphisomes are long-range organelles that will traffic retrogradely to reach the soma^34^. Moreover, TrkB and SIPA1L2 associate with an autophagosome enriched fraction from rat brain lysates that is also positive for LC3, Rab7 and Snapin (Fig. 7i, j). Of note, SIPA1L2 was not found in the lysosome fraction (Fig. 7i, j).

**Fig. 8:**
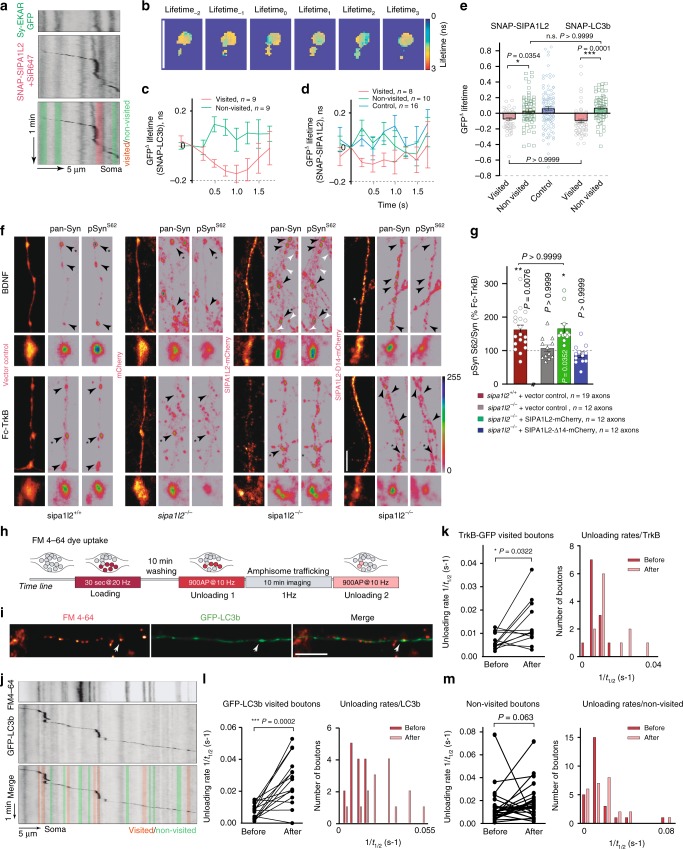
SIPA1L2-amphisomes activate ERK at boutons and potentiate presynaptic function. **a** Kymographs represent the visit of SIPA1L2-amphisome labeled by SNAP-SIPA1L2 (+SiR647) to a bouton identified by Sy-EKAR. **b** Representative heat maps depicting GFP lifetime (n.s.) over time. Lifetime_0_ represents the frame when the amphisome reaches the bouton. Scale bar is 5 μm. **c**, **d** Quantification of GFP-∆Lifetime over time in visited and nonvisited boutons. Amphisomal arrival is considered as time 0. Control in **d** represents data from axons where no trajectories were found. *N* numbers represent analyzed boutons from *n* > 3 independent experiments. **e** Averaged GFP-∆Lifetimes from **c**, **d**. One-way ANOVA with Bonferroni’s posthoc test; *n* = number of boutons. **f** Representative confocal images from wt and *sipa1l2*^*−/*−^ primary neurons overexpressing fl-SIPA1L2-mCherry, SIPA1L2-Δ14-mCherry or mCherry, treated with TrkB-Fc or BDNF and immunostained for Synapsin1,2 (Syn) and phospho-Syn^S62^. White arrows show nontransfected boutons. Insets depict boutons labeled with the asterisk. Scale bar is 5 µm. **g** Percentage of enhancement of the pSyn^S62^/Syn ratio in BDNF-treated as compared to Trk-Fc-treated neurons. *P* values correspond to comparisons with Fc-TrkB-treated cells for the corresponding condition (Kruskall-Wallis test with Dunn’s correction; wt-tRFP-Fc-TrkB = 20 axons; ko-tRFP-Fc-TrkB = 13 axons; ko-SIPA1L2-Fc-TrkB = 10 axons; ko-SIPA1L2d14-Fc-TrkB = 15 axons; two independent experiments). **h** Scheme showing the timeline of the experiment: FM_4-64_ (10 µM) was loaded in the terminals by a train of pulses (30 s @ 20 Hz) and washed out for 10 min. First FM_4-64_ unloading was done by delivering 900 pulses@10 Hz. Trafficking of TrkB-GFP or GFP-LC3b was imaged for 10 min before a second unloading protocol was applied. **i** Representative images showing an axon overexpressing GFP-LC3b after loading with FM4-64 (red). Scale bar is 10 μm. **j** Kymographs prepared from an axonal segment overexpressing GFP-LC3b and loaded with FM4-64. **k**–**m** Unloading rate after visits of TrkB-GFP (**k**) or GFP-LC3b (**l**) and from nonvisited boutons (**m**). Dots indicate boutons. Right panels show the frequency distribution of unloading rates (paired Student’s *t* test in **k**, **l** and Wilcoxon rank test in **m**). Shaded lines in kymographs (**a**, **j**) represent visited (red) and nonvisited (green) boutons. Bar graphs show mean ± SEM. n.s. stands for not significant. "*", "**", "***" indicate *P* ≤ 0.05, *P*≤0.01, *P* ≤ 0.001, respectively.

What could be the consequence of amphisome stopover at axon terminals? Local ERK activation has been linked to enhanced neurotransmitter release and presynaptic plasticity^35,36^. We took advantage of a FRET-based ERK sensor^37^ that we targeted to the presynaptic compartment following fusion to Synaptophysin-1 (Fig. 8a–e; Supplementary Fig. 6h). We then imaged changes in the GFP fluorescence lifetime, the donor of the FRET pair, coinciding with the stopover of amphisomes, which we identified using either tagged versions of SIPA1L2 or LC3b. Synaptic visits were determined by kymograph analysis (Fig. 8a). Quantification of ∆Lifetime_GFP_ upon the arrival of the amphisome at the bouton (time 0) indeed correlated with a significant decrease in the GFP lifetime (Fig. 8b–e), indicating a higher FRET efficiency due to ERK activation and subsequent phosphorylation of the sensor^37^. Importantly, this decrease was not observed when the amphisome passed by a bouton without stopping (nonvisited terminals) or in axons where no amphisomal trafficking was observed during the recording time (Fig. 8c–e).

ERK activation in presynaptic plasticity results in phosphorylation of Synapsin I (Syn-I), a protein known to play a role in BDNF-mediated increase of neurotransmitter release^36^. Ultrastructural analysis revealed no major alterations in presynaptic bouton organization and vesicle content in *sipa1l2*^−/−^ as compared to wt mice (Supplementary Fig. 6i–j). Quantification of ERK-dependent phosphorylation of Syn-I in hippocampal primary neurons from wt mice revealed a significant enhancement of the pSyn/Syn ratio at boutons following BDNF application (Fig. 8f, g) without changes in their number (Supplementary Fig. 6k). The BDNF-induced enhancement of the pSyn/Syn ratio was absent in *sipa1l2*^−/−^ neurons and could be rescued by re-expression of fl-SIPA1L2 in ko neurons. However, no rescue was observed when we re-expressed the SIPA1L2-Δ14 mutant lacking the TrkB binding region (Fig. 8f, g).

To directly assess the effects of the activation of these molecular pathways in transmitter release, we combined unloading of FM 4-64 dyes to monitor synaptic vesicle fusion with live imaging of LC3b/TrkB trafficking and compared unloading rates at visited and nonvisited boutons (Fig. 8h–j). Analysis of the unloading rates before and after a stopover revealed a significant enhancement in the majority of the analyzed boutons visited by both TrkB-GFP (Fig. 8k) and GFP-LC3b (Fig. 8l). Importantly, this increase was not observed in nonvisited neighboring boutons (Fig. 8m).

## Discussion

**Fig. 9:**
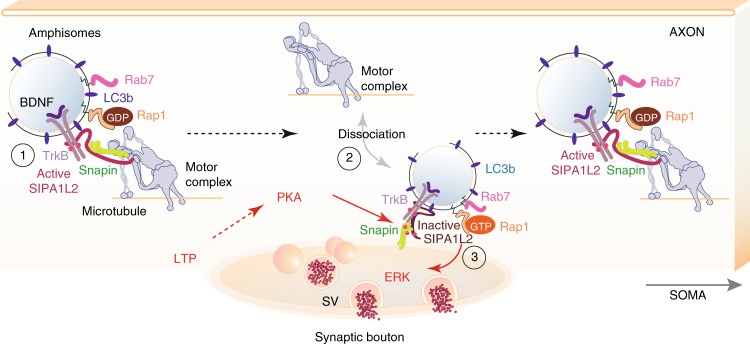
SIPA1L2 controls trafficking and signaling of TrkB-amphisomes at boutons. Signaling amphisomes result from the fusion of active TrkB containing late endosomes with autophagosomes (1). Retrograde trafficking and signaling of these amphisomes in axons are tightly regulated by SIPA1L2, which directly binds to TrkB, Snapin, and LC3b. While the binding to Snapin serves as a linker to a dynein motor, binding of LC3b enhances SIPA1L2 RapGAP activity which negatively interferes with TrkB/Rap1-signaling and also slows down the velocity of retrograde transport. (2) The amphisome halts at single presynaptic boutons in a PKA-dependent manner. PKA-dependent phosphorylation of Snapin triggers its dissociation from the motor complex and immobilizing the amphisome at individual axon terminals. (3) PKA activity also terminates RapGAP activity by phosphorylating SIPA1L2 and allows TrkB/Rap1 signaling that subsequently activates synaptic ERK and facilitates transmitter release.

Autophagosomes in neurons are formed continuously in distal axon terminals^38,39^ from where the autophagic flux of cargoes derived from synapses is directed towards the soma in a dynein-dependent manner^19,38–42^. Classical stimuli like starvation have relatively little effect on neuronal autophagic flux^40^. However, it was shown previously that synaptic activity transiently upregulates autophagy at presynaptic terminals^41,43–45^ and autophagosome formation might be therefore regulated locally in individual boutons. Although there is growing appreciation that neuronal autophagy might serve synaptic function^46–48^, it has not been shown yet that it might be a mechanism used to mediate activity-dependent synaptic change. In the present work, we introduce a signaling mechanism that is based on the incorporation of TrkB receptors from late endosomes to autophagosomes. This fusion has been largely associated with the termination of signaling^49^ and evidence for the existence of a signaling amphisome was scarce^50^. We found that the resulting organelle has indeed features of an amphisome with signaling properties whose trafficking and signaling capabilities are tightly controlled by SIPA1L2 and its association with TrkB, Snapin, and LC3. Intriguingly, binding of LC3 enhances RapGAP activity and thereby negatively interferes with TrkB-induced Rap signaling, which is upstream of ERK activation (Fig. 9). PKA phosphorylation of Snapin and SIPA1L2 at presynaptic sites immobilizes the amphisome at axon terminals, terminates RapGAP activity and thereby allows TrkB/Rap1 signaling that will facilitate transmitter release (Fig. 9). It was proposed previously that autophagosomes contain active TrkB complexes^34^. In this study, we provide evidence that TrkB signaling endosomes are in fact amphisomes whose formation is likely linked to the regulation of neuronal autophagy. We propose that neuronal amphisomes are stable prelysosomal hybrid organelles with signaling capabilities based on the findings that (i) they were immunopositive for the late endosome marker Rab7 but not the lysosomal marker LAMP1, (ii) TrkB in presynaptic boutons is invariably found in close proximity to SIPA1L2 and phosphorylated at Y515, which is a crucial phosphosite for ERK signaling, (iii) amphisome stopover occurred predominantly at active release sites, (iv) amphisome trafficking to boutons correlated with enhanced levels of phosphorylated Syn-I and ERK activation and (v) finally, enhanced transmitter release.

What could be the advantage of assembling a stable hybrid organelle for signaling at axonal boutons? Axon terminals and distal axons in mature neurons contain very few lysosomes and acidification and proteolytical cleavage are significantly delayed until amphisomes reach the soma^33,39^. Most important, whereas the lumen of autophagosomes becomes more acidified during retrograde transport and lysosomal markers accumulate, levels of cathepsins, the major lysosomal proteases, remain very low compared to those in the soma^51^. If one takes into account the extreme distances in axonal transport, amphisomes will constitute stable entities long before acquiring a lysosomal identity near the neuronal cell body. Since autophagosomes fuse with late endosomes to undergo robust retrograde transport^19^, it is probably inevitable that, in the absence of autolysosome formation, amphisomes serve as signaling and sorting platforms while trafficking in a retrograde direction. This makes endosomal sorting processes at axon terminals dispensable and would give an answer to the long-standing question of why neurons transport autophagic and endocytic cargos back to the cell body for degradation instead of disposing of them locally.

It is likely that amphisomes collect additional cargo during visits at synapses that may differently determine their properties and vesicular fate while propagating retrogradely to the soma. Sustained signaling capacity and diversity will promote and integrate local synaptic function with long-range signaling while evading lysosomal degradation. Specificity for certain synaptic connections is conceivable and in the case of SIPA1L2 might come from docking to presynaptic sites via for instance PDZ domain-dependent interactions. The idea of selective amphisome signaling at subpopulations of boutons is appealing since TrkB/BDNF facilitation of presynaptic transmitter release possibly requires more sophisticated signaling than the one provided by the classical TrkB signaling endosomes that lack local signaling function and selectivity for individual boutons required to induce clustered presynaptic plasticity like it was proposed by Staras et al.^52^. This study demonstrated the existence of a vesicle pool that is not confined to a synapse but spans multiple terminals. Vesicles within this superpool are highly mobile and are rapidly exchanged between terminals. Focal BDNF application suggests the involvement of a local TrkB-receptor-dependent mechanism for synapse-specific regulation of presynaptic vesicle pools through control of vesicle release and capture to or from the extrasynaptic pool^52^. Thus, clustered presynaptic plasticity is another conceivable function of amphisome signaling. This is also interesting in light of several studies that have shown that MF LTP is expressed presynaptically and requires cAMP–PKA signaling^53^. Our results suggest that the SIPA1L2 amphisome is a likely substrate for PKA-dependent LTP. Along these lines, we speculate that at MF synapses SIPA1L2-related amphisome signaling is important for the maintenance of LTP, which in turn requires BDNF/TrkB signaling and is important for pattern separation.

Several adaptors have been described for antero- and retrograde transport of TrkB^18,34,54–57^. Many of these adaptors show also an association with autophagosomes^58–60^ and synergies likely exist between adaptors. Interestingly, Snapin is reportedly also an adaptor for axonal lysosomes^59^, which further supports the idea of a single transport complex slowly acquiring a lysosomal identity during retrograde movement. We could show that SIPA1L2-mediated ride-on service for TrkB-containing amphisomes enables local and long-distance signaling (Fig. 9). In this context, it should be mentioned that other SIPA1L family members exhibit a similar domain organization with a high degree of similarity in the RapGAP, PDZ and CC-leucine zipper domain (Supplementary Fig. 1). It is thus very likely that all of them will bind Snapin and LC3 while they might differ in cargo selection for retrograde transport of amphisomes but also additional functions of the SIPA1L family in autophagy are conceivable.

## Methods

### Animals

Animals were maintained in the animal facility of the Leibniz Institute for Neurobiology, Magdeburg (Germany) or ZMNH, Hamburg (Germany) under controlled environmental conditions.

All animal experiments have complied with all ethical regulations for animal testing and research in accordance with the European Communities Council Directive (2010/63/EU) and were approved by the ethics committees of Sachsen-Anhalt/Germany (reference number 42502-2-1264 LIN and 42502-2-1284 UNIMD) or of the city-state of Hamburg (Behörde für Gesundheit und Verbraucherschutz, Fachbereich Veterinärwesen) and the animal care committee of the University Medical Center Hamburg-Eppendorf.

### Generation of *sipa1l2*^*−/−*^ mice

For a constitutive knockout generation, exon 2 of the *SPAR2/sipa1l2* locus (Gene ID: 244668; RefSeqs NM_001081337.1 (mRNA), NP_001074806.1 (protein)) was replaced by a IRESLacZ reporter gene and a neomycin phosphoribosyltransferase selection cassette by homologous recombination. The targeting vector was constructed by amplifying homologous regions from 129S6/Sv/Ev mouse genomic DNA using primers described in Supplementary Table 1.

Gene targeting was performed using ES cells (CCB; 129S6/Sv/Ev strain) and chimeras were generated by injection into C57/Bl6 blastocysts. All experiments described here were performed with mice backcrossed for at least ten generations to C57BL/6J background. Genotyping was performed with primers described in Supplementary Table 1 resulting in bands of 200 bp for wt and 400 bp for ko condition.

### Antibodies

A list of antibodies used in this study and corresponding dilutions is available in Supplementary Table 1.

### Cloning

Sources of the main constructs and primers used in this study are available in Supplementary Tables 1 and 2. For Snapin knockdown experiments, a published Snapin KD sequence^61^ was cloned into the pSIH-H1 shRNA vector (System Biosciences). Inserted sequences are described in Supplementary Table 1.

The Sy-EKAR vector was created by PCR amplification of EKAR-GFP/RFP (ref. ^37^; Addgene #18680) to which a 4Gly linker domain (GGTGGCGGTGGA) was incorporated in the N-terminus (see Supplementary Table 1). This was subcloned into the CMV: ratSyGCaMP2 (Addgene #26124), from where GCaMP2 was removed. SIPA1L2 mutants were generated using the Q5 site-directed mutagenesis kit (NEB).

### Yeast Two-Hybrid

The Yeast-Two-Hybrid assay was performed as described in ref. ^62^.

### Electrophysiology in acute hippocampal slices

MF-LTP induction was performed as described in ref. ^11^. Hippocampal slices from 11–16-week-old C57BL/6J or *sipa1l2*^−/−^ mice were cut and preincubated for 3 h prior recordings. When required, slices were preincubated for 3 h before recording in carbogenated ACSF either with 1 µM of TAT-SIPA1L2 peptide or with 1 µM of TAT-scrambled peptide as a control, as well as with or without 5 µg/ml TrkB-Fc.

For assessment of postsynaptic DG LTP, field-EPSPs were measured with an electrode positioned at the middle of the molecular layer of the DG. The medial perforant path was stimulated with biphasic constant current pulses (0.1 ms per half-wave duration). Different theta-burst stimulation protocols (TBS) or a high- frequency stimulation (HFS) protocol were used for induction of synaptic plasticity. The TBS protocols consisted of either one episode of brief presynaptic bursts (ten pulses of 0.2 ms per half-wave duration delivered @10 Hz) repeated ten times @5 Hz, two of these episodes (inter-episode interval 10 s) or four episodes (inter-episode interval 10 s) repeated four times every 5 min. The HFS protocol consisted of four trains of 100 pulses (0.2 ms per half-wave duration) @100 Hz, inter-train interval 5 min.

### Behavioral tests

The behavioral spatial pattern separation task was performed as described^13^ with minor modifications. For object location and novel object recognition task, two equal objects were placed at the equidistant position from the walls of the arena, and the animals were free to explore them. Twenty-four hours later, novel location recognition was assessed as one of the same familiarized objects was displaced to a new location and mice were again left free to explore both objects over 20 min. On the third day, a novel object recognition test took place as one of the familiarized objects was now exchanged for a novel object. Animals were again left for 20 min to explore the objects before being returned to the home-cage. In experiments involving rotarod, animals were tested on an accelerating rotarod apparatus two trials per day, for 5 consecutive days, in which the speed of the rod was gradually increased each day (from 15 to 36 rpm). On day 5, each animal was submitted to four trials lasting 300 s each, during which the rotation speed gradually increased from 4 to 40 rpm. The latency to fall was recorded.

In experiments requiring infusion of TAT-peptides in DG, animals (wt) underwent sample phase training of the spatial pattern separation protocol, and 5 min after the session received an intra-DG infusion of either 1 mM TAT-SIPA1L2 peptide or TAT-scrambled conjugated with fluorescin. Anesthesia was induced at 5% isofluorane in O_2_/N_2_O mixture (Rothacher Medical GmbH., Switzerland) and mice were placed in the stereotaxic frame (World Precision Instruments). During surgery, anesthesia was maintained at 1.5–2.0%. Craniotomy was done targeting the dorsal dentate gyrus with stereotaxic coordinates, anterioposterior (AP) −2.0 mm, mediolateral (ML) ±1.4 mm from Bregma and dorsoventral (DV) −1.6 mm (from brain surface). Each mouse received 1.5 μl bilateral infusion of a respective peptide at a flow rate of 0.5 μl/min. The effect of TAT-peptide infusion was assessed 24 h later in the choice phase of pattern separation task. After the completion of the task, mice were deeply anesthetized and perfused transcardially with PBS and 4% paraformaldehyde (PFA) in PBS solution to verify DG localization of injections.

### Cell culture, transfections and immunostaining

Maintenance and transfection of MRC5, HEK293T and COS-7 cells were performed as described in ref. ^63^. HEK293T were obtained from Leibniz Institute DSMZ (Cat#ACC635; RRID:CVCL_0063), COS7 were obtained from ATCC (Cat#CRL-1651; RRID:CVCL_0224) and MRC-5 cells were obtained from KCLB (Cat#10171; RRID:CVCL_0440).

Primary rat (Sprague Dawley, E18) hippocampal cultures were prepared as previously described^64^. Mouse hippocampi were dissected and prepared as described in ref. ^64^ and neurons were plated at a density of 35,000 cells/18 mm-coverslip and maintained as described. For live imaging experiments, primary hippocampal cultures were transfected at 10–14 DIV using Lipofectamine 2000 (Thermo Fisher Scientific) according to the manufacturer’s instructions and imaged 48 h after transfection. For knockdown experiments, neurons were transfected at 8 DIV and imaged 96 h after transfection.

Immunocytochemistry was performed as described in ref. ^64^. Sequential labeling steps were included to avoid cross-labeling. For detection of SIPA1L2, a heat-based antigen retrieval protocol was used. This included the immersion of the cells into a 10 μM sodium citrate solution (pH = 9) for 30 min at 80 °C before proceeding with the standard protocol. The pH of the sodium citrate (Fluka) solution was adjusted with 5 M NaOH.

For immunostaining of cryosections, brains of adult *sipa1l2* wt and ko mice were post-fixed in 4% PFA and 0.5 M sucrose for 24 h and 40-μm-thick cryosections were cut.

For Nissl staining, sections were acidified with 0.05 M acetate buffer (pH 4) for 5 min and incubated with 0.05% cresyl violet acetate solution for 10 min. Sections were further incubated with 0.05 M acetate buffer (pH 4) for 3 min and dehydrated by ethanol steps. For the volumetric analysis of the DG, serial sections were collected throughout the region. Timm staining was performed as described^65^.

### EM analysis

For ultrastructural analysis, ultrathin sections were prepared from six adult male animals as described in ref. ^66^. Quantification was performed as described in ref. ^66^.

### Imaging of primary cultures and kymograph preparation

Rat primary neurons overexpressing fl-SIPA1L2-mCherry together with TrkB-GFP, GFP-LC3b or GFP-Snapin were placed in a Ludin chamber (Life Imaging Services) and imaging was performed 24–48 h later at 37 °C 5%CO_2_ on a VisiScope TIRF/FRAP imaging system from Visitron Systems based on Nikon Ti-E. The system is equipped with the Perfect Focus System (Nikon), Nikon CFI Apo TIRF 100×, 1.49N.A. oil objective, a back focal TIRF scanner for suppression of interference fringes (iLas2, Roper Scientific), and controlled with VisiView software (Visitron Systems). 488 and 561 nm laser lanes were used to excite the respective fluorophores whose fluorescence was collected through ET 488/561 Laser Quad Band filters to a The ORCA-Flash 4.0 LT sCMOS camera. Time-lapse imaging of neuronal axons was recorded at 0.5 Hz for 4 min in neuronal conditioned media. For testing the coclustering of SIPA1L2 with TrkB, SIPA1L2-mCherry or SIPA1L2-∆14-mCherry were coexpressed with TrkB-GFP in MRC5 cells and cells were imaged 24 h later in growing media for 1 min at 2 Hz. For recruitment assay of RapGAP-SIPA1L2 and Snapin, COS7 cells were cotransfected with RapGAP-PDZ-SIPA1L2-tRFP or a tRFP empty vector as control and GFP-Snapin. Coverslips were mounted on an imaging ring 24 h after transfections and cells were imaged in DMEM-based growing media. For cotrafficking of SIPA1L2 and Snapin, fl-SIPA1L2-mCherry and GFP-Snapin were overexpressed in COS7 cells for 24 h and imaged in growing media for 2.5 min at 1 Hz. Imaging of MRC5 and COS7 cells was done in the same VisiScope imaging system described above.

The analysis was performed using the free software Fiji from ImageJ (http://imagej.nih.gov/ij/) and kymographs were created using KymographClear^67^ by drawing a line along axons and Fourier-filtered kymographs generated by the plugging were displayed in figures. Retrograde and anterograde trajectories were identified by the respective location of the cell soma or axonal tip during imaging. Stationary trajectories were considered as those moving <3 μm. Axons from three independent experiments were analyzed.

In experiments where Snapin was knocked-down, imaging was performed 96 h after transfection on a Leica TCS SP5 system controlled by Leica LAS AF software using HCX PL APO 63×1.40. Areas of ~60 × 15 µM (512 × 128 pixels) were scanned using 488, 561 and 633 nm laser lanes, at 37 °C and 5%CO_2_. SNAP-Cell 647 SiR (0.5 µM) was added 1 h before starting imaging in order to visualize TrkB-SNAP.

Imaged axons were selected according to the following morphological criteria: (i) long thin processes growing far from the somatic region with constant width; (ii) without recurrent bifurcations and (iii) lacking protrusions or spines.

### Live imaging of FM_4−64_ unloading

Primary hippocampal cultures (14–16 DIV) overexpressing TrkB-GFP or GFP-LC3b were placed into a field stimulation chamber (Warner Instruments) 24–48h after transfection. The field stimulation chamber contains two platinum wire electrodes placed 10 mm apart. Cells were incubated with 10 µM FM_4–64_ (Thermo Fisher) in extracellular imaging buffer (EIB: 119 mM NaCl; 2.5 mM KCl; 2 mM CaCl_2_; 2 mM MgCl_2_; 30 mM Glucose; 25 mM HEPES in H_2_O at pH 7.4) containing 10 µM AP-5 and 4 µM CNQX^68^. Dye loading was achieved by delivering pulses for 30 s@20 Hz. Unbound dye was washed-out with EIB containing 1 mM ADVASEP-7 (Sigma-Aldrich) for 1 min followed by two washes with EIB at 37 °C. After 10 min, dye unloading was triggered by a train of 900 pulses @10 Hz in the presence of AP-5 (50 µM) and CNQX (10 µM) and dye unloading was imaged at a frequency of 1 Hz for 110 s^68^. Ten frames were acquired as a baseline before stimulation. Trafficking of either TrkB-GFP or GFP-LC3b was recorded immediately after the stimulation for an additional 10 min @1 Hz. After that, a second dye unloading protocol was delivered and imaged as described.

Imaging was performed at 37 °C 5%CO_2_ in the VisiScope TIRF/FRAP imaging system from Visitron Systems based on Nikon Ti-E described above. Laser lines of 488 and 633 nm were used to activate the respective fluorophores whose fluorescence was collected ET 488/640 Laser Quad Band filters to The ORCA-Flash 4.0 LT sCMOS camera. Pulses were evoked by delivering currents of 60 mA for 1 ms using an isolator unit controlled by a pulse generator (Master-8). Boutons were considered as visited when trajectories overlapped with the signal from the FM dye for >3 vertical pixels in the kymograph while the term nonvisited was used for those boutons within the same axon where the overlapping signals were lasting <3 pixels. Circular ROIs were placed in both types of boutons using Times Series Analyzer V3 from Fiji and mean gray values per ROI per frame were calculated from both streams of images acquired during unloading protocols. Data from each ROI were fitted to a one-phase decay from which tau was calculated using GraphPad Prism.

### Amphisomal trafficking to presynaptic boutons

Primary hippocampal neurons 14−16 DIV overexpressing tRFP-LC3b and either fl-SIPA1L2-GFP or SIPA1L2-N705A-GFP were incubated in growing media in the presence of an antibody anti-Synaptotagmin1 coupled to Oyster 660 (1:250) for 1–2 h at 37 °C 5%CO_2_. Imaging was performed for 5 min at 1 Hz at 37 °C 5% CO_2_ on a Leica TCS SP5 system controlled by Leica LAS AF software using HCX PL APO 63×1.40. Areas of ~60 × 15 µM (512 × 128 pixels) were scanned using 488, 561 and 633 nm laser lanes. Fluorescence was collected using three HyD detectors.

For experiments cLTP induction was required, neurons in growing media were treated with Rolipram (0.1 µM) and Forskolin (50 µM) for 10 min and cells were imaged after media replacement with EIB . When needed, H89 (10 µM) was added for 30 min before proceeding with the cLTP induction.

Dwelling time of the amphisome in boutons was calculated from kymographs by drawing a vertical line using Fiji in cotrajectories that overlapped with the synaptic marker and the number of pixels was quantified. Only those stopovers where the overlap occurs for >3 vertical pixels were considered. Instant velocity was calculated from the angle of each cotrajectory obtained by drawing a line along each trajectory. Run-length was also assessed by drawing a line and measuring its dimensions using Fiji. Frequency distributions were generated in GraphPad Prism.

### Imaging and quantification of pSyn/Syn ratio

Hippocampal primary cultures from wt and *sipa1l2*^*−/−*^ mice were transfected at 10 DIV with either fl-SIPA1L2-mCherry, SIPA1L2-∆14-mCherry or empty vector for control. Transfected neurons were treated at 14 DIV with TrkB-Fc (500 ng) overnight or with BDNF (100 ng) for 30 min.

Immunocytochemistry was performed with anti-Synapsin I,II, anti-phospho Synapsin (Ser62) and anti-mCherry primary antibodies. Stack images were acquired in a Leica TCS SP5 system controlled by Leica LAS AF software using HCX PL APO 63 × 1.40. Areas of 82 × 82 µM were scanned with 488, 568 and 635 nm laser lanes (12-bits, 80 × 80 nm pixel size, 700 Hz, Z-step 0.25 µm). Fluorescence was collected using three HyD detectors.

Circular regions of interest (ROIs) were placed using Time Series Analyzer v3 on the Synapsin signal and pSyn/Syn ratio was calculated for single boutons from the mean gray intensity obtained in Fiji for each ROI. Boutons from the same axon were averaged and average from axons is plotted on the graph. For each rescue group, data were normalized to the group of neurons treated with TrkB-Fc.

### Imaging of Sy-EKAR

Neurons overexpressing Sy-EKAR and either with SNAP-SIPA1L2 or SNAP-LC3b were imaged in EIB. The far-red SNAP-Cell 647 SiR substrate at a concentration of 0.5 µM was added to the growing media for 1 h and was washed out (3×) with EIB.

FLIM measurements were carried out at 37 °C and 5%CO_2_ using an Abberior multichannel confocal/STED microscope based on a Nikon Ti-E microscope body with perfect focus system. For excitation three pulsed lasers at i.e. 470, 561, 640 nm were employed. A ×60 P-Apo oil immersion objective (NA 1.4) was used. In the detection path, the emitted light was directed through a pinhole set to match 1 Airy at 540 nm. Three avalanche photodiodes APDs were used for recording the fluorescence signal in the green 470−520 nm, red 595−635 nm and far-red 615–755 nm range. For measuring the fluorescence lifetime a Becker&Hickl SPC150 TCSPC board was used after setting the APDs in single-photon count mode (spcm).

A small area of interest containing 2–5 boutons was chosen. Two hundred FLIM images were recorded in streaming mode detecting only the green and the far-red channel. In this way, it was possible to record 2–4 FLIM images/s (pixel size was set to be 110 nm). The acquisition speed of any FLIM time-lapse experiment carried out in this work can be calculated using:

1 $${{V}} = 16{{vN}}_{{\mathrm{p}x}}{{N}}_{{\mathrm{p}y}},$$

where *V* is the acquisition speed in frames/s, 16 is the total number of line accumulations used for imaging the green and far-red channel, *v* is the pixel dwell time set to be 3 µs in all the experiments and ${{N}}_{{\mathrm{p}x}}/{{y}}$ is the *x* and *y* axis image pixel size. FLIM measurements were carried out in sampling the acquisition period of 19 ns in 50 steps of 0.38 ns.

FLIM images were analyzed by a self-made Matlab script where boutons were automatically localized by finding the local peaks of the intensity signal in the sum projection images and 5 × 5 pixels-ROIs were placed. The time series of the mean fluorescence lifetime was produced from each ROI by averaging the pixel by pixel measured lifetime. The lifetime information was extracted with the help of a least square algorithm. A mono-exponential decay function was used as a model function in the least square algorithm.

Boutons in axons without any detected trafficking were used as a control. Mean lifetime calculated from each bouton before - 4 frames -,during and after the visit of the amphisome and ∆Lifetime was calculated for each bouton. Lifetime_0_ was considered as the mean lifetime in the frame before the arrival of the amphisome.

### Confocal and STED imaging

Gated STED images were acquired with a Leica TCS SP8 STED 3X equipped with pulsed White Light Laser (WLL) and diode 405 nm laser for excitation and pulsed depletion with a 775 nm laser. A Leica HC APO CS2 ×100/1.40 oil objective was used. Images were taken as a single plane of 1024 × 1024 pixels and optical zoom ranging from 5 to 6, at 400 lines per seconds and 4× line averaging. Pixel size was 20–23 nm in *xy*. Time gates were set to 0.5 to 6 ns. Raw STED and confocal images were deconvoluted using Deconvolution wizard (Huygens Professional, SVI) and a theoretical point spread function (PSF) based on optical microscopic parameters until it reached a Quality threshold of 0.05.

Mander’s coefficient was calculated on deconvoluted images using either colocalization analyzer plug-in (Imaris, Bitplane) or the Colocalization test plugin from ImageJ. Briefly, the region of interests (ROIs) for presynaptic boutons were defined by intensity threshold of Bassoon staining. Manders M1 coefficient (SIPA1L2/ pTrkB^Y515^) with Costes thresholding was measured within the synaptic boutons as a fraction of the total fluorescence found in common presynaptic ROIs.

### Coimmunoprecipitation

Coimmunoprecipitation experiments were performed as described before^62^.

Briefly, ~500 mg of rat hippocampal tissue was homogenized in 2.5 ml/g in the following buffer: 10 mM HEPES, 320 mM sucrose, 1 mM EDTA, pH 7.4, PhosSTOP phosphatase inhibitors and complete proteases inhibitors (Roche Holding AG, Basel, Switzerland) by applying eight strokes at 900 rpm in a Teflon pistol homogenizer and subsequently centrifuging the samples for 10 min @800 × *g*. This procedure was repeated three times and the resulting supernatants were collected and centrifuged for 10 min at 3500 × *g*. The pellet was resuspended in 2.7 ml/g lysis buffer (150 mM NaCl, 50 mM TRIS, 0.5% DOC, 1% Triton X-100, PhosSTOP phosphatase inhibitors and complete proteases inhibitors) and incubated @4 °C with rotation. Samples were then centrifuged for 15 min @20,000 × *g*, and the supernatant was used for immunoprecipitation. An amount of 700 μl of tissue extract was incubated with 4 μl of goat anti-TrkB (R&D, 200 μg/μl) and 2 μl of goat IgG (sc-2028, 500 μg/μl), as well as 5 μl of rabbit anti-SIPA1L2 and 5 μl of rabbit IgG (sc-2027, 400 μg/μl) overnight @4 °C with rotation. Forty microliters of magnetic Protein G Dynabeads was added into the mixture for an additional 3 h for IgG coupling. The unbound fraction was removed and beads were washed with the extraction buffer. Proteins were eluted and subjected to SDS-PAGE.

For immunoprecipitation with DIC complex, 5 µg of anti-DIC antibodies or IgG mouse control antibodies were coupled to magnetic Protein G Dynabeads (Life Sci. Tech.) for 16 h. Subsequently, beads were washed three times in PBS pH 7.4 containing 0.1% (w/v) BSA and resuspended in 200 µl binding buffer (PBS, pH 7.4, containing 2 mM EDTA and 0.1% (w/v) BSA. For coimmunoprecipitation, 120 µl of the crude light membrane fraction was added and incubated for 16 h with constant rotating. All steps were performed at 4 °C. After substantial washing, proteins were eluted from the beads in 60 µl SDS sample buffer, boiled at 95 °C, subjected to 8% SDS-PAGE and analyzed by WB. Representative blots are shown uncropped and unprocessed in the Source Data file.

### Lipid raft isolation

Cerebella were homogenized in lysis buffer (50 mM Hepes pH 7.4, 100 mM NaCl, 3% Brij 58, 5 mM EDTA, 10 mM Na_4_P_2_O_7_, protease inhibitors). The lysate was mixed with 1.5 ml ice-cold sucrose (final concentration: 40%) and transferred to a Potter S (B.Braun, Germany). A homogenization step was performed and 3 ml of homogenate were transferred into a centrifuge tube. The sample was overlaid with 6 ml ice-cold 30% sucrose and then again overlaid with 3 ml ice-cold 5% sucrose and subjected to centrifugation for 20 h at 200,000 × g @4 °C. Twelve samples of 1 ml obtained and proteins of each sample were precipitated with acetone. Fraction 13 (pellet) was diluted in lysis buffer. Pellets from all fractions were lyophilized and resuspended in SDS protein sample buffer for SDS-PAGE and immunoblotting.

### Heterologous coimmunoprecipitation

HEK-293T cells growing in T75 Flasks (Thermo Fisher Scientific) were transfected, harvested and lysed in 1 ml of RIPA buffer (50 mM Tris-HCl pH 7.4, 150 mM NaCl and 1% Triton X-100, 0.5% sodium deoxycholate and 0.1% sodium dodecyl sulfate (SDS)) for 1 h@4 °C. The lysate was cleared by centrifugation incubated with anti-GFP/myc-antibody-coated magnetic beads (MultiMACS GFP Isolation Kit #130-094-253 or Miltenyi Biotec GmbH, Germany). The coimmunoprecipitation was done following the manufacturer’s protocol (Mitenyibiotec, Bergisch-Gladbach, Germany).

For coimmunoprecipitation of fl-SIPA1L2-mCherry and SIPA1L2-Δ14-mCherry with TrkB-GFP, the following lysis buffer was used: 50 mM TRIS, 150 NaCl, 1% NP-40, 0.1% SDS, protease inhibitors (Complete, Roche), PhosphoStop (Roche). For heterologous coimmunoprecipitation of RapGAP and PDZ domains of SIPA1L2 with GFP-Snapin as well as coimmunoprecipitation of endogenous DIC and fl-SIPA1L2-mCherry with either GFP-Snapin-S50A or GFP-Snapin-S50D, the lysis buffer was without SDS. Representative blots are shown uncropped and unprocessed in the Source Data file.

### Pull-down assays

Proteins were bacterially purified as previously described^62^.

For interaction assays between SIPA1L2 and TrkB, the matrix (amylose-MBP) alone or coupled with different fragments of the TrkB receptor was washed three times and resuspended in pull-down buffer (20 mM Tris pH 7.5, 1 mM DTT, 3 mM EDTA, 100 mM NaCl, 0.3% TritonX-100) and protease inhibitors (Complete, Roche). Subsequently, amylose resin was incubated with 2 μg of a GST-ActI-SIPA1L2-1-624 or GST-ActII-SIPA1L2-1026-1650 overnight @4 °C. Reciprocally, 10 µg of ether GST-SIPA1l2-86-99 or GST alone as a control was immobilized on Glutathione Sepharose resin equilibrated with PBS buffer (140 mM NaCl, 2.7 mM KCl, 10 mM Na_2_HPO_4_, 1.8 mM KH_2_PO_4_, pH 7.3, protease inhibitor (Complete, Roche)), incubated with 20 μg of purified MBP-TrkB-454-465 for 120 min@4 °C in PBS buffer and washed three times with PBST buffer (PBS, protease inhibitor, 0.1% Triton X-100). All samples were eluted with 2× SDS loading buffer and an equal amount of samples were used for SDS-PAGE.

For experiments with the TAT peptides, MBP-TrkB-454-821 was coupled to amylose resin (NE BioLabs) and incubated in the presence of 10× molar access of either TAT-SIPA1L2 or TAT-scr peptides with 1 μg of GST-SIPA1L2-1-624 fusion protein @4 °C overnight. The resin was washed three times with 1 ml of pull-down buffer (see buffer composition above) and boiled with 50 μl of 2× SDS sample buffer for 5 min before SDS-PAGE. Blots were then analyzed by immunodetection with anti-SIPA1L2 antibodies. For interaction between SIPA1L2 and LC3, 5 µg of Intein-RapGAP-SIPA1L2 (470–838) and Intein-Caldendrin were immobilized on chitin resin (NE BioLabs). The resin was washed with TBS buffer (150 mM NaCl, 20 mM Tris-Cl, pH 7.4, 5 mM MgCl_2_, EDTA-free protease inhibitors (Complete, Roche)) and incubated with purified 6xHis-tag LC3b for 120 min @4 °C in TBS buffer either alone or in the presence of purified 6xHis-Snapin. Samples were then washed with TBS-T buffer (TBS + 0.1% Triton X-100), eluted with 2× SDS sample buffer, subjected to SDS-PAGE and immunoblotting. For pull-down assay of SIPA1L2 with endogenous LC3, whole brain extract was incubated with GST-SIPA1L2-RapGAP, GST-SIPA1L2-PDZ as well as with GST alone. Immunodetection was done with anti-LC3b antibodies. Representative blots are shown uncropped and unprocessed in the Source Data file.

### Rap1-GAP activity assay

RapGAP activities were assessed using Active Rap1 detection kit (Cell Signaling)^69^ following the manufacturer’s instructions. Briefly, the assay employs Rap1-binding domain (RBD) of RalGDS as an activation-specific probe for Rap1. Fused to GST-RalGDS-RBD allows to pull down the amount of nonhydrolized Rap1 in the presence of RapGAP. For experiments involving overexpression of fl-SIPA1L2, RapGAP and LIR-SIPA1L2 mutants GST-RalGDS pull down of GTP-bound Rap1 was performed from HEK293T cell extracts. For experiments involving LC3b and PKA phosphorylation of SIPA1L2-S990, Intein-tagged SIPA1L2-470-838, SIPA1L2-470-1025, SIPA1L2-470-1025-S990D, 6xHis-tagged Rap1b as well as 6xHis-targged LC3b were bacterially purified. Purified Rap1b was incubated with 50 mM GTP in 200 μl of loading buffer (50 mM TRIS, 100 mM NaCl, 2 mM EDTA, 1 mM DTT) for 20 min at 37 °C for loading recombinant Rap1b with GTP. The loading reaction was terminated by adding 10 mM MgCl_2_. Subsequently, RapGAP assay was carried out in the presence of SIPA1L2-RapGAP domain (470–838 aa, ~20 μg), RapGAP-PDZ (470–1025, ~30 μg) and RapGAP-PDZ (470-1025-S990D, ~30 μg) of SIPA1L2 in the presence of either His-tagged LC3b (~50 μg) or His-SUMO (~50 μg) as a control for 15 min at 30 °C on shaker (650 rpm). Representative blots are shown uncropped and unprocessed in the Source Data file.

### Preparation of autophagosomal-enriched fraction

A whole brain homogenate from two rats was subjected to density gradient separation and purification as already described with minor modifications^70^. Briefly, rat brains were homogenized and subject to differential centrifugation through Nycodenz gradient (15, 20, 24, 26, 85%) for 3 h@105 × *g*. Each fraction (A1—15–20%, A2—20–24% and L—24–26% interfaces) was collected, diluted with Percoll and centrifuged for 1 h@105 × *g* to obtain a virtually pure pellet and analyzed using antibodies against SIPA1L2, TrkB, Snapin, LC3b, and Rab7.

### Statistical analysis

Data in the manuscript are shown as mean ± SEM and *n* numbers used for statistics are depicted in each panel or figure legends from at least three independent experiments. Graphs and statistical analysis was made with GraphPad Prism (GraphPad Software). Statistical tests used are given in the figure legend of the corresponding experiment. Briefly, D’Agostino−Pearson omnibus normality tests were performed to assess the normality of samples. Subsequently, parametric and nonparametric tests were chosen accordingly. For comparison between normally distributed samples, two-tailed unpaired Student’s *t* test was used for comparing two groups and one-way ANOVA with Bonferroni’s posthoc test was used for comparison between more than two groups. For not normally distributed samples, Mann-Whitney *U* test or Kolmogorov-Smirnov test were used to compare two groups and Kruskal-Wallis with Dunn’s correction was used for comparing not normally distributed samples. *P* values < 0.05 were considered significant.

### Reporting summary

Further information on research design is available in the Nature Research Reporting Summary linked to this article.

## Supplementary information

Supplementary Information

Peer Review

Reporting Summary

Description of Additional Supplementary Files

Supplementary Movie 1

Supplementary Movie 2

## Data Availability

All data supporting the findings described in this study are available from the corresponding author upon reasonable request. Source data from all representative blots shown in this study as well as data underlying all quantitative analysis performed in this study are provided with the manuscript as a Source Data file.

## Source data

Source data
