# Supplementary material for: SIPA1L2 controls trafficking and local signaling of TrkB-containing amphisomes at presynaptic terminals
Source: Nat Commun. 2019 Nov 29;10:5448. doi: 10.1038/s41467-019-13224-z (PMC6884526; doi:10.1038/s41467-019-13224-z)
Supplement: Supplementary file 1 — Supplementary Information [file 41467_2019_13224_MOESM1_ESM.pdf]

Supplementary Information

SIPA1L2 controls trafficking and local signaling of TrkB-containing amphisomes at presynaptic terminals

Andres-Alonso et al.

Supplementary Figures

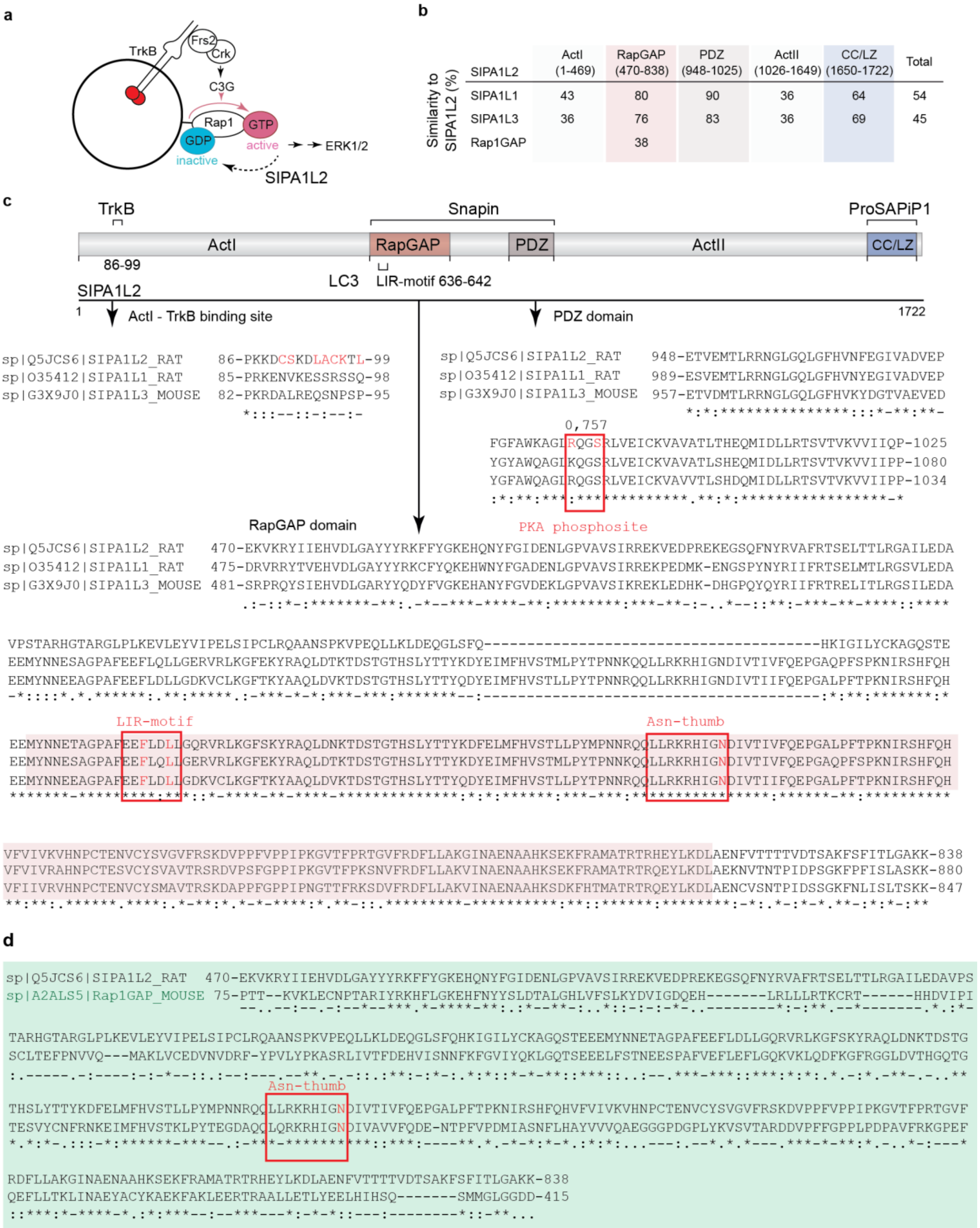

Supplementary Figure 1. SIPA1L2 shows high degree of similarity with other SIPA1L family members and Rap1GAP.

a. Cartoon depicting the molecular cascade leading to the TrkB-dependent ERK1/2 activation. Upon BDNF binding, sustained ERK1/2 is achieved after TrkB activation by recruitment of the protein adaptor fibroblast growth factor receptor substrate-2 (Frs2) that provides the binding for Crk. This association promotes the activation of C3G, the guanyl nucleotide exchange factor (GEF) for Rap1 that triggers the conversion of the inactive, GDP-bound to the active GTP-bound Rap1. Rap1-GTP stimulates B-Raf and activates the ERK cascade (see [7] for a

detailed revision). SIPA1L2 is a RapGAP for Rap1/2 that stimulates the GTPase activity of Rap and promotes the inactivation of the pathway.

b. Table showing the similarity of SIPA1L2 with SIPA1L1 and SIPA1L3 as well as the RapGAP domain of Rap1GAP. Comparisons were made according to the SIPA1L2 domains defined by the amino acids depicted in the heading. The RapGAP and PDZ domains are highly conserved among SIPA1L family members.

c. Scheme showing the domain organization and interaction motifs of SIPA1L2 with TrkB, Snapin, LC3 and ProSAPiP1 [9]. Depicted is the sequence comparison of the ActI, RapGAP and PDZ domains between SIPA1L family members. Boxes within the RapGAP domain indicate the LIR-motif and the Asparagine thumb where amino acids in the so-called LIR-mutant (F638A-L641A) and RapGAP dead mutant (N705A) are indicated in red. Unless otherwise stated, interaction assays performed in this study involving the RapGAP or PDZ domain were done with SIPA1L2-624-813 (SIPA1L2-RapGAP; red shaded box in the figure) and SIPA1L2-948-1025 (SIPA1L2-PDZ). The alignment for the RapGAP domain is done with a longer sequence stretch (470-838) defined by comparison with a published, catalytically-active fragment of Rap1GAP (aa 75-415; 23). The fragment SIPA1L2-470-838 was used in RapGAP activity assays. Symbols represent: fully conserved residues (*), groups with strongly similar properties (:) and groups with weakly similar properties (.) according to ClustalW2.

d. Sequence comparison of SIPA1L2 with the catalytically active RapGAP domain of Rap1GAP. Symbols represent: fully conserved residues (*), aa with strong similarity (:), aa with no similarity (.) according to ClustalW2.

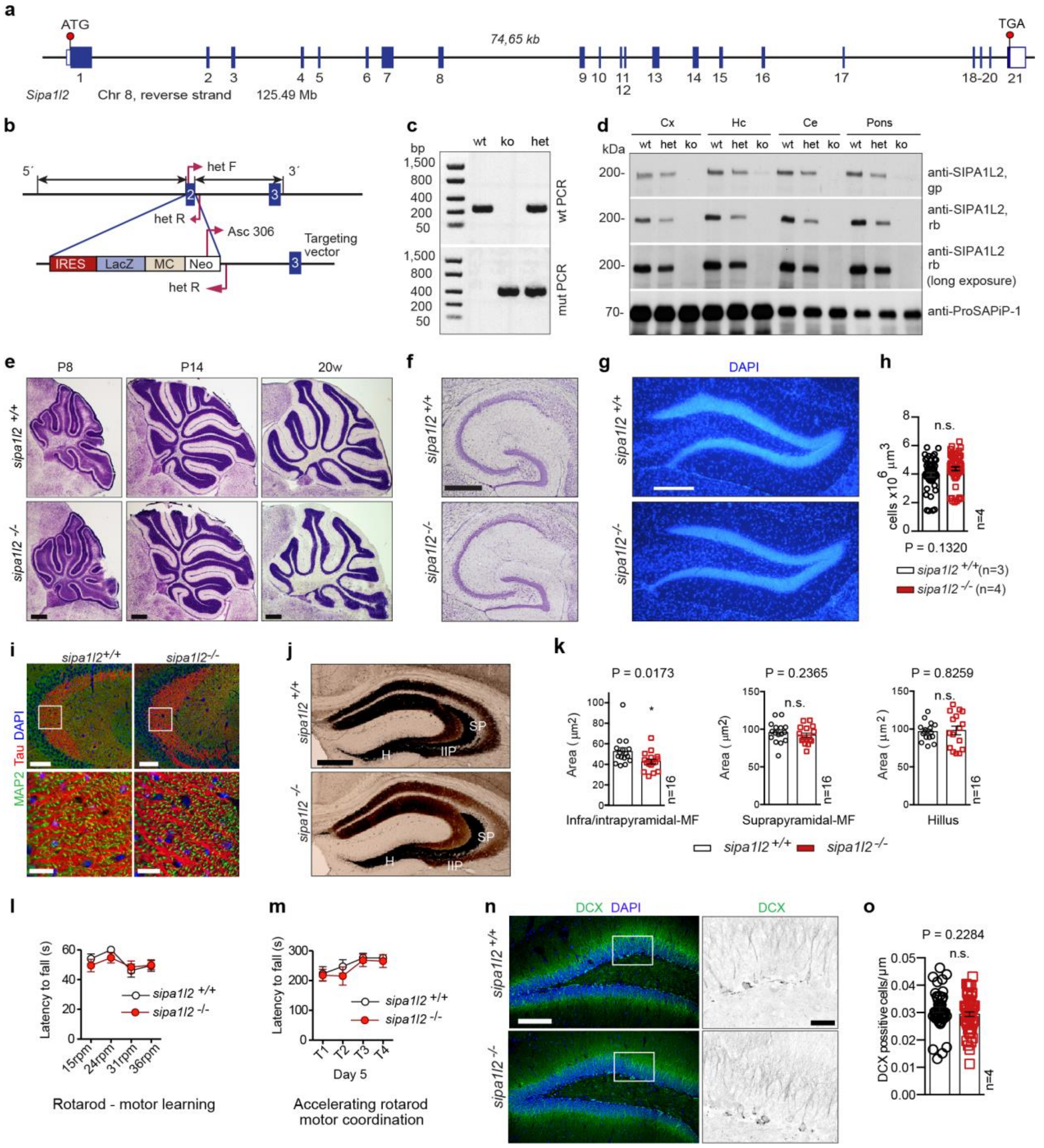

Supplementary Figure 2. Generation and characterization of *sipa1l2* knockout mice.

a. Schematic representation of the *sipa1l2* genomic structure comprising 21 exons located on mouse chromosome 8 with one alternatively spliced exon (no. 18).

b. Scheme depicting the strategy used for generation of the constitutive *sipa1l2* knockout mice. The targeting vector replaces exon 2 with an IRES-LacZ sequence. The positions of primers for genotyping analysis (hefF, hetR and Asc306) are indicated by pink arrows.

c. Representative gel of mouse genotyping by PCR with primers indicated in **b**.

d. Western blot on mouse brain tissue from different areas of *wt*, *sipa1l2 het* and *ko* mice (Cx, Cortex; Hc, hippocampus; Ce, cerebellum; Pons). Specific SIPA1L2 antibodies from different species (guinea pig and rabbit) were used. The expression of ProSAPiP1 was not altered.

e. Nissl-stained saggital cerebellar sections from *ko* and *wt* mice at different developmental stages. Scale bar = 500 μm.

f. Nissl staining from mouse *wt* and *ko* hippocampal sections (scale bar = 500 μm).

g-h. Representative DG images (**g**) and quantification (**h**) from wt and ko slices stained with DAPI. Scale bar =150 μm.

i-k. Tau (**i**) and Timm staining (**j**) performed in *sipa1l2* ko and wt slices. In (**j**) H stands for Hilus, SP for suprapyramidal MF and IIP for infra-and intrapyramidal MF (Mann Whitney U test for infra/intrapyramidal and suprapyramidal regions and Student´s t test for quantification in the Hillus). Scale bar =100 μm and 20 μm (inset). Scale bar for Timm staining is 400 μm.

l-m. In **l,** quantification of the latency to fall of *ko* and *wt* mice submitted to 4 days of rotarod task with increased speeds each day (maximum latency of 60 sec). In **m**, animals were submitted on day 5 to the accelerating rotarod task 4 times (maximum latency 300 sec). (Two-way ANOVA, n animals=9).

n-o. Representative images (**n**) from hippocampal sections from *wt* and *ko* mice stained with doublecortin (DCX) for adult-born granule cells and DAPI and quantification (**o**). Scale bar =100 μm and 20 μm (inset).

Data is show as mean ± S.E.M. n.s. stands for not significant.

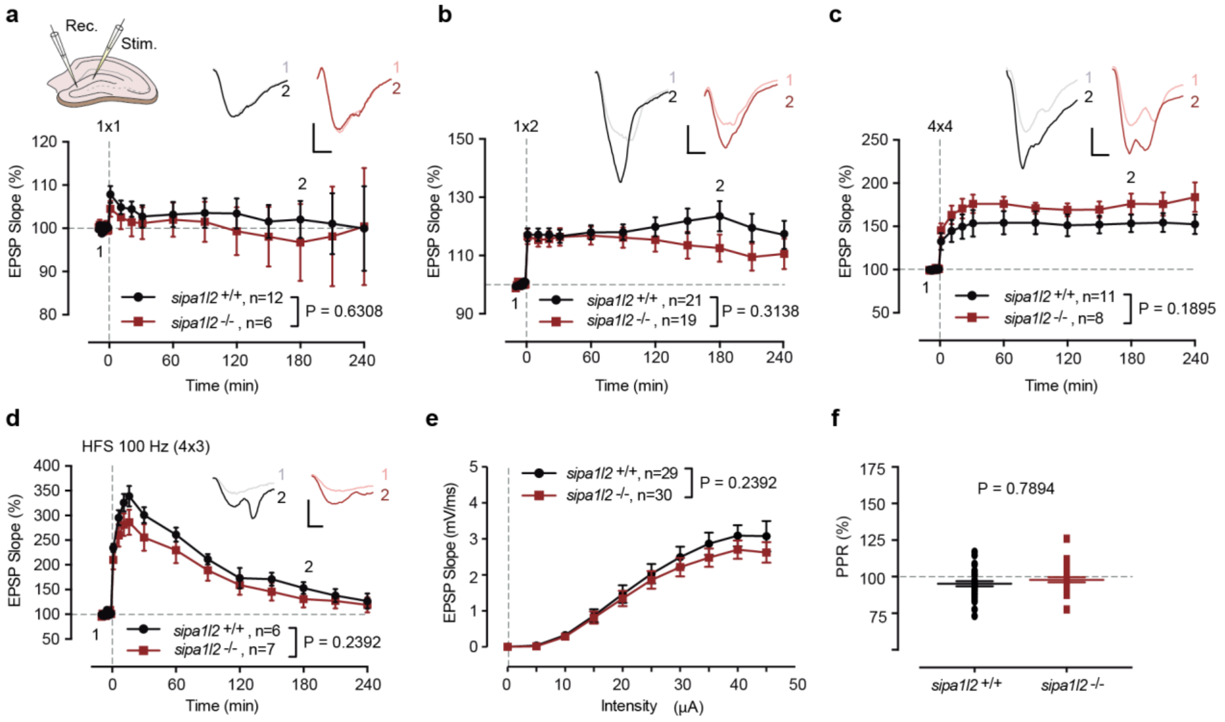

Supplementary Figure 3. Basal synaptic transmission and synaptic plasticity in the DG of *sipa1l2 ko* animals was similar to *wt* mice.

a-b. EPSP slopes after theta burst stimulation (TBS) of different strengths delivered on acute slices from *wt* and *ko* mice. Delivery of one brief presynaptic burst of 10 pulses at 10 Hz - 0.2ms per half-wave duration - repeated 10 times at 5Hz (**a**) or two of these episodes (inter-episode interval 10s) (**b**) resulted in similar responses in both experimental groups (two-way ANOVA).

c-d. EPSP slopes upon delivery of a stronger TBS protocol consisting of 4 episodes (inter-episode interval 10s) repeated 4 times every 5min (**c**) or a high-frequency stimulation protocol (4x100Hz 1s, every 5min) (**d**) (two-way ANOVA).

e-f. No differences in input-output curve of the field excitatory postsynaptic potential (**e**) and paired-pulse facilitation (**f**) between *sipa1l2*$^{+/+}$ and *sipa1l2*$^{-/-}$ were observed (Student´s t test).

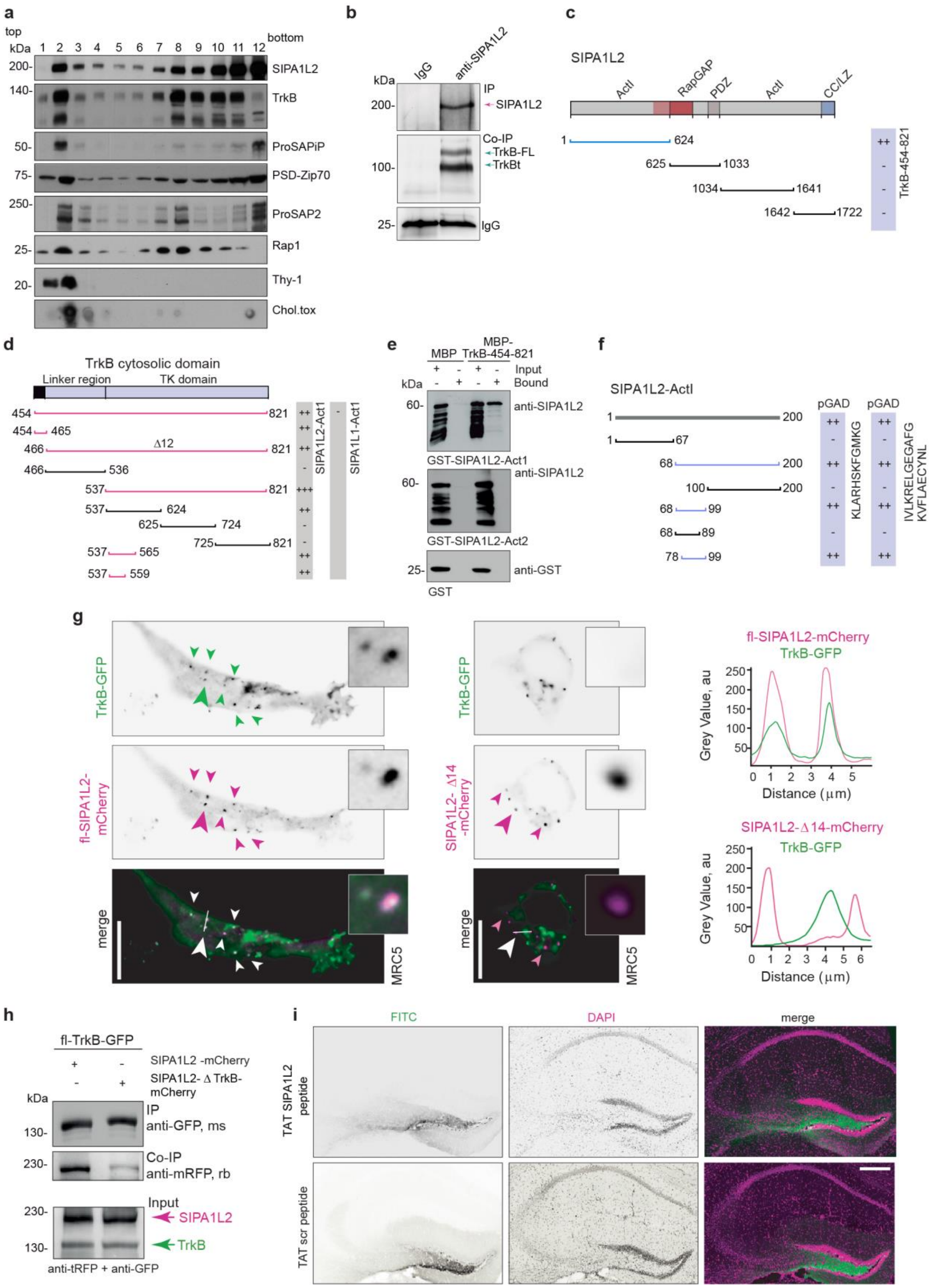

Supplementary figure 4. SIPA1L2 interacts with TrkB.

a. Lipid raft isolation from rat cerebellum shows that SIPA1L2 distribution overlaps with TrkB and Rap1 and associates with lipid rafts identified by Thy-1. Lipid raft fractions were also identified by sphingolipids-dot blot assay using Cholera toxin (lower panel).Twelve fractions (from top to bottom of the gradient) were immunoblotted for indicated proteins.

b. TrkB co-immunoprecipitates with endogenous SIPA1L2 from rat hippocampus. SIPA1L2 was immunoprecipitated with anti-SIPA1L2 rabbit antibodies (IP) and rabbit IgG was used as a control. Western blot of precipitates was detected anti-TrkB goat antibodies.

c. Schematic representation of SIPA1L2 domains and the corresponding fragments used in YTH. Results are depicted in the light-blue box on the right. Numbers indicate amino acid (aa) residue position in each protein according to the rat sequence. The intensity of the binding is represented as: "+++" <60 min, "++" 60-120 min, "+" 120-180 min, and – for no interaction

d. Scheme depicting the fragments used in YTH and corresponding interactions. Interacting fragments are shown in pink. Two binding interfaces in TrkB were found to interact with ActI-SIPA1L2: 12aa within the juxtamembranous region of TrkB (TrkB-454-465) and the first 23aa the tyrosine kinase (TK) domain (TrkB-538-560).

e. MBP-pull-down assays confirmed the direct interaction of the ActI domain in SIPA1L2 with the cytosolic part of TrkB (MBP-TrkB-454-821). A band at about 60kDa is visualized in the WB using anti-SIPA1L2 (rb) antibodies. The ActII domain of SIPA1L2 (68 kDa; GST-SIPA1L2-1026-1650) as well as GST alone does not interact with the cytosolic TrkB.

f. Identification of the TrkB-binding interface within the Act1 domain of SIPA1L2 by YTH. The first 200 aa of the ActI domain were chosen as bait and the interaction with the juxtamembranous region of TrkB (aa 454-465) as well as with the first 23 aa of TK domain (TrkB-537-559) was verified.

g. Representative images and line profiles showing the co-localization between TrkB-GFP with SIPA1L2-mCherry, but not SIPA1L2-Δ14-mCherry

h. Heterologous co-immunoprecipitation experiments between GFP-SIPA1L2-Δ86-99 or fl-SIPA1L2 and TrkB.

i. Representative images show coronal brain sections from injected mice after behavioral testing. Scale bar is 200 µm.

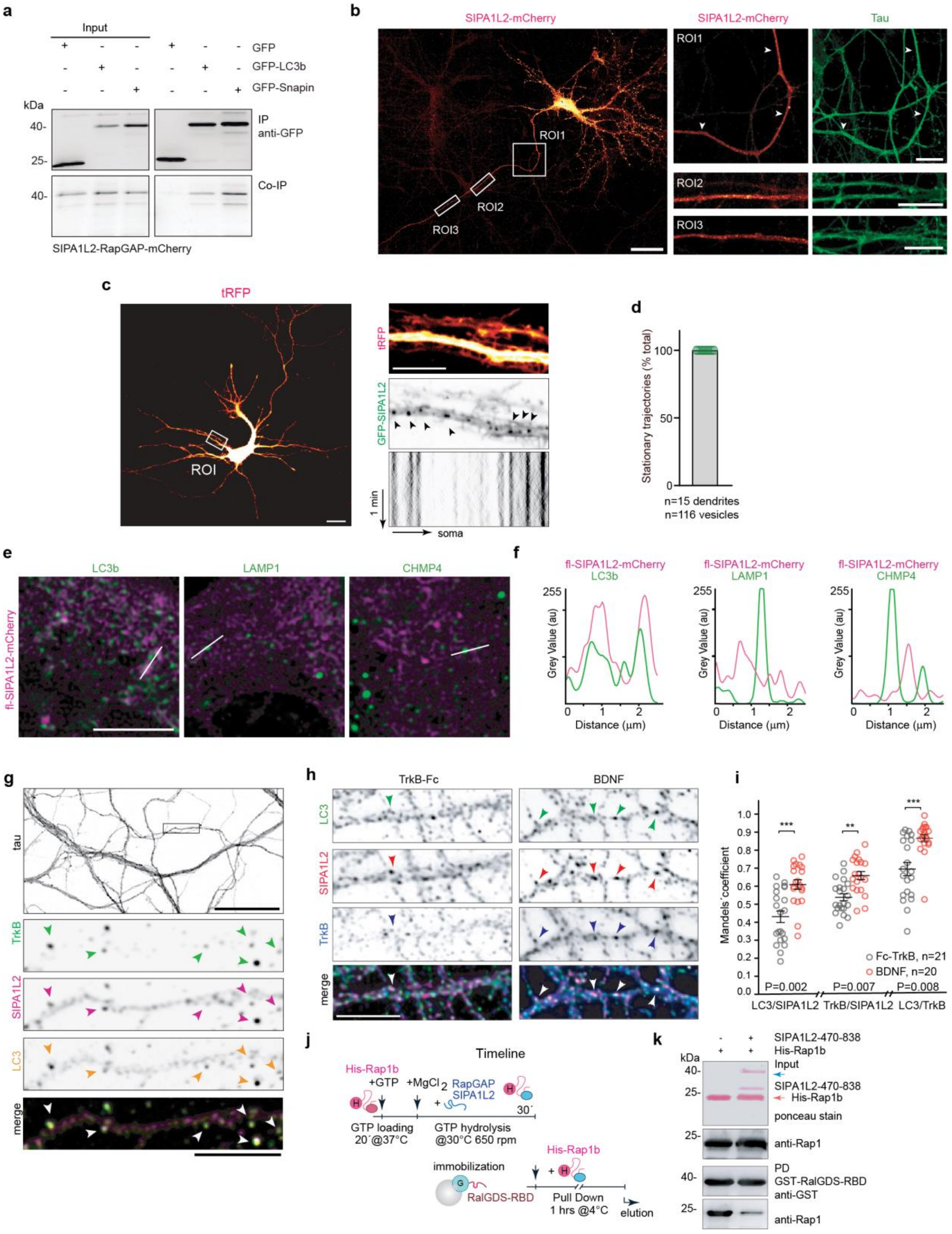

Supplementary figure 5. SIPA1L2 associates with Snapin, TrkB and LC3b, but not with LAMP1 and CHMP4.

a. RapGAP domain of SIPA1L2 (aa 624-813) co-immunoprecipitates with both GFP-Snapin as well as with GFP-LC3 from HEK293T cell extracts.

b. Confocal tile scanned image of neuron overexpressing fl-SIPA1L2-mCherry co-stained with Tau showing representative distal axonal regions used for co-trafficking assays. Scale bars are 50 µm and 10 µm within the ROIs.

c-d. Confocal images showing a neuron (DIV 10) overexpressing tRFP and GFP-SIPA1L2. The depicted ROI shows the dendritic region live-imaged for 5 minutes to assess the dendritic trafficking of SIPA1L2. Kymograph shows stationary particles and the bar graph (**d**) shows the percentage of stationary particles quantified per dendritic segment. Scale bars are 20 µm in the overview image and 10 µm in the ROI.

e-f. Representative confocal images (**e**) of MRC5 cells overexpressing fl-SIPA1L2-mCherry and stained against endogenous LC3b, LAMP1 or CHMP4 (multivesicular bodies marker) and corresponding line profile (**f**). Scale bar is 5 µm.

g. Confocal images from rat hippocampal cultures immunostained for Tau, TrkB, SIPA1L2 and LC3 showing the colocalization of the later three in axons. Scale bar = 20 µm in overview image, 5 µm in insert.

h-i. Representative confocal images (**h**) from rat primary cultures immunostained for LC3, SIPA1L2 and TrkB after treatment with TrkB-Fc bodies or BDNF and corresponding Manders´colocalization coefficient (**i**). Circles represent single analyzed images. Black bars are mean±S.E.M. (Mann-Whitney U test). Scale bar = 10 µm.

j-k. Time line (**j**) of the Rap1b loading with GTP and conditions for Rap1b-GTP hydrolysis in the RapGAP assay (**k**) after addition of SIPA1L2. SIPA1L2-(470-838) is generated based on sequence alignment with the catalytically active RapGAP-domain of Rap1GAP.

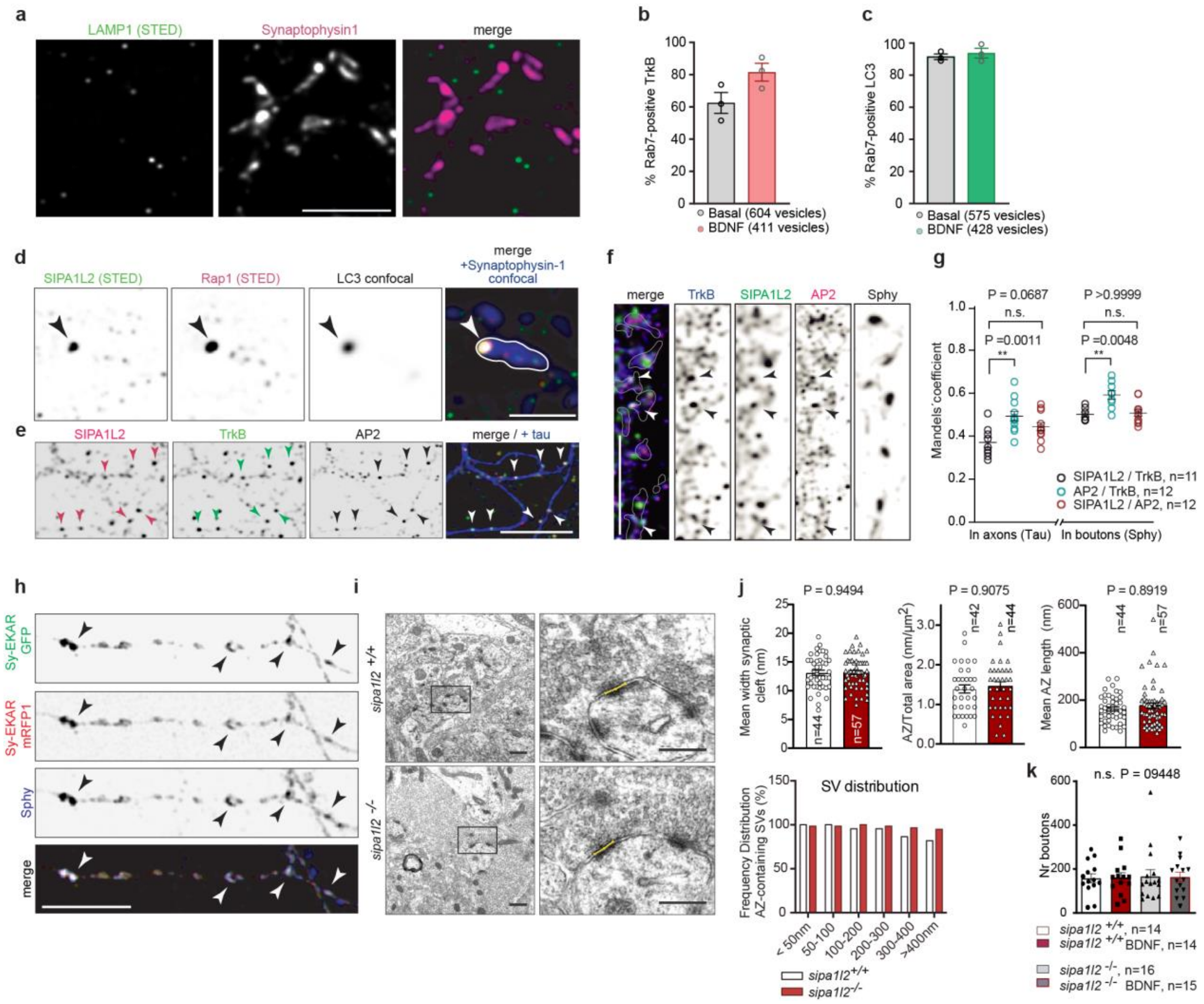

Supplementary Figure 6. SIPA1L2/TrkB/LC3 signaling amphisomes are positive for AP2 and absence of SIPA1L2 does not result in changes of MF ultrastructure.

a. Representative images of rat hippocampal neurons stained against LAMP1 and Synaptophysin1 and imaged using STED and confocal microscopy revealed no staining for LAMP1 at presynaptic boutons labeled with Synaptophysin1. Scale bar is 2,5 μm.

b-c. Quantification of the percentage of TrkB (**b**) and LC3 (**c**) organelles that are positive for Rab7 from the experiment in Figure 7a in which primary hippocampal neurons were stained against SIPA1L2, TrkB, LC3 and Rab7. Circles represent averaged data per imaged quantified. Number of vesicle in the legend represent total particles analyzed from the three images.

d. Quadruple immunofluorescence combining confocal and super-resolution STED imaging depicting association of Rap1 with SIPA1L2/LC3 positive amphisomes in the presynaptic boutons from hippocampal primary neurons. Scale bar is 1 μm

e-g. Immunostaining of primary hippocampal cultures showing the colocalization of SIPA1L2/TrkB/AP2 in axons labeled with anti-tau (**e**) and boutons (**f**) labeled with anti-Synaptophysin-1 antibodies and corresponding Manders´ co-localization coefficient (**g**). Circles in the graph represent average Manders' coefficient per image and "n" numbers of analysed images (One-way ANOVA). Scale bars are 5 μm.

h. Representative confocal images from rat hippocampal neurons transfected with Sy-EKAR (GFP-mRFP1) and immunostained for Synaptophysin-1 (Sphy-1) showing the localization of the ERK sensor in presynaptic terminals. Scale bar is 10 μm.

i-j. Representative EM images (**i**) and quantification (**j**) of mean width of the synaptic cleft, mean active zone length and frequency distribution of AZ-containing synaptic vesicles. Representative active zones are shown in yellow. "N" numbers are within columns represent the number of images analyzed. Scale bars – 500 nm (overview) and 300 nm (insert).

k. Number of boutons detected by immunofluorescence against Syn in *wt* and *sipa1l2*$^{-/-}$ neurons after treatment with BDNF as compared to control, non-treated cells. Data shown as mean±S.E.M. "n" numbers represent independent cells. n.s.=p>0.05 (One-way ANOVA with Bonferroni posthoc test).

Supplementary tables

Supplementary table 1. Description of the antibodies, constructs and oligonucleotides used in this study and corresponding sources.

Antibodies	Source	Identifier / Dilution
Rabbit anti-SIPA1L2	[1]	N/A; WB: 1:1000
Guinea pig anti-SIPA1L2	[1]	N/A; IF/STED-1:150
Goat anti-TrkB (cys32-His429)	R&D Systems	Cat#AF1494-SP; RRID:AB_2155264; IF/STED-1:200
Rabbit anti-pTrkA (Tyr490) (pTrkBY515)	Cell Signaling	Cat#9141S; RRID:AB_2298805; IF-1:200
Mouse anti-Rab7 (clone Rab7-117)	Abcam	Cat#ab50533; RRID:AB_882241; IF-1:300
Rabbit anti-Rab7	Cell Signaling Technology	Cat#2094; RRID:AB_2300652
Mouse anti-Synaptotagmin 1 (cytoplasmic tail)	Synaptic Systems	Cat#105011; RRID:AB_887832; IF-1:400
Mouse anti-Synaptotagmin 1 labeled with Oyster650	Synaptic Systems	Cat#105311C5; RRID:AB_2199308; IF-1:300
Guinea pig anti-Synaptophysin 1	Synaptic Systems	Cat#101004; RRID:AB_1210382; IF-1:400
Mouse anti-Synaptophysin 1 labeled with Oyster488	Synaptic Systems	Cat#101011C2; RRID:AB_10890165 IF-1:300
Mouse anti-LC3 (clone5F10)	NanoTools	Cat#0231-100;RRID:AB_2722733
Mouse anti-LC3 (human)	MBL	Cat#M152-3; RRID:AB_1279144; IF/STED-1:250
Rabbit anti-LC3b	Abcam	Cat#ab48394; RRID:AB881433; IF-1:300
Rabbit anti-LAMP1	Abcam	Cat#ab24170; RRID:AB_775978; IF-1:1000
Mouse anti-LAMP1 (LY1C6)	ThermoFisher Scientific	Cat#MA1-164; RRID:AB_2536869; IF/STED-1:300
Mouse anti-Dynein intermediate chain	Merck Millipore	Cat #MAB1618; RRID:AB_2246059; WB-1:4000
Rabbit anti-Rap1 mAb	Cell signaling	Cat#8825;RRID:N/A; WB/IF-1:500/1:300
Rabbit anti-Snapin	Synaptic Systems	Cat#148 002, RRID:AB_887796; WB/IF-1:500;1:300
Guinea pig anti-Synapsin 1/2	Synaptic Systems	Cat#106004; RRID:AB_1106784; IF-1:1500
Mouse anti-mCherry	Novus Biologicals	Cat#NBP1-96752; RRID:AB_11034849; IF-1:100
Rabbit anti-pSynapsin (pS62)	ThermoFisher Scientific	Cat#PA5-38336; RRID:AB_2554937; IF-1:1000

Rabbit anti-CHMP4b	Abcam	Cat#ab105767; RRID:AB_10858466; IF: 1:1000
Mouse anti-Tau1 (PC1C6)	Merck Millipore	Cat#MAB3420; RRID:AB_94855; IF-1:300
Rabbit anti-Tau	Synaptic Systems	Cat#314002; RRID:AB_993042; IF-1:300
Anti-doublecortin (DCX)	Abcam	Cat#ab18723; RRID:AB_732011; IF-1:500
Mouse anti-AP2	BD Biosciences	Cat#610501; RRID:AB_397867; IF-1:200
Mouse anti-MAP2 (HM-2)	Sigma Aldrich	Cat#M-4403; RRID:AB_477193; IF-1:500
Mouse anti-GM130 clone 35/GM130	BD Bioscience	Cat#610822; RRID:AB_10015242; WB-1:500
Rabbit sera anti-ProSAP2	kind gift from Prof. Böckers[2]	N/A; WB-1:500
Rabbit sera anti-ProSAP1P	kind gift from Prof. Böckers[3]	N/A; WB-1:500
Rabbit anti-PSD-Zip70	kind gift from Dr. Kenji Sobue [4]	N/A; WB-1:1000
Mouse anti-Rap1 and Rap2	BD Bioscience	Cat#610196; RRID:AB_397595; WB-1:1000
Mouse anti-Thy1	BD Bioscience	Cat#550571; RRID:AB_2201306; WB-1:1000
Mouse anti-Intein (CBD)	NE BioLabs	Cat#E8034S; WB-1:100
Rabbit anti-RFP	Rockland	Cat#600-401-379; RRID:AB_2209751
Mouse anti-GFP (3E6)	Molecular probes	Cat#A11120; RRID:AB_221568
Rabbit anti-His-tag	Cell signaling	Cat#2365; RRID:AB_2115720; WB-1:500
Mouse anti-His-tag (27E8)	Cell signaling	Cat#2366S; RRID:AB_10698449; WB-1:500
Mouse anti-β-actin (AC-15)	Sigma	Cat#A-5441; RRID:AB_476744; WB-1:500
Mouse anti-MBP	NE BioLabs	Cat#E8032S;WB-1:500
Rabbit anti-GST	Sigma	Cat#G7781; RRID:AB_259965;WB-1:500
Anti-mouse-AlexaFluor 405	ThermoFisher Scientific	Cat#A-31553; RRID:AB_221604; IF-1:300
Anti-mouse-AlexaFluor 488	ThermoFisher Scientific	Cat#A-11001; RRID:AB 2534069; IF-1:300
Anti-mouse-AlexaFluor 568	ThermoFisher Scientific	Cat#A-11004; RRID:AB_2534072; IF-1:300
Anti-mouse-AlexaFluor 647	ThermoFisher Scientific	Cat#A-21235; RRID:AB_2535804; IF-1:300
Anti-rabbit-AlexaFluor 488	ThermoFisher Scientific	Cat#A-11034; RRID:AB_2576217; IF-1:300
Anti-rabbit-AlexaFluor 568	ThermoFisher Scientific	Cat#A-11036; RRID:AB_10563566; IF-

		1:300
Anti-rabbit-AlexaFluor 647	ThermoFisher Scientific	Cat#A-21236; RRID:AB_2535805; IF-1:300
Anti-guinea pig-AlexaFluor 488	ThermoFisher Scientific	Cat#A-11073; RRID:AB_2534117; IF-1:300
Anti-goat-AlexaFluor 568	ThermoFisher Scientific	Cat#A-11057;RRID:AB_2534104; IF/STED -1:200
Anti-guinea pig-AlexaFluor 568	ThermoFisher Scientific	Cat#A-11075; RRID:AB_2534119; IF-1:300
Anti-guinea pig-AlexaFluor 647	ThermoFisher Scientific	Cat#A-21450; RRID:AB_2535867; IF-1:300
Anti-rabbit-Abberior STAR 580	Abberior	Cat#2-0012-005-8; IF/STED-1:200; IF-1:200
Anti-mouse-Abberior STAR 580	Abberior	Cat#2-0002-005-1; RRID:AB_2620153; IF/STED-1:250
Anti-mouse-Abberior STAR 635P	Abberior	Cat#2-0002-007-5; IF/STED-1:250
Anti-guinea pig-Abberior STAR 635P	Abberior	Cat#2-0112-007-1; IF/STED-1:250
Anit-goat-IgG-HRP	Dianova	Cat#705-035-147; RRID:AB_2337022 1:1000
Anti-rabbit-IgG-HRP	Dianova	Cat#111-035-114; 1:1000
Anti-mouse-IgG-HRP	Dianova	Cat#115-035-146; 1:1000
Anti-guinea pig-IgG-HRP	Dianova	Cat#705-035-148; 1:1000
4',6-diamidino-2-phenylindole (DAPI)	Biozol	Cat#BCL-BCFA-211; 1:1000
**Peptides and oligonucleotides**		**Source**
TAT-SIPA1L2- YGRKKRRQRRRPKKDCSKDLACKTLGGK		Genosphere biotechnologies
TAT- Scr- YGRKKRRQRRRPALKLCSKTDDKCKGGK		Genosphere biotechnologies
Generation of sipa1l2 mutant mice: 3armF, GAGGTAAGAAACTGCTGTTGGTCACTG		This paper
Generation of sipa1l2 mutant mice: 3armR, ACACACTGTACATACCCCTTGTTCATC		This paper
Generation of sipa1l2 mutant mice: 5armF: CTCCCTCCATCCCTTGTATGCCATCAC		This paper
Genotyping: musHetR ATGGGACCACATAACCGCCTCTCAGTG		This paper
Genotyping: Asc306 AATGGCCGCTTTTCTGGATTCATCGAC		This paper
Sy_EKAR cloning: SIPA_52AAGCGGCCGCAGATACT		This paper
Sy_EKAR cloning: SIPA_51: CGGATCCA(GGTGGCGGTGGA) ATGGCCTCCTCCGAGGAC		This paper
shRNA targeting sequence for Snapin KD GATCCGTGACAACCTAGCTACAGAA		[5]
shRNA targeting sequence for Snapin scrambled ATCCGCTGACCTCCAGAAATAAGA		This paper
**Recombinant DNA**		**Source**
Mouse Syp-(Myc-DDK-tagged)-synaptophysin		Origene MR204364;NM009305
EKAR-GFT/RFP (ERK-activity FRET sensor)		Addgene #18680
Rat SyGCaMP2		Addgene #26124

Rat EGFP-LC3b	Addgene #21073
Mouse GFP-fl-SIPA1L2-1-1722 (pEGFP-C1, Clontech)	[1]
Human His-LC3b	Addgene #73949
Mouse GFP-fl-SIPA1L2-N705A	This paper
Mouse fl-SIPA1l2-F638A-L641A-mCherry (LIR-motif mutant: EEFxxLL to EEAxxAL)	This paper
Mouse SIPA1L2-1-86-100-1722-mCherry (Δ14)	This paper
Mouse SIPA1L2-RapGAP-470-838-tRFP (pTag-RFP-N1, EVROGEN)	This paper
Mouse SIPA1L2-PDZ-948-1025-tRFP	This paper
Mouse SIPA1L2-RapGAP+PDZ-470-1025-tRFP	This paper
Mouse Intein-SIPA1L2-470-1025 (PMXB10)	This paper
Mouse Intein-SIPA1L2-470-1025-S990D (PMXB10)	This paper
Mouse Intein-SIPA1L2-RapGAP-470-838 (PMXB10)	This paper
Rat fl-TrkB-GFP	Kind gift from Prof. Ursula Wyneken
Mouse GFP-fl-Snapin	Snapin was cloned from cDNA library.
Rat 6xHis-Rap1b (pET28)	This paper

Supplementary table 2. Sequence and names of the primers used in this study and the corresponding contructs. In white boxes are forward primers and in blue reverse primers.

Contruct	Sequence (5‘ to 3‘)	Primer name
SNAPf-fl-SIPA1L2-1-1722	GAGCTAGCGCCACCATGGACAAAGACTGCG	SIPA_01
	CGTTCGGACCCGACCCAGGAGCTCTG	SIPA_02
fl-SIPA1L2-1-1722-mCherry	CGCTAGCGCTACCGGACTCAGATCATGAGTGATCCAAGGCCATCA	SIPA_03
	GATCTAGAGTCGCGGCCGCTTTACTTGTACAGCTCGTCCATGCC	SIPA_04
GFP-fl-SIPA1L2-N705A	GCACATCGGGGCCGACATCGTC	SIPA_05
	CCGTCGTCGAAGACTCCTTCTC	SIPA_06
fl-SIPA1l2-F638A-L641A-mCherry	GGCCTTCGAGGAGGCCCTGGACGCCCTCGGCCAGCGA	SIPA_07
	TCGCTGGCCGAGGGCGTCCAGGGCCTCCTCGAAGGCC	SIPA_08
GFP-RapGAP-625-813	GAGAATTCATGTACAACAACGAGACAG	SIPA_09
	ACGGATCCCGTAAATCCTTTAAGTATTCAT	SIPA_10
SIPA1L2-RapGAP-470-838-tRFP	GATCTCGAGCTCAAGCTTCTGCCACC ATG GAGAAGGTCAAACGTTAC	SIPA_11
	CTCTTCGCCCTTAGACACTGGATCCACCTTCTTCGCCCCCAGGGTG	SIPA_12
SIPA1L2-PDZ-948-1025-tRFP	GATCTCGAGCTCAAGCTTCTGCCACC ATG GAGACTGTGGAAATG	SIPA_13
	CTCTTCGCCCTTAGACACTGGATCCACCTCATGGGGCTGGATG	SIPA_14
SIPA1L2-RapGAP+PDZ-470-1025-tRFP	5GATCTCGAGCTCAAGCTTCTGCCACC ATG GAGAAGGTCAAACGTTAC	SIPA_15
	CTCTTCGCCCTTAGACACTGGATCCACCTCATGGGGCTGGATG	SIPA_16
SIPA1L2-ActI-1-164-delta 88-99-mCherry	TGGGAGAGCCGTTCTCAGTCC	SIPA_17

	AGGCCACTCAGACACTCTGGC	SIPA_18
Intein-SIPA1L2-RapGAP-470-838	TTATATCATATGGAGAAGGTCAAACGTTACA	SIPA_19
	AAATATCTCGAGCTTCTTCGCCCCC	SIPA_20
Intein-SIPA1L2-470-1025	TTATATCATATGGAGAAGGTCAAACGTTACA	SIPA_21
	AAATATCTCGAGGGGCTGGATGATAACCACTTT	SIPA_22
Intein-SIPA1L2-470-1025-S990D	GATCGGCTTGTGGAGATCTGCAAG	SIPA_23
	CCCTTGGCGAAGGCCTGCCTTC	SIPA_24
MBP-TrkB-454-821	GATCGAGGGAAGGATTTCAGAATTCAAGTTGGCGAGACATTCC	SIPA_25
	GCTGAAAATCTTATCTCAGCCTAGGATGTCCAGG	SIPA_26
MBP-TrkB-454-465	GATCGAGGGAAGGATTTCAGAATTCAAGTTGGCGAGACATTCC	SIPA_27
	GCTGAAAATCTTATCTCAGCCTTTCATGCCAAAC	SIPA_28
fl-TrkB-SNAPf	CGGGATCCACCATGGACAAAGACTGC	SIPA_29
	CCGTTCGGACCCGACCCAACTAGATCTCG	SIPA_30
GFP-fl-Snapin-1-136	GAGAATTCATGGCCGCGGCTGGTTC	SIPA_31
	CCAAGAGGTTCGTTTATTCCTAGGCG	SIPA_32
GFP-Snapin-S50D	GTCAGAGAAGATCAAGTAGAG	SIPA_33
	CTCTACTTGATCTTCTCTGAC	SIPA_34
GFP-Snapin-S50A	GTCAGAGAA GCG CAAGTAGAG	SIPA_35
	CTCTACTTGCGCTTCTCTGAC	SIPA_36
6xHIS-Snapin	GGTCGCGGATCCGATGGCGGGGGCTGGTTC	SIPA_37
	GAGCTCGAATTCTTGCCTGGGGAGCCAG	SIPA_38
SNAPf-fl-LC3b-1-142	GCGGATCCATGCCGTCCGAGAAGAC	SIPA_39
	CTCGGTACGAACACTGAGCTCGC	SIPA_40
6xHis-Rap1b	TTCTGCCATATGCGTGAGTATAAGCTAGTCGTTC	SIPA_41

	TATGCAGGATCCTTAAAGCAGCTGACATGATGA	SIPA_42
GST-RapGAP	AGCTTAGAATTCCGGTACTGCAAAGCCGGGCAG	SIPA_43
	GAATTGGATCCGTAAATCCTTTAAGTATTCATGCCTT	SIPA_44
GST-SIPA1L2-PDZ	AGCTTAGAATTCCGGGAAATGACCCTGAGAAGGAAT	SIPA_45
	GAATTGGATCCGCTCATGGGGCTGGATGATA	SIPA_46
GST-ActII GST-SIPA1L2-ActII-1026-1650	GGTCGTGGGATCCCCGAATTCGCCCTTCACCATGAGGATGGCTCTCCC	SIPA_47
	CGTCAGTCAGTCACGATGCGGCCGCTCTATGATCGCTCCCCTGGTG-	SIPA_48
GST-SIPA1L2-ActI- 1-624	GGTCGTGGGATCCCCGAATTCGCCCTTCAC ATGAGTGATCCAAGGCC	SIPA_49
	CGTCAGTCAGTCACGATGCGGCCGCTCTATTCCTCTTCCGTGCTCTG	SIPA_50
Sy-EKAR	CGGATCCAGGTGGCGGTGGAATGGCCTCCTCCGAGGAC	SIPA_51
	AAGCGGCCGCAGATACT	SIPA_52
musHet	TGAAAATCTTGGCCCTGTGGCGGTCAG	SIPA_53
	ATGGGACCACATAACCGCCTCTCAGTG	SIPA_54
Asc306	AATGGCCGCTTTTCTGGATTCATCGAC	SIPA_55
3arm	GAGGTAAGAAACTGCTGTTGGTCACTG	SIPA_56
	ACACACTGTACATACCCCTTGTTCATC	SIPA_57
5armF	CTCCCTCCATCCCTTGTATGCCATCAC	SIPA_58

Supplementary references

1. Spilker C, Acuna Sanhueza GA, Bockers TM, Kreutz MR, Gundelfinger ED. SPAR2, a novel SPAR-related protein with GAP activity for Rap1 and Rap2. *J Neurochem* **104**, 187-201 (2008).

2. Bockmann J, Kreutz MR, Gundelfinger ED, Bockers TM. ProSAP/Shank postsynaptic density proteins interact with insulin receptor tyrosine kinase substrate IRSp53. *J Neurochem* **83**, 1013-1017 (2002).

3. Wendholt D, *et al.* ProSAP-interacting protein 1 (ProSAPiP1), a novel protein of the postsynaptic density that links the spine-associated Rap-Gap (SPAR) to the scaffolding protein ProSAP2/Shank3. *J Biol Chem* **281**, 13805-13816 (2006).

4. Konno D, *et al.* The postsynaptic density and dendritic raft localization of PSD-Zip70, which contains an N-myristoylation sequence and leucine-zipper motifs. *J Cell Sci* **115**, 4695-4706 (2002).

5. Wu CS, *et al.* Type VI adenylyl cyclase regulates neurite extension by binding to Snapin and Snap25. *Mol Cell Biol* **31**, 4874-4886 (2011).
