# Supplementary material for: SIPA1L2 controls trafficking and local signaling of TrkB-containing amphisomes at presynaptic terminals
Source: Nat Commun. 2019 Nov 29;10:5448. doi: 10.1038/s41467-019-13224-z (PMC6884526; doi:10.1038/s41467-019-13224-z)
Supplement: Supplementary file 4 — Description of Additional Supplementary Files [file 41467_2019_13224_MOESM4_ESM.docx]

**Description of Additional Supplementary Files**

File name: Supplementary Movie 1

Description: Live imaging performed in an axon of a neuron overexpressing TrkB-SNAP (+SiR647) (green) and fl-SIPA1L2-mCherry (magenta) and scr-shRNA. Displayed at 2Hz. Soma is on the left.

File name: Supplementary Movie 2

Description: Live imaging performed in an axon of a neuron overexpressing TrkB-SNAP (+SiR647) (green) and fl-SIPA1L2-mCherry (magenta) and Snapin-shRNA. Note the immobile vesicles within circles. Displayed at 2Hz.
