# Supplementary material for: SIPA1L2 controls trafficking and local signaling of TrkB-containing amphisomes at presynaptic terminals
Source: Nat Commun. 2019 Nov 29;10:5448. doi: 10.1038/s41467-019-13224-z (PMC6884526; doi:10.1038/s41467-019-13224-z)
Supplement: Supplementary file 2 — Peer Review [file 41467_2019_13224_MOESM2_ESM.pdf]

Reviewers' comments:

Reviewer #1 (Remarks to the Author):

The manuscript “SIPA1L2 controls trafficking and signalling of TrkB-containing amphisomes at presynaptic terminals” by Andres-Alonso et al describes the role of a RapGAP in mossy fibre synaptic plasticity and suggests this occurs by localising BDNF/TrkB-dependent Erk signalling.

This is a comprehensive study; the manuscript contains an immense amount of data. Unfortunately the experiments are not always carefully described. In addition the authors assume detailed knowledge of the Mossy fibre field, together making the data difficult to access for the non-expert.

For example, figures S2 h-m are not referred to in the text. In figure S3 there seems to be a consistent difference between the wt and ko animals, which may not test significant at this stage but should be acknowledged in the text.

Figure 1: please explain the drug treatments in the purple and grey shaded areas in a, g for the non-mossy fibre specialist either within the text, legend or methods. I presume these are isolating the Mossy fibre path?

Figures 2b/ S4b: The TrkB band is below 140kDa – could the authors show more of the blot to show both bands and highlight whether the association is with the full length (expected at ~145kDa) or truncated TrkB? The blot in 2C suggests truncated, which would lead to fundamentally different conclusions. The antibodies listed by the authors would all recognise both forms. This critically needs to be addressed.

I also have questions regarding the choice of tissues: Why the switch to cerebellum for the biochemistry in figure 2b, forebrain in figure 2c? And why the use of E18 hippocampal neurons for cultures if the behaviour suggests there is a dentate specific deficit and CA1 dependent behaviours are not affected? DG neurons are born postnatal and can be cultured and imaged.

Figure 3a – I assume this was a GFP-Snapin IP, in which case a GFP only would have been useful. Is the conclusion that the RapGAP and PDZ domains individually are sufficient for the interaction? Are both (input and IP) panels probed for both RFP and GFP? Labelling/ legend/ text are not clear.

Figure 3b - to demonstrate co-recruitment one needs to see localization without the recruiting probe, where is Snapin localized in absence of RapGAP-PDZ? In d, what is imaged here? Could you show individual frames that underlie the kymograph? What is the direction of movement: towards the cell center or periphery?

Figure 3g/ p5 – the text should acknowledge “high percentage of retrograde and stationary co-trajectories” (also p7). Also, what is the % normalised to, TrkB or SIPA1L2? Can you comment on the behaviour of the single positive vesicles for either marker?

Figure 3 j-m: Can you show efficiency of knockdown? Is the transport defect TrkB specific or are other cargoes affected? This would be important information for the interpretation of the experiment. Is anterograde TrkB transport affected? Also, in k the scrambled sh treatment appears to increase the % stationary vesicles by itself, compared to panel g – please comment.

P7/ figure 5a,b: are the boutons labelled from the transfected or neighbouring cells? Image suggests neighbouring? Is the stopping specific to the LC3 structures or widely observed across cargoes?

The LC3b/SIPA1L2 traces in 6d look more akin to the mutant than wt traces in 5b, and accordingly, the dwelling times do not match between figures, please explain the discrepancy. Was cLTP induced in

figure 5?

Figure 7d,e, could you show representative data? Is there evidence for reduced TrkB-Erk1/2 signalling in the SIPA1L2 ko mice? A Western blot comparison of wt and ko cultures to complement the data would strengthen the argument.

The EKAR in figure 8 data is interesting, what type of signal is generated +/- BDNF or TrkB-Fc? The comparison would be useful. Is the activity TrkB-dependent? Also, what is the time scale of visiting versus Erk activity? Is there an immediate increase in active Erk, and how long is it retained for after the amphisome moves on? What is happening in ko neurons?

Minor comments:

P3 - They claim that BDNF/TrkB are "sorted by yet unknown mechanisms", yet a number of previous studies have addressed exactly these mechanisms (eg see Deinhardt 2006, Philippidou 2011, Burk 2017, Moya-Alvarado 2018, Goto-Silva, 2019).

P3 – a supplemental staining showing SIPA1L2 distribution in the hippocampus would set the scene for the subsequent experiments and help the reader interpret the data.

P4 – should read "synaptophysin".

In figure 1g, what concentration of TrkB-Fc was used?

Figure S4a – it would be nice to see a marker that does not float in the DRM prep to show that not all proteins co-fractionate in this experiment.
It would be helpful to have the experimental timeline for the TAT peptide experiments in figure 2, along with some more detail. How quickly does the peptide act, and how long is it active for? How well does it diffuse in the tissue?

Please indicate directionality in figure 3f.

Figure 4d – explain use of intein-caldendrin.

Figure 4e – what does anti-CBD detect, which lane shows the control? Is Snapin supposed to be visible in the Ponceau stain? (I can see the arrow, but no corresponding band).

I almost wonder if two separate manuscripts would do all these data more justice as individual results could be discussed in greater detail.

Reviewer #2 (Remarks to the Author):

The manuscript describes experiments suggesting that in neurons, BDNF and TrkB (BDNF receptor) localize in vesicles that are also positive for RAB7 and LC3. Once these vesicles traffic along axons towards cell soma, they make stops and signal at presynaptic boutons. Signaling and inward transport are shown to require the Rap GTPase-activating (RapGAP) protein SIPA1L2, which directly binds to TrkB and Snapin in order to connect TrkB-containing vesicles to dynein. Association with LC3 is shown to regulate the RapGAP activity of SIPA1L2, which also regulates retrograde trafficking. During induction of presynaptic plasticity, the signaling vesicles are shown to dissociate from dynein at synaptic boutons, and this is suggested to enable local signaling and to promote transmitter release.

The topic is important, the authors have studied the issue down to its molecular mechanism, and the experiments are well planned and controlled, with only few exceptions.

Major

1. The main concern is that manuscript is loaded with detailed data. The authors use very sophisticated but complicated experimental approaches, which are explained in very compact form. This makes the manuscript difficult to follow to readers of such a broad scope journal as Nature Communications. If this is due to restriction of space by the journal, the authors could think of alternative approaches, e.g. dividing the story in two manuscripts.

2. Fig. 7h,i, and page 9, first paragraph. TrkB and Snapin are localized in all fractions, SIPA1L2 only in A1 fraction. LC3b is most abundant in top fraction, less abundant in A1 and even less abundant in A2 and L. Why are A1 and A2 indicated as autophagy fractions? This gradient is not very informative and could be omitted, or alternatively, replaced with a more convincing cell fractionation experiment.

3. Fig. S6d. The diameter of the vesicles shown is too small for conventional amphisomes. Further, amphisomes should have contents (cytoplasmic cargo and intraluminal vesicles, about 50-nm in diameter, delivered by fusion with multivesicular endosomes). However, the structures in S6d are empty. Therefore, it is questionable whether the structures in the images are amphisomes. Much more likely the structures shown are endosomes. One gold particle per organelle is also not convincing to demonstrate positive immunostaining, since no quantification is shown to demonstrate enrichment of the labeling. Although the immunofluorescence and live imaging data suggest that the vesicles of interest could be amphisomes, the EM data in Fig. S6d is contradictory. The EM data should be omitted or alternatively, significantly improved. Without convincing EM confirmation, it would be justified to call the vesicles as 'possible/putative amphisomes' positive for LC3, RAB7, and the other proteins studied.

4. Fig. 8f remains unclear. How are the pSyn/Syn ratio and bouton number visualized in the figure?

5. Using Ponceau as loading control is problematic, since Ponceau staining is not very sensitive. It does not reliably reveal equal loading.

Minor

Page 3, first paragraph. The authors should shortly mention how ERK connects to TrkB. Not all readers are familiar with these signaling pathways.

Abbreviations should be explained when first mentioned (for instance LTP on page 3, first paragraph). What is cLTP (page 8, second row)?

Page 4, last paragraph: typo: symaptotagmin

Reviewer #3 (Remarks to the Author):

This manuscript by Kreutz and colleagues is a really interesting piece of work that nicely links from the molecular mechanisms to functional physiology both in slices and in vivo. It identifies the essential role for SIPA1L2 in the regulation of TrkB signalling via amphisomes and implicates amphisomes in the modulation of neurotransmission at individual nerve terminals.

I understand the logic of starting with the SIPa1L2 KO function and then going through the binding data etc but this is a little incongruous with the final paragraph of the introduction which is structured the other way around and finishes with the KO. It may be easier to follow if they were both the same.

There is huge quantity of data presented in this study and the first few figures, particularly figures 2, 3 and 4, jump around a lot between the figures and the supplemental, making the logic harder to follow. It may be worth considering consolidating some of this data and simplifying the data presented.

The legends for the figures would benefit from more information as to exactly what system is being used for each panel because the authors use a range of different systems and multiple different cell lines/neuronal cultures within figures. For eg. in figure 2 are these granule neurons or are these hippocampal neurons? Figure 5 – what neurons are these? Are these rat or mouse?

Throughout the manuscript, the n#s are not clear in terms of what is the n (i.e. bit of axon, neuron, coverslip, or prep/animal) and which number has been used for the statistics eg kymographs in figure 5 only say from greater than 3 preps and in figure 6, the number of dots on the graph panel e look like a lot more than the n number listed on the graph label inset. In figure 8c-e the n# in the graph inset is between 8-16 but the legend says n>3 independent expt so were the stats calculated with n # on the graphs or 3? Are the 8-16 individual boutons where the half life was calculated or averages per coverslip or averages per independent expt? More detail in the methods/legend and consistency throughout the manuscript would make the distinction clear.

The authors show nicely that there is physiological effect on LTP which can be phenocopied with collation of BDNF in fig1 or incubation of a binding peptide which blocks TrkB and Sipa1L2, implicating this signalling cascade. Later, the authors show that the RapGAP domain of Sipa1L2 is also required for plasticity. It would be nice to show a rescue of the system in Sipa1L2 KO, possibly by activating the pathway downstream of Sipa1L2 – if the model is correct, would a peptide encompassing the Sipa1L2 binding site on LC3 activate the RapGAP activity of Sipa1L2 and therefore bypass sipa1L2? Alternatively, in the paper the authors show that TrkB localises and traffics with LC3, dynein and snapin in different combinations. If Sipa1L2 is required as the link between these, in neurons from sipa1L2 knock out, perhaps the TrkB would not. Likewise, in supplemental fig S5, colocalisation of LC3 and TrkB increases upon BDNF application. Does this still occur in sipa1L2 KO neurons? Sipa1L2 can also be expressed back in to look at rescue.
Apologies if I missed this somewhere but more information explaining the time course of the LTP experiment, the stimulation frequencies used to generate the LTP and a reminder of the rationale of the expt with the drug incubations would be helpful. As well as the differences in the last 10 minutes (presence of DCGIV) there are bigger differences in the period of time preceding this which is not mentioned.

A weakness of the biochemistry and localisation data is that there is a large quantity of descriptive information which is displayed as n=1. I do not doubt that the authors will have done these experiments multiple times nor doubt the author's conclusions due to weight of data supporting their logic as well as the convincing peptide electrophysiology in the slices using the peptide of the binding site but, it would be helpful for the authors to quantify at least the major findings of the binding data in the early figures as done for the later figures as well as present the information as representative of n= x experiments.

Likewise, the localisation data is often one illustrative image (eg fig 2, 5, sup S5) and so some sort of quantification would make this more convincing. The line scans show the same n=1 as the images and

so further or different quantification assessing colocalisation across multiple images would be more convincing.

The idea of showing the link between amphisome trafficking regulation and synaptic changes is really nice however, the quantification in terms of n# in figure 8 f and g is 10 fold different between sipa1l2+/+ + cherry and sipa1L2-/- + SIPA1L2Δ14-cherry. To make these conclusions more rigorous, this should be quantified by averaging the boutons per axon segment or coverslip ideally and averaging them, not just the averages of the boutons.

This applies also to the FM4-64 experiment detailed in fig 8 h-m. Further, this experiment compares the unloading of FM dye before and after a period of time allowing amphisome trafficking, however, unloading 1 and unloading 2 are not directly comparable since different vesicle pools are likely being monitored which could have different release rates. A better experiment would have been to reload with dye following the amphisome trafficking phase and then repeat the unload thus ensuring the same vesicle pools would be labelled. The rate of unloading is also a proxy of exocytosis and so a more direct method looking at exocytosis specifically could have allowed specific conclusions about vesicle release to be made. The details of the n#s in terms of independent experiments have also been overlooked in the legend.

Minor points:

Since many of these figures are reliant on overexpression of tagged proteins it would be good to see that the levels of overexpression of wt is overexpressed to the same extent as the relevant mutants eg. Fig 5 Sipa1L2 wt and RapGAP dead mutant.

The characterisation of synaptic plasticity of the sipa1L2 KO presented in supplemental fig3 although technically not significantly different as per statistical tests, do not look overlaid either. Describing these as "normal" in the manuscript should be more cautiously described since although not significantly different, they do not look like "normal" either. It is not clear what the n# tested in this are? Are these individual animals, slices or sweeps and therefore is the experiment powered enough to detect significant differences?

The RapGAP activity assay uses a binding assay as a proxy for activity – a little more explanatory detail on the basis of the assay would make the experiment easier to interpret.

The wt GFP-Snapin included in Fig 6 along with the phosphomimetics would have been a useful control to compare the difference with the phosphomimetics. Additionally, the stationary pool not compared to the wt or phosphonull.

The model proposed by the authors and illustrated in supplemental figure S7 is really nice – does it have to be in the supplemental? I think the discussion would benefit from this figure being in the main text.

Reviewers' comments:

Reviewer #1 (Remarks to the Author):

The manuscript "SIPA1L2 controls trafficking and signalling of TrkB-containing amphisomes at presynaptic terminals" by Andres-Alonso et al describes the role of a RapGAP in mossy fibre synaptic plasticity and suggests this occurs by localising BDNF/TrkB-dependent Erk signalling.
This is a comprehensive study; the manuscript contains an immense amount of data. Unfortunately the experiments are not always carefully described. In addition the authors assume detailed knowledge of the Mossy fibre field, together making the data difficult to access for the non-expert. For example, figures S2 h-m are not referred to in the text. In figure S3 there seems to be a consistent difference between the wt and ko animals, which may not test significant at this stage but should be acknowledged in the text.

Reply: We want to thank the reviewer for the positive comments and we believe that we could address his/her criticism. According to the reviewers's suggestions, we have improved the description of the experiments and provided comprehensive explanations for non-expert readers. Moreover, we have revised the flow of the result section and made changes to ease reading of the manuscript.

Concerning figures S2 h-m, we believe that the reviewer overlooked the reference in the main text (line 3 of the Results section). Please note that in the current version the panels the reviewer refers to have been rearranged within the figure in order to improve the transition between the main text and the supplementary figures following the advice of reviewer 3 (see below / in the current version they are Figure S2f-m). For figure S3, we think that the differences are not really consistent. For instance, figure S3c and S3e show the opposite result for both genotypes and the statistical analysis does not show a clear trend. In addition, the differences in figure S3a-b and d might appear larger than they are due to the scale used in the graphs.

Figure 1: please explain the drug treatments in the purple and grey shaded areas in a, g for the non-mossy fibre specialist either within the text, legend or methods. I presume these are isolating the Mossy fibre path?

Reply: We thank the reviewer for pointing this out since this is critical information that was missing in the previous version of the manuscript. Indeed, blue-shaded boxes represent the presence of the NMDA receptor blocker D-APV during baseline recordings and potentiation. Please note that mossy fiber (MF)-LTP is a presynaptic, NMDA receptor-independent form of plasticity. The grey-shaded boxes depict the use of the group II mGluR agonist DCG-IV, which suppresses synaptic transmission at the MF pathway. This agonist was used at the end of each experiment to control for input specificity. All this information is now included in the figure legend.

Figures 2b/ S4b: The TrkB band is below 140kDa – could the authors show more of the blot to show both bands and highlight whether the association is with the full length (expected at ~145kDa) or truncated TrkB? The blot in 2C suggests truncated, which would lead to fundamentally different conclusions. The antibodies listed by the authors would all recognise both forms.

Reply: We have tried to clarify this issue and to this end we have also included additional data. We had already described in the manuscript that the binding region of TrkB for SIPA1L2 is found in the juxtamembraneous region of TrkB. Therefore, both full-length and truncated forms of TrkB are expected to associate with SIPA1L2. The blot in Figure 2b has a relatively poor resolution (see MW markers) and we are not able to clearly distinguish both bands. Moreover, if the immunoreactivity would indeed only stem from the shorter isoform of TrkB it should be emphasized that this is then also the most prominent band in the input and it would be therefore not surprising to see the shorter isofrom as the main band in the IP. In order to clarify this crucial point along with another question from the reviewer (see the next point) we firstly overexpressed the fl-TrkB fused to GFP in HEK293-T cells and detected the overexpressed protein with an alternative antibody against TrkB (goat

polyclonal raised against Cys32-His429 amino acids of TrkB from R&D; see below panel b) to test for the antibody specificity. Accordingly, we repeated the coimmunoprecipitation experiments (Figure 2c, previously Figure 2b) where we show the association with the full length TrkB using a different set of antibodies: anti-TrkB (R&D, goat) for immunoprecipitation and anti-TrkB (R&D, rat) for immunodetection of the TrkB in precipitates and, hence, replaced Figure 2b. Additionally, we added the inputs indicating the efficiency of the TrkB detection with anti-TrkB (R&D, goat) antibodies. Although the employed antibodies are directed against the extracellular domain of TrkB, we and others have observed that different antibodies show different preferences for one of the two isoforms, as evidenced by different band intensities (Cazorla et al., 2011).

Accordingly, we have reprobed the membrane for the coimmunoprecipitation experimens with DIC with the goat polyclonal anti-TrkB antibodies (R&D) and we show the association of DIC with the full length TrkB (updated panel 2d).

*Additionally, we repeated reciprocal immunoprecipitation experiment with SIPA1L2 antibodies from rat hippocampus and, therefore, replaced the panel **b** in the Figure S4.*

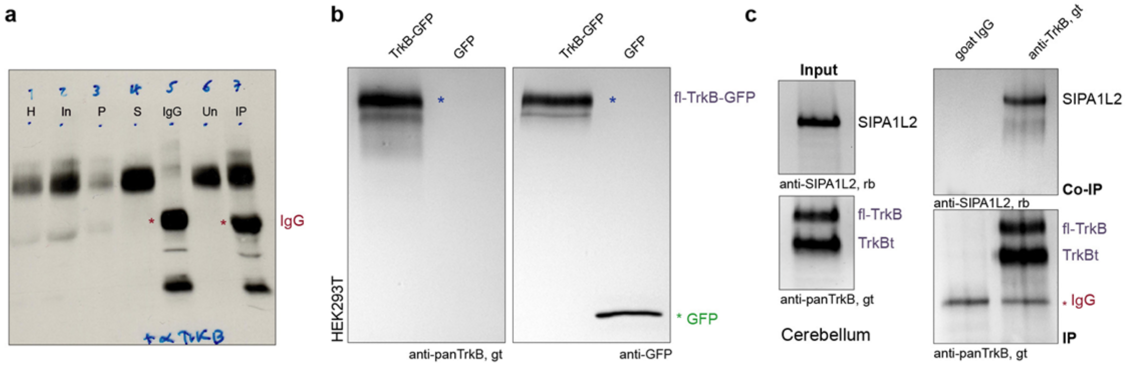

Figure legend: Endogenous TrkB co-immunoprecipitate SIPA1L2 from cerebellar lysates

*(**a**) represents the complete blot for the immunodetection of immunoprecipitated endogenous TrkB with anti-TrkB antibodies from lysates of cerebellum. Asteriks indicate fl-TrkB, truncated TrkB (TrkBt) and IgG. (**b**) Goat polyclonal anti-TrkB antibodies (R&D) raised against Cys32-His429 amino asids of TrkB specifically detect fl-TrkB-GFP extracted from HEK-293T cells. (**c**) Goat polyclonal anti-TrkB antibodies (R&D) detects both full length and truncated version of TrkB in extracts from cerebellum and can efficiently immunoprecipitate both isoforms.*

I also have questions regarding the choice of tissues: Why the switch to cerebellum for the biochemistry in figure 2b, forebrain in figure 2c? And why the use of E18 hippocampal neurons for cultures if the behaviour suggests there is a dentate specific deficit and CA1 dependent behaviours are not affected? DG neurons are born postnatal and can be cultured and imaged.

Reply: We acknowledge that the choice of tissues might be confusing. As described previously (Spilker et al., 2008) and also mentioned in the text, SIPA1L2 is most abundant in granule cells of the cerebellum and hippocampus. We have therefore used these regions for morphological analysis of the sipa1l2 ko mice (Figure S2e-k, m-o) and biochemical assays (previously Figure 2b; Figure S4a-b). Cerebellum was chosen because of the larger amount of tissue. Importantly, expression of SIPA1L2 is not restricted to these regions and we confirmed this by endogenous immunoprecipitation assays from different rat brain regions and immunostaining of primary hippocampal cultures (Figure 2a,c-f; Figure 3f; Figure 7a-b,d; Figure S5g-h; Figure S6d-e). Nevertheless, as explained in the previous section the endogenous coimmunoprecipitation experiments have been repeated with hippocampal tissue and the panel and figure legends have been accordingly exchanged (Figure 2c and Figure S4b). Please note that for the experiment in panel 2d (previously panel 2c), whole-brain and not forebrain was used. We apologize for this mistake and we have corrected it in the legend and main text.

Moreover, SIPA1L2 is present in pyramidal and granule cells and we believe that the cell biological mechanism under investigation is of general relevance. Of note, LTP at CA3-CA1 Schaffer collateral synapses that might be relevant for CA1-dependent behavior is expressed post- and not presynaptically, thus we don't expect a behavioral phenotype. Although studies have used hippocampal granule cells in dissociated cultures, these cultures do not solely contain granule cells but they are characterized by the enrichment of granule cells over pyramidal neurons (Baranes et al., 1996, Lopez-Garcia et al., 1996). However, DG neurons can't be easily kept in culture and, unlike hippocampal neurons in mixed primary cultures, do not resemble their in vivo morphology and do not reach a level of maturity like those in mixed hippocampal primary cultures. In such conditions, DG neurons also do not entirely acquire the morphological characteristics typical of the MF pathway, but instead resemble a younger and immature phenotype as compared to in-vivo conditions (Baranes et al., 1996). Therefore, even if we would have imaged in a co-culture system synapses where the postsynapse derives from pyramidal neurons, whereas the presynapse belongs to a granule cell, this will not necessarily resemble a mossy fiber synapse. Although MF boutons preferentially contact CA3 cells in vitro as well (Williams et al., 2011), their properties in culture systems differ (Lee et al., Neuron, 2013). Finally, we do not believe that the use of primary DG culture would change the outcome of these experiments.

Figure 3a – I assume this was a GFP-Snapin IP, in which case a GFP only would have been useful. Is the conclusion that the RapGAP and PDZ domains individually are sufficient for the interaction? Are both (input and IP) panels probed for both RFP and GFP?

Reply: The reviewer is correct. In the heterologous co-immunoprecipitation assay represented in Figure 3a GFP-Snapin was immunoprecipitated using magnetic beads coupled to anti-GFP antibodies. The membrane was firstly probed for Co-IP with anti-tagRFP (Evrogen) antibodies and then with anti-GFP for detection of equal immunoprecipitation of GFP-Snapin without stripping. We relabeled the IP as IP:GFP-Snapin. Because SIPA1L2 RapGAP+PDZ, as well as separated RapGAP- and PDZ-domains were fused to tagRFP, we have chosen tagRFP as a tag-related control for these Co-IPs (first line). Additionally, the inquired GFP control is depicted in the figure S5a where we examined the association of the RapGAP domain of SIPA1L2 with both GFP-tagged Snapin and GFP-tagged LC3b. These data suggest that both domains individually might be sufficient for the interaction with Snapin. In addition, we have clarified whether both (input and IP) have been probed for RFP and GFP in the Figure legend to better explain the loading scheme and the bands that we visualize.

Figure 3b - to demonstrate co-recruitment one needs to see localization without the recruiting probe, where is Snapin localized in absence of RapGAP-PDZ? In d, what is imaged here? Could you show individual frames that underlie the kymograph? What is the direction of movement: towards the cell center or periphery?

Reply: In response to the comment of the reviewer we have updated the panel in Figure 3b to show the localization of GFP-Snapin in the absence of RapGAP-PDZ-tRFP. We used tagRFP as control and co-expressed it together with GFP-Snapin in COS7 cells. In the absence of RapGAP-PDZ-tagRFP, GFP-Snapin shows a cytoplasmic distribution. We have repeated the GFP-Snapin/SIPA1L2-mCherry co-trafficking experiment in COS7 cells and as requested by the reviewer, we have also included in Figure 3d the individual frames from which the kymograph in Figure 3e was generated. The new panels 2d-e correspond to the sequential live-imaging of COS7 cells overexpressing GFP-Snapin and full-length SIPA1L2-mCherry and show the co-trafficking of these two proteins in the same complex. Please note that, though microtubule orientation in COS7 cells is plus-end out, microtubules are highly intermingled and often bend near the periphery of the cell. Therefore, we are unable in this case to make a statement on the directionality of the movement.

Figure 3g/ p5 – the text should acknowledge “high percentage of retrograde and stationary co-trajectories” (also p7). Also, what is the % normalised to, TrkB or SIPA1L2? Can you comment on the behaviour of the single positive vesicles for either marker?

Reply: Please note that graphs in Figure 3 (3h+k+m+o) represent the transport dynamics and directionality of co-trajectories. Accordingly, each co-trajectory is normalized to the total number of co-trajectories found in the corresponding axon and data is presented as a percentage of total co-trajectories. The total number of co-trajectories analyzed for each graph has been included in the figure legend. We have relabeled all graph axes accordingly to make data interpretation easier. We have included the analysis of co-trajectories as a percentage of total TrkB below for the reviewer. However, due to technical limitations, i.e. 1) the significant difference in signal-to-noise ratio between GFP (better GFP detection) and mCherry and 2) the likelihood of finding several TrkB-GFP particles per amphisome, we believe that this data is an underestimation of the real numbers and could provide misleading information for the readers. Finally, as requested by the reviewer, we have included a new panel (Figure 3i) showing the behavior of single TrkB and SIPA1L2 positive vesicles. The motility of both TrkB and SIPA1L2 is very similar, further supporting the direct interaction data described in the manuscript.

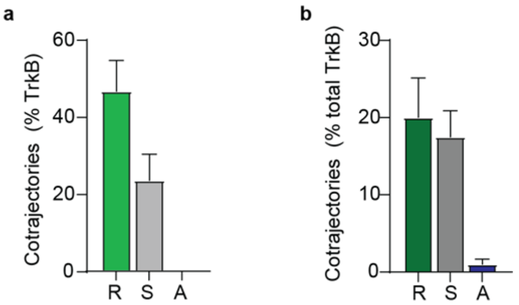

*Figure legend: Graph in (**a**) shows the number of cotrajectories as a percentage of relative TrkB trajectories (i.e. retrograde cotrajectories quantified per axon were normalized to total number of retrograde TrkB trajectories from the same axon). Graph in (**b**) shows cotrajectories per axon normalized to total number of TrkB trajectories from the same axon. R = retrograde; S = stationary; A = anterograde.*

Figure 3 j-m: Can you show efficiency of knockdown? Is the transport defect TrkB specific or are other cargoes affected? This would be important information for the interpretation of the experiment. Is anterograde TrkB transport affected? Also, in k the scrambled sh treatment appears to increase the % stationary vesicles by itself, compared to panel g – please comment.

Reply: We have employed a target sequence for Snapin KD that was described previously (Wu et al., 2011). For assessing the efficiency of the knockdown we co-transfected HEK-293T with 1:1 and 1:3 (shRNA:tagRFP-Snapin) ratios of either shRNA or scr-shRNA and tagRFP-Snapin. Results are depicted below (see panel a) and strongly suggest a high efficiency of the shRNA since the band for Snapin was dramatically reduced at 1:1 ratio and disappeared in conditions of shRNA of 3:1 co-transfection.

With regard to the cargo specificity of Snapin, others have demonstrated that absence of Snapin leads to a transport defect specific for late endosomes (Cai et al., 2010). Accordingly, axonal transport of mitochondria and early endosomes as well as ER and Golgi distribution is unaltered in neurons lacking Snapin (Cai et al., 2010). Nevertheless, we have checked for the axonal motility of peroxisomes (PEXs) in scr-shRNA and shRNA treated cells and found that absence of Snapin does not affect transport of PEXs in axons (see panel b). We also did not observe an impairment of TrkB anterograde transport (panel c). Please note that we have added two supplementary movies to the manuscript to further support the data set displayed in Figure 3l-m.

We have also looked into the differences among % stationary vesicles between Figure 3h and 3m (previously panels 3g+k) following the comment of the reviewer. Indeed this percentage is slightly higher in scr-shRNA treated cells which might be due to the longer expression time used in experiments requiring sh-RNA constructs (96h) vs trafficking assays (48h). However, this is statistically non-significant difference (see panel d).

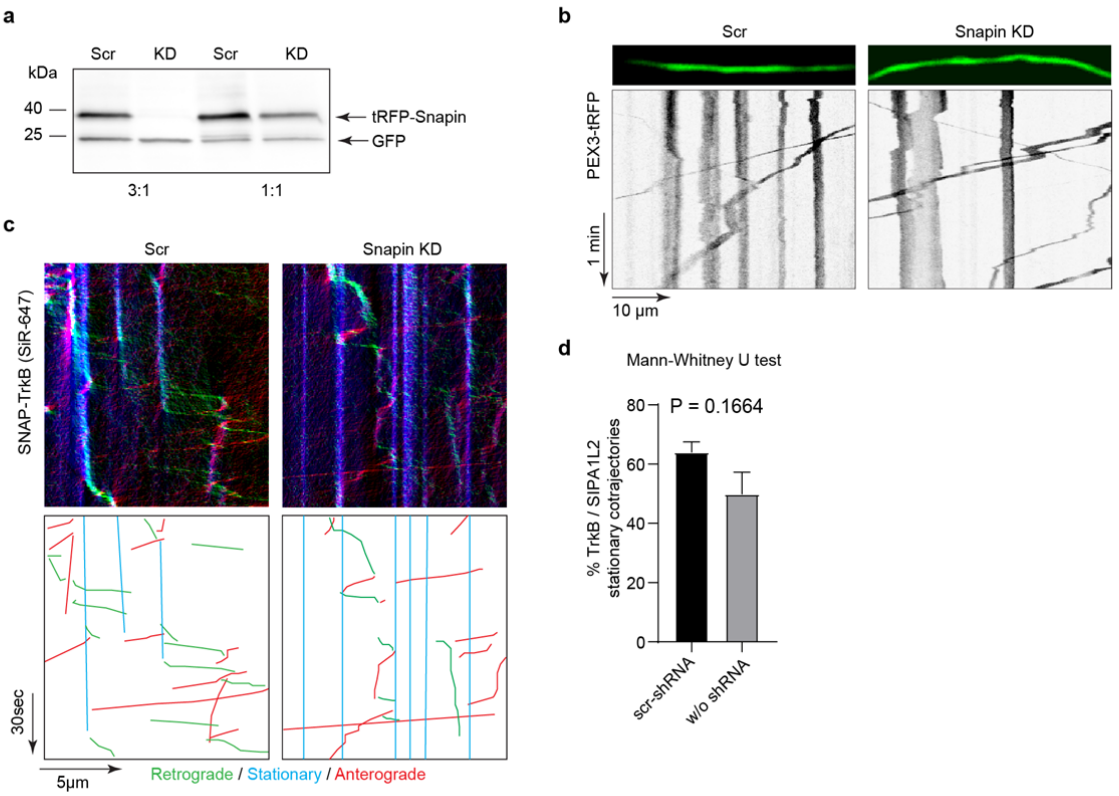

*Figure legend: (**a**) WB showing extracts from HEK293T cells co-transfected with tagRFP-Snapin and scramble-shRNA (Scr) or Snapin-shRNA (KD) at 3:1 and 1:1 (shRNA:tagRFP-Snapin) ratios. Please note that the shRNA vector is indicated in the figure as GFP. Membranes were probed with both anti-tagRFP and anti-GFP antibodies sequentially without stripping. (**b**) Representative kymographs and corresponding axons from rat hippocampal neurons transfected with the peroxisomal marker PEX3-tRFP and scr-shRNA or Snapin-shRNA. Note the similar motility of peroxisomes under both conditions. (**c**) Representative kymographs of prepared from primary hippocampal neurons overexpressing scr-shRNA or Snapin-KD and SNAP-TrkB. Trajectories are colour-coded by orientation. Note the presence of anterograde trajectories in both conditions. Below, sketch drawing showing manually-traced trajectories depicted in the kymographs. (**d**) Graph showing the direct comparison between the stationary trajectories quantified from Figure 3h (without shRNA; w/o shRNA) and 3h (scr-shRNA), previously panels 3g+k, corresponding to TrkB/SIPA1L2 cotrajectories under wt conditions (w/o-shRNA) or in scr-shRNA transfected neurons. Non-parametric Mann-Whitney U test was used for comparison.*

P7/ figure 5a,b: are the boutons labelled from the transfected or neighbouring cells? Image suggests neighbouring?

Reply: In this experiment, active presynaptic boutons were live-labeled using an anti-Synaptotagmin-1 antibody coupled to a fluorophore. In this assay all activated synaptic boutons are reliably labeled in the field of view. Data were generated by kymograph analysis and kymographs were created exclusively from transfected axons and the Synaptotagmin signal belonging to these axons. In consequence, we considered for analysis only boutons of the transfected axon. We have modified the panel 5a and we now represent analyzed ROIs in a different color (red) to discriminate them from those belonging to neighbouring cells (yellow). The figure legend is modified accordingly.

Is the stopping specific to the LC3 structures or widely observed across cargoes?

Reply: In order to address this important question, we monitored the dynamics in axons of peroxisomes (PEXs) and recycling endosomes (REs) using PEX3-Emerald and TfR1-GFP, respectively. Presynaptic boutons were labeled by anti-Synaptotagmin 1 antibody as described above and

*quantification is shown below. We found that only ~20% of the total moving PEX stopped at least once at boutons (**a**). Moreover, analysis of all stops revealed that PEXs and REs stops occur predominantly at non-synaptic sites (**b**). These results significantly differ from the analysis performed for SIPA1L2/LC3 stops showing that the majority of stops occur at boutons. Please note that these data are now included in a new panel in figure 5h. Therefore, we conclude that the stopping at synapses is prominent feature of amphisomes.*

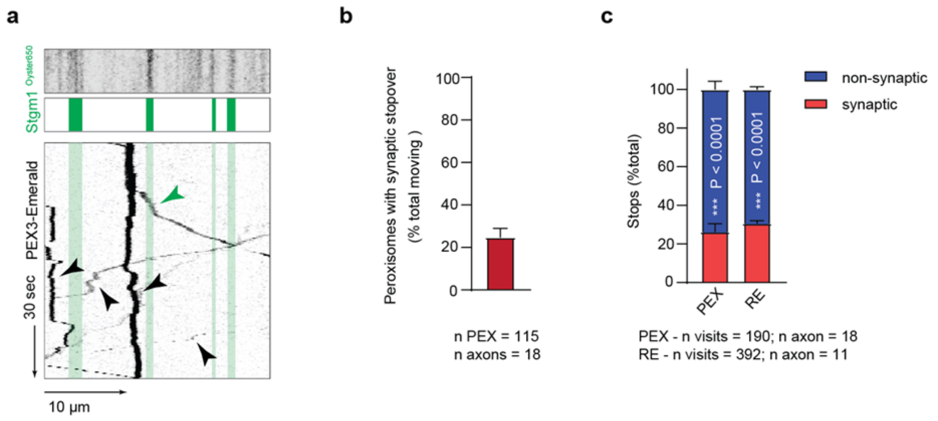

*Figure legend. (**a**) Representative kymograph generated from axons overexpressing PEX3-Emerald. Upper panel, stationary Stgm-1 Oyster 650 indicates presynaptic boutons. Black arrows indicate non-synaptic stopovers, green arrow indicates a synaptic stopover. (**b**) Quantification of the experiment described in (**a**) a percentage of peroxisomes that stop at least once in a bouton. (**c**) Quantification of the synaptic and non-synaptic stopovers of peroxisomes and recycling endosomes (RE) detected by overexpression of PEX-Emerald (PEX) and TfR1-GFP.*

The LC3b/SIPA1L2 traces in 6d look more akin to the mutant than wt traces in 5b, and accordingly, the dwelling times do not match between figures, please explain the discrepancy. Was cLTP induced in figure 5?

Reply: The differences can be explained by the fact that for cLTP experiments we had to use non-conditioned extracellular imaging buffer keeping in mind that the presence of the supplement in the culture medium might already enhance cAMP level at basal conditions. Experiments in Figure 5 were performed in neuronal conditioned medium. As expected, we observed a reduced dwelling time of the amphisome at synaptic boutons. We added explanatory information to the figure legend from both figures. The cLTP was not induced in experiments represented in figure 5.

Figure 7d,e, could you show representative data? Is there evidence for reduced TrkB-Erk1/2 signalling in the SIPA1L2 ko mice? A Western blot comparison of wt and ko cultures to complement the data would strengthen the argument.

Reply: We have included a panel with representative images in Figure 7d as suggested by the reviewer. The second point is difficult to address. TrkB-Erk1/2 signalling is local and changes will be subtle. Moreover, not all TrkB is bound by SIPA1L2 and the effect of the protein interaction is regulatory regarding signalling. Given that many pathways and signaling molecules other than TrkB are upstream of ERK activation a western blot analysis is unlikely to yield significant differences between genotypes.

The EKAR in figure 8 data is interesting, what type of signal is generated +/- BDNF or TrkB-Fc? The comparison would be useful. Is the activity TrkB-dependent? Also, what is the time scale of visiting versus Erk activity? Is there an immediate increase in active Erk, and how long is it retained for after the amphisome moves on? What is happening in ko neurons?

Reply: We thank the reviewer for the comment. We have performed further experiments in order to better characterize the Sy-EKAR signal following incubation with BDNF (100ng/ml for 30 min) or Fc-

TrkB (500ng/ml for >12h). Application of BDNF significantly decreased GFP lifetime compared to the GFP lifetime recorded in neurons incubated with Fc-TrkB bodies. Importantly, the magnitude of this reduction is similar to the decrease found upon the arrival of the amphisome at the terminals (Figure 8e) and it is prevented by preincubation with the tyrosine kinase inhibitor k252a (Schildt et al., 2013); 100nM, 30 min). These data demonstrate that ERK activation observed upon the visit of amphisomes is TrkB dependent and it further supports the presence of active TrkB in amphisomes.

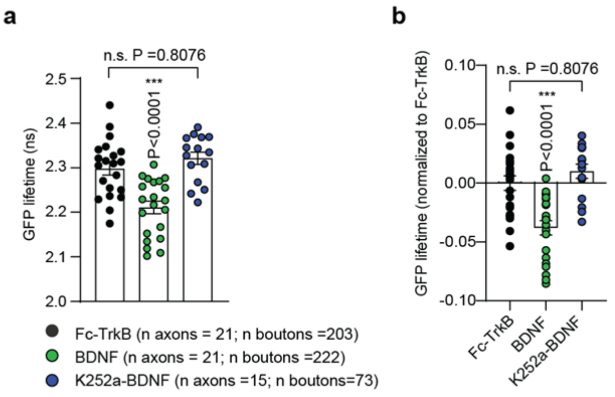

*Figure legend. (**a**) Lifetime of the Sy-EKAR donor (GFP) quantified from hippocampal neurons overexpressing Sy-EKAR. Neurons were treated with Fc-TrkB, BDNF or pretreated with the Trk inhibitor K252a (30 min) before and during the application of BDNF. Note that a decrease in donor lifetime (GFP lifetime) indicates higher FRET efficacy and activation of ERK. (**b**) Data presented in (**a**) normalized to Fc-TrkB. This representation is similar to the one present in Figure 8e. Circles represent average values per axon. Number of analyzed axons and total number of boutons is represented below the graph in **a**. One-way ANOVA with Bonferroni's correction test (n = axons).*

An analysis of Erk activity by using Sy-EKAR in sipa1l2 ko neurons would be interesting. However, we have shown that transport of amphisomes is impaired in the absence of SIPA1L2 (see Figure 3n-o) and amphisomes remain immobile. Under these conditions, the local and transient activation of Erk signaling triggered by the amphisome visit would be impossible to detect.

We plotted the time scale of Erk activity vs the visits in panels 8c-d. Estimation of time of arrival of amphisomes to the boutons was done by kymograph analysis (panel 8a). Frame of arrival was considered as time 0 in 8c-d. According to this analysis, the Erk activation occurs within seconds. This experiment was aimed to correlate visits to Erk activity. Because the duration of stopovers is variable and we have only analyzed the immediate 8 frames after arrival, we cannot make a statement on the duration of this Erk activation. We acknowledge that this is an interesting question but we believe it is out of the scope of this study.

Minor comments:

P3 - They claim that BDNF/TrkB are "sorted by yet unknown mechanisms", yet a number of previous studies have addressed exactly these mechanisms (eg see Deinhardt 2006, Philippidou 2011, Burk 2017, Moya-Alvarado 2018, Goto-Silva, 2019).

Reply: We have rephrased this statement to "sorted by different mechanisms"

P3 – a supplemental staining showing SIPA1L2 distribution in the hippocampus would set the scene for the subsequent experiments and help the reader interpret the data.

Reply: Our previous study describes SIPA1L2 mRNA distribution across different brain areas (Spilker et al., 2008). Unfortunately our antibodies are not really suitable for IHC.

P4 – should read "synaptophysin".

Reply: We are sorry for this mistake and we have corrected it accordingly.

In figure 1g, what concentration of TrkB-Fc was used?

Reply: As described in the method section (Electrophysiology, subsection MF-LTP), slices were incubated with 5µg/ml TrkB-Fc. We have now included this information in the figure legend.

Figure S4a – it would be nice to see a marker that does not float in the DRM prep to show that not all proteins co-fractionate in this experiment.

Reply: Not all markers float in this assay. For instance, ProSAPiP and ProSAP2/Shank but also Rap1 are not present in all fractions.

It would be helpful to have the experimental timeline for the TAT peptide experiments in figure 2, along with some more detail. How quickly does the peptide act, and how long is it active for? How well does it diffuse in the tissue?

Reply: The experimental timeline for the TAT peptide experiment is now in figure 2j. The corresponding images showing the distribution of the TAT peptide within the DG following infusion are present in Figure S4. To answer the reviewer comment, it is difficult to judge how quickly the TAT peptide begins to act. Based on our observations, the addition of the TAT peptide into the protein mix overnight disrupted the interaction of SIPA1L2 with TrkB (figure 2i), and this would indicate that it remains stable for longer periods of time. As described in materials and methods, we have assessed the effect of TAT-peptide infusion 24 h later in the choice phase of the pattern separation task, therefore supporting the idea that the TAT peptide might act over longer time intervals. Additionally, localization of the TAT peptide labelled with FITC shows a diffused distribution in DG, indicating that it can affect the interaction in a large number of cells.

Please indicate directionality in figure 3f.

Reply: The directionality of amphisome movements (now panel 3g) towards the soma is indicated in the figure with an arrow under the respective kymograph.

Figure 4d – explain use of intein-caldendrin.

Reply: Figure 4d represents the results of the pull-down assay where we show a direct association of the intein-tagged RapGAP domain of SIPA1L2 with His-tagged LC3b. We have used Intein-tagged Caldendrin as a negative non-binding control. This information has been included in the figure legend.

Figure 4e – what does anti-CBD detect, which lane shows the control? Is Snapin supposed to be visible in the Ponceau stain? (I can see the arrow, but no corresponding band).

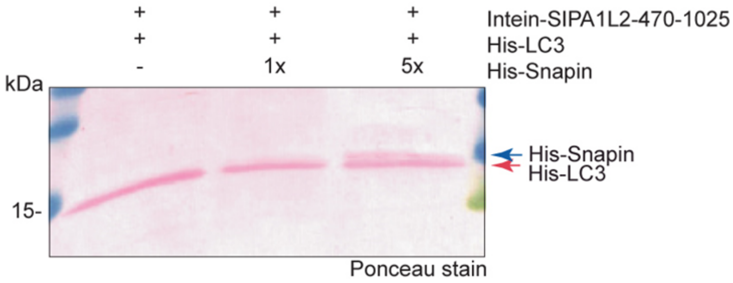

Reply: Figure 4e represents the pull-down assay where we show that inclusion of snapin into the SIPA1L2-(RapGAP+PDZ) / LC3b protein mix does not alter the association of SIPA1L2 with LC3b. The first line shows the positive control without Snapin. A negative control was not included in the experiment represented in 4e because it is already shown in figure 4d. Anti-CBD antibodies detect the Chitin Binding Domain within the Intein tag of SIPA1L2. For clarification, we modified the labeling in the figure as anti-Intein (CBD) and added an explanatory sentence to the figure legend.

We agree with the reviewer that the ponceau blot represented in figure 4e is not informative, therefore we decided to omit this image from the figure. The arrow was supposed to indicate the

band of His-tagged Snapin (see magnified figure) that is in very close proximity to the LC3b band in the PD due to similar molecular weight. During the figure preparation process, the label "input" got shifted from His-Snapin blot to Ponceau stain indicating the PD. We apologize for the mistake. We modified the panel 3e accordingly.

I almost wonder if two separate manuscripts would do all these data more justice as individual results could be discussed in greater detail.

Reply: As outlined above we have tried to streamline the manuscript. Accordingly, we have also reorganized figures with regard to the previous version to improve the flow and ease the reading and we have included more detailed experimental information in the main text and legends. We believe that it would be very difficult to separate the work into two different stories without significant overlap.

Reviewer #2 (Remarks to the Author):

The manuscript describes experiments suggesting that in neurons, BDNF and TrkB (BDNF receptor) localize in vesicles that are also positive for RAB7 and LC3. Once these vesicles traffic along axons towards cell soma, they make stops and signal at presynaptic boutons. Signaling and inward transport are shown to require the Rap GTPase-activating (RapGAP) protein SIPA1L2, which directly binds to TrkB and Snapin in order to connect TrkB-containing vesicles to dynein. Association with LC3 is shown to regulate the RapGAP activity of SIPA1L2, which also regulates retrograde trafficking. During induction of presynaptic plasticity, the signaling vesicles are shown to dissociate from dynein at synaptic boutons, and this is suggested to enable local signaling and to promote transmitter release. The topic is important, the authors have studied the issue down to its molecular mechanism, and the experiments are well planned and controlled, with only few exceptions.

Reply: We also want to thank reviewer 2 for the positive comments.

Major

1. The main concern is that manuscript is loaded with detailed data. The authors use very sophisticated but complicated experimental approaches, which are explained in very compact form. This makes the manuscript difficult to follow to readers of such a broad scope journal as Nature Communications. If this is due to restriction of space by the journal, the authors could think of alternative approaches, e.g. dividing the story in two manuscripts.

Reply: As outlined in our response to reviewer 1 we acknowledge the difficulty to follow the line of arguments in the previous version of the manuscript. We have tried to improve the description of the experiments in the main text and especially in figure legends and provided comprehensive explanations for non-expert readers. Moreover, we have revised the flow of the result section and made changes to ease reading of the manuscript. On our opinion it will be extremely difficult if possible at all to separate the work into two different stories without significant overlap.

2. Fig. 7h,i, and page 9, first paragraph. TrkB and Snapin are localized in all fractions, SIPA1L2 only in A1 fraction. LC3b is most abundant in top fraction, less abundant in A1 and even less abundant in A2 and L. Why are A1 and A2 indicated as autophagy fractions? This gradient is not very informative and could be omitted, or alternatively, replaced with a more convincing cell fractionation experiment.

Reply: We have revised this panel following the reviewers comment and found a misleading spelling mistake. We followed an established fractionation protocol (Mancias et al., 2014) with modifications. According to this protocol, fractionation results in A1 (autophagosome), A2 (autophagolysosome) and L (lysosome). Unfortunately, a labeling mistake was found in the panel where "Top" was meant to be

“Tot”, an abbreviation for total fraction. This explains why LC3b is most abundant in this fraction, as the reviewer pointed out. This labeling mistake has been corrected in the panel and figure legend. We thank the reviewers for drawing our attention to this and we apologize for the mistake.

This experiment was aimed to further support the presence of SIPA1L2 in the autophagosome compared to the lysosome fraction. We believe that this fractionation is useful to support our hypothesis as the autophagosomal A1 fraction is clearly different from the autophagolysosomal and lysosomal fractions (A2 and L), which might not be easy to distinguish using this approach. Note that SIPA1L2 and Rab7 are as expected only present in the A1 fraction and not in the A2 and L fractions. We have also rephrased the section in Results accordingly.

3. Fig. S6d. The diameter of the vesicles shown is too small for conventional amphisomes. Further, amphisomes should have contents (cytoplasmic cargo and intraluminal vesicles, about 50-nm in diameter, delivered by fusion with multivesicular endosomes). However, the structures in S6d are empty. Therefore, it is questionable whether the structures in the images are amphisomes. Much more likely the structures shown are endosomes. One gold particle per organelle is also not convincing to demonstrate positive immunostaining, since no quantification is shown to demonstrate enrichment of the labeling. Although the immunofluorescence and live imaging data suggest that the vesicles of interest could be amphisomes, the EM data in Fig. S6d is contradictory. The EM data should be omitted or alternatively, significantly improved. Without convincing EM confirmation, it would be justified to call the vesicles as 'possible/putative amphisomes' positive for LC3, RAB7, and the other proteins studied.

Reply: Unfortunately, it is impossible to control the amount of GFP fused TrkB molecules (a low DNA concentration for slice electroporation was used) incorporated into a single amphisome or to make any statements of preferential association of TrkB-GFP with MVB vs amphisome vs simple endosome. We, therefore, focused on double-membrane organelles that resemble the features of autophagosomes (as described in M&M) and immunogold label to ensure the amphisome identity of the organelle unequivocally. In the absence of a second specific amphisome marker, this can only be described qualitatively. There is a growing body of literature on the heterogeneity in size and the content of the autophagosome (Mancias et al., 2014, Nature letters). As an example, EM image performed from autophagosome fraction (Mancias et al., 2014) shows a high diversity in size and the vesicular content. On the other hand organelle identity, particularly in the endosomal and endolysosomal pathway, is uncertain at the EM level to some extent. This could lead to a significant underestimation of the number of amphisomes. On top, the angle of cutting and the relative positive to mossy fiber boutons will also make it difficult unambiguously identify amphisomes and in particular to distinguish them from mitochondria. Taking all this into account we have looked at all available EM images in search for structures that matched the criteria mentioned by the reviewer and we indeed found better examples that are now depicted in panel a below. These are large structures (>250nm) that contain cytoplasmic cargo and are surrounded by a double membrane. Importantly, the immunogold in both cases is located in the outer membrane of the organelle. Since the hippocampal slices were electroporated with a TrkB fused to a GFP in its C terminus and the immunogold labeling was performed against GFP, the gold particles would indicate the C terminal region of TrkB that contain the signaling domain. Please note that the gold particles are only present in these and not in other organelles in these images.
Additionally, we have also tried an alternative approach in order to improve the quality of the EM data as requested by the reviewer. To this end, we transfected primary rat hippocampal neurons that were plated on 12mm punched ACLAR embedding Film (Ted Pella, Inc. USA) for 10DIV with TrkB-GFP and performed immunogold labeling against GFP (panel b). In these experiments the embedding was done with methacrylate without osmium fixation in order to preserve the antigenicity and the fluorescence required to identify transfected cells in confocal microscopy and to allow the postembedding immunogold labeling. This procedure was done at the expense of losing the lipid membrane structures and contrast. We found structures with features of amphisomes as they

resemble autophagosomes in the sense that their slightly altered cytoplasmic content and could contain different cytoplasmic packages as a result of repeated endosomal fusions, similarly to what others found in different cell types (Berg et al., 1998, Patel et al., 2013). Importantly, they are larger than conventional autophagosomes and are heavily labeled by gold particles that are not only intraluminal but they are also found in the outer regions.
We believe that these are examples of neuronal amphisomes that support our immunofluorescence and live imaging data. However, our antibodies against LC3 were proven to not be suitable for immunogold-EM and the lipid membrane is not well preserved. We can't therefore provide compelling evidence for this notion. Given these shortcomings we decided at this stage to omit the EM data from the manuscript. However, if the reviewer feels that the data are convincing enough to be included we will be happy to modify the corresponding figure.

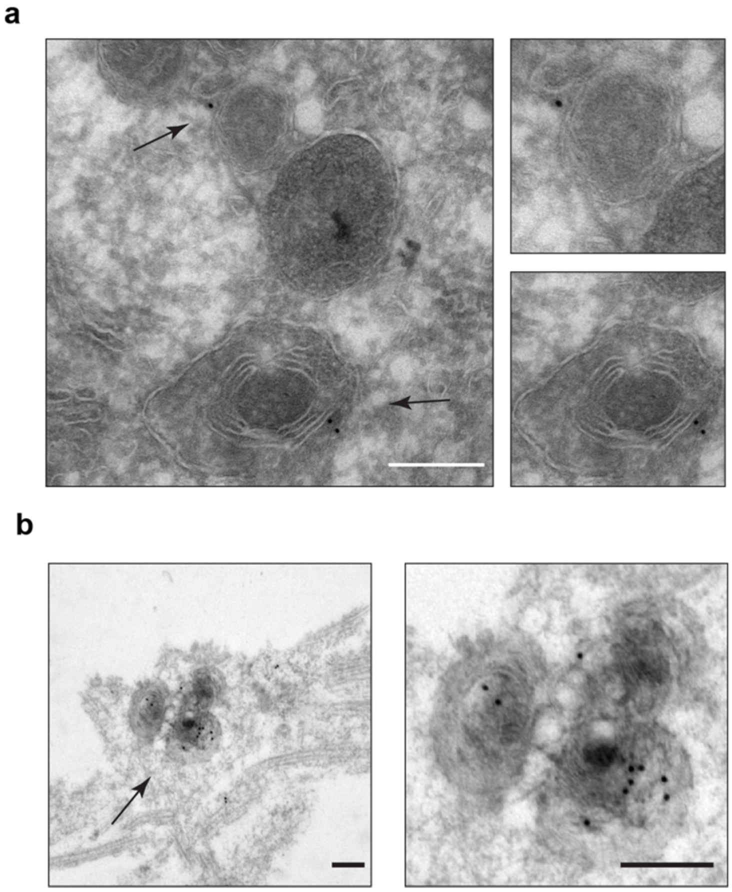

*Figure legend: (**a**) Immunogold EM images obtained from mossy fiber synapses of mouse hippocampal organotypic slices electroporated with TrkB tagged with GFP in the C terminus. Immunogold was performed with an antibody directed against GFP. (**b**) Representative images (left: overview; right: zoom-in) obtained after postembedding immunogold labeling performed from rat primary cultures at DIV 10 transfected with TrkB tagged with GFP in the C terminus. Immunogold was performed against GFP. Structures indicated by the arrows are shown in the right panels. **PLEASE NOTE that samples shown in both panels are generated from two different preparations (organotypic slices in a and primary cultures in b) where lipid membranes are preserved differently due to extraction of lipids in B and different processing of the samples.** Scale bars are 250nm in a-b.*

4. Fig. 8f remains unclear. How are the pSyn/Syn ratio and bouton number visualized in the figure?

Reply: We apologize because the information was not clear. Representative images in figure 8 show intensity of fluorescence of $sipa1l2^{+/+}$ *or* $sipa1l2^{-/-}$ *primary hippocampal neurons (genotype is depicted below the images) transfected with tagRFP, SIPA1L2-mCherry or SIPA1L2-Δ14-mCherry, treated with*

Fc-TrkB or BDNF and immunostained for pan-Synapsin or pSyn62. Black arrows indicate boutons within transfected axons. Quantification of changes in fluorescence intensity is depicted in figure 8g. The graph shows the pSyn/Syn ratio for each group of BDNF-treated cells as a percentage of the ratio obtained from the corresponding Fc-TrkB group. In the previous version, the average value calculated from the number of boutons was used for the graph and statistics. Following the suggestion from reviewer 3 (see below), we have averaged the boutons per axonal segment and we present now a graph with the average from all axons quantified. The information has been added to the figure legend together with the number of axons used for the Fc-TrkB treated groups.

5. Using Ponceau as loading control is problematic, since Ponceau staining is not very sensitive. It does not reliably reveal equal loading.

Reply: We used this approach for labeling of recombinant GST-RalGDS for normalization only when we were using very high protein concentrations (figure 4g+i). Figures 4g+i represent the result of the pull-down assays performed from whole cell extracts overexpressing wild type SIPA1L2 as well as RapGAP (SIPA1L2-N705A) and LIR (F638A-L641A) mutants. As evident from the results, 24 hours overexpression of wild type SIPA1L2 in HEK-293T cells completely abolished association of Rap1 with GTP (a small background of Rap1-GTP in graphs is coming from the non-transfected cells). The introduced mutations abolished RapGAP activity of SIPA1L2 resulting in a high association of GTP-bound Rap1 with GST-RalGDS. This is the major factor that accounts for the difference observed. Therefore, we are confident that for these two particular experiments the sensitivity of Ponceau is sufficient to detect the GST-RalGDS bands and differences in the detection of GTP-Rap1 normalized to GST-RalGDS detected with antibodies versus ponceau are negligible.

The Ponceau staining in the figure 4k was not used for normalization but rather for detection of untagged RapGAP domain of SIPA1L2 that otherwise would be undetectable due to the absence of the antibody recognizing of this particular fragment of SIPA1L2. It also detects 6xHis-SUMO protein used as a control for 6xHis-ragged LC3b detected with anti-LC3 antibodies.

For in vitro RapGAP assays where we assess the modulation of RapGAP activity in the presence of the LC3b (Figure 4k) as well as by S990 phosphorylation (figure 6h) quantification was performed based on the antibody detection of GST-RalGDS.

Ponceau staining was also used in Figure 2h for detection of GST-SIPA1L2-86-99, and in Figure 4c for detection of RapGAP and PDZ domains for corresponding pull-down assays and has not been used for normalization.

Minor

Page 3, first paragraph. The authors should shortly mention how ERK connects to TrkB. Not all readers are familiar with these signaling pathways.

Reply: We have better explained the molecular link between TrkB signaling and ERK in the text and we have also included a small explanatory cartoon in Figure S1a.

Abbreviations should be explained when first mentioned (for instance LTP on page 3, first paragraph). What is cLTP (page 8, second row)?

Reply: We thank the reviewer for pointing this out. We added the explanations for both in the main text.

Page 4, last paragraph: typo: symaptotagmin

Reply: We have corrected this typo.

Reviewer #3 (Remarks to the Author):

This manuscript by Kreutz and colleagues is a really interesting piece of work that nicely links from the molecular mechanisms to functional physiology both in slices and in vivo. It identifies the essential role for SIPA1L2 in the regulation of TrkB signalling via amphisomes and implicates amphisomes in the modulation of neurotransmission at individual nerve terminals.
I understand the logic of starting with the SIPa1L2 KO function and then going through the binding data etc but this is a little incongruous with the final paragraph of the introduction which is structured the other way around and finishes with the KO. It may be easier to follow if they were both the same.

There is huge quantity of data presented in this study and the first few figures, particularly figures 2, 3 and 4, jump around a lot between the figures and the supplemental, making the logic harder to follow. It may be worth considering consolidating some of this data and simplifying the data presented.

Reply: We thank the reviewer for the positive feedback and we have worked on his/her suggestions (see also the replies to Reviewer 1 and 2). We believe that we have significantly improved the flow of the manuscript, explanation of experiments and figure legends. Altogether, we present a more consolidated manuscript that is hopefully more accessible to a broader readership.

The legends for the figures would benefit from more information as to exactly what system is being used for each panel because the authors use a range of different systems and multiple different cell lines/neuronal cultures within figures. For eg. in figure 2 are these granule neurons or are these hippocampal neurons? Figure 5 – what neurons are these? Are these rat or mouse?

Reply: We thank the reviewer for this criticism. We have updated the requested information in the figure legends accordingly. We have used primary hippocampal cultures throughout the manuscript (see reply to reviewer 1). Accordingly, in both Figure 2 and 5 rat primary hippocampal cultures were used. All this information is now included in the corresponding figure legends.

Throughout the manuscript, the n#s are not clear in terms of what is the n (i.e. bit of axon, neuron, coverslip, or prep/animal) and which number has been used for the statistics eg kymographs in figure 5 only say from greater than 3 preps and in figure 6, the number of dots on the graph panel e look like a lot more than the n number listed on the graph label inset. In figure 8c-e the n# in the graph inset is between 8-16 but the legend says n>3 independent expt so were the stats calculated with n # on the graphs or 3? Are the 8-16 individual boutons where the half life was calculated or averages per coverslip or averages per independent expt? More detail in the methods/legend and consistency throughout the manuscript would make the distinction clear.

Reply: We have included information regarding n numbers throughout the manuscript. For cotrajectory analysis, statistics are done using the number of axons (Figure 3 and 5). For quantification of dwelling time (Figure 5j and 6e) or visits (Figure 8c), statistics are done using the number of events or boutons analyzed. We are sorry that this information was unclear. We have updated figures and figure legends to clarify this point.

The authors show nicely that there is physiological effect on LTP which can be phenocopied with collation of BDNF in fig1 or incubation of a binding peptide which blocks TrkB and Sipa1L2, implicating this signalling cascade. Later, the authors show that the RapGAP domain of Sipa1L2 is also required for plasticity. It would be nice to show a rescue of the system in Sipa1L2 KO, possibly by activating the pathway downstream of Sipa1L2 – if the model is correct, would a peptide encompassing the Sipa1L2 binding site on LC3 activate the RapGAP activity of Sipa1L2 and therefore bypass sipa1L2?

Reply: We agree with the reviewer that an in-vivo rescue of the sipa1l2 ko mice would be very interesting. However, as we discuss in the manuscript, our data indicate that deficits found in sipa1l2 ko mice are due to the lack of TrkB-mediated ERK activation at single presynaptic boutons. This mechanism serves local purposes likely at specific time points. Any pharmacological intervention would imply a broad and tonic activation of the downstream cascade. We strongly believe that this approach would cause numerous off-target effects and make the interpretation of data difficult. As suggested by the reviewer, the use of a TAT peptide could be a more elegant approach. However, this would not be a rescue experiment as a TAT peptide encompassing the SIPA1L2 binding site on LC3 will indirectly affect many other LC3 targets with a LIR motif. Thus, the outcome of this experiment will likely not add relevant information to the manuscript.

Alternatively, in the paper the authors show that TrkB localises and traffics with LC3, dynein and snapin in different combinations. If Sipa1L2 is required as the link between these, in neurons from sipa1L2 knock out, perhaps the TrkB would not. Likewise, in supplemental fig S5, colocalisation of LC3 and TrkB increases upon BDNF application. Does this still occur in sipa1L2 KO neurons? Sipa1L2 can also be expressed back in to look at rescue.

Reply: It has been shown previously that dynein-dependent retrograde trafficking of TrkB-signaling endosomes essentially requires an association with the adaptor protein Snapin (Zhou et al., 2012), though the characteristics of this association remained unclear. Our data show that retrograde trafficking of TrkB in Snapin shRNA - expressing cells is impaired and that in SIPA1L2 KO neurons, TrkB/LC3 complexes remain immobile. These results strongly suggest that one of the roles of SIPA1L2 is to serve as a cargo adaptor for Snapin. This is further supported by our results showing the direct association between these two proteins. Another crucial role of SIPA1L2 is to control the local signaling of TrkB at boutons via the modulation of its RapGAP activity by LC3. Colocalization analysis of TrkB with LC3 in SIPA1L2 wt and ko neurons suggests that SIPA1L2 is not required for the association of TrkB and LC3 in amphisomes (n numbers correspond to the number of images analyzed). Additionally, both proteins colocalize as immobile structures in SIPA1L2 KO neurons. Therefore, we believe that SIPA1L2 may not play a role in the formation of the amphisome but in its signaling properties. Likely, the colocalization of LC3/TrkB upon BDNF in SIPA1L2 ko neurons is increased as in wt cells (data not shown).

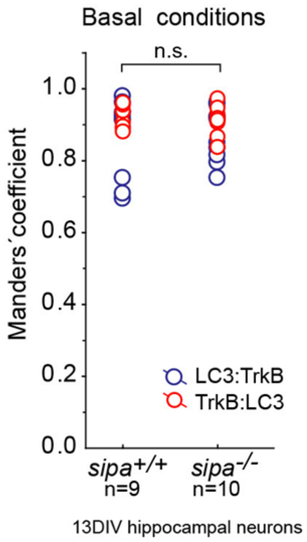

Figure legend: The graph represents the Manders' colocalization coefficient calculated for LC3 and TrkB as well as vice versa TrkB with LC3 from confocal images of 13DIV hippocampal mouse neurons (wt vs. ko).

Apologies if I missed this somewhere but more information explaining the time course of the LTP experiment, the stimulation frequencies used to generate the LTP and a reminder of the rationale of the expt with the drug incubations would be helpful. As well as the differences in the last 10 minutes (presence of DCGIV) there are bigger differences in the period of time preceding this which is not mentioned.

Reply: We have extended the explanation regarding the LTP experiments in the corresponding figure legends (see also reply to reviewer 1). LTP was induced with a high frequency stimulation (HFS) protocol that consists of 4 trains of 100 pulses at 100Hz with an inter-train interval of 5 minutes. MF-LTP is a presynaptic, NMDA-independent form or LTP. Therefore, we used D-APV (NMDA receptor blocker) during baseline recordings and potentiation (blue-shaded box). The group II mGluR agonist DCG-IV (grey boxes) was used at the end of each experiment to control for input specificity, since the application of this drug suppresses synaptic transmission at the MF pathway.

A weakness of the biochemistry and localisation data is that there is a large quantity of descriptive

information which is displayed as n=1. I do not doubt that the authors will have done these experiments multiple times nor doubt the author's conclusions due to weight of data supporting their logic as well as the convincing peptide electrophysiology in the slices using the peptide of the binding site but, it would be helpful for the authors to quantify at least the major findings of the binding data in the early figures as done for the later figures as well as present the information as representative of n= x experiments.
Likewise, the localisation data is often one illustrative image (eg fig 2, 5, sup S5) and so some sort of quantification would make this more convincing. The line scans show the same n=1 as the images and so further or different quantification assessing colocalisation across multiple images would be more convincing.

Reply: We understand the argument of the reviewer but due to space limitations it is difficult to show several representative examples from descriptive panels and in particular, for images and line profiles.

However, we have performed quantifications for the major findings following the suggestion from the reviewer. Accordingly, we present a quantification of Pearson's coefficient between Synaptophysin and SIPA1L2 for panel 2a and the graph is added to the main figure (see Figure 2b). We believe that a more in-depth analysis for the data presented in panel 2e-f (former panels 2d-e) is available in figure 7c-e-f. We have also performed an analysis from panel 7a (see figure S6b-c) and we also show quantification of the motility of SIPA1L2 in dendrites (now figure S5d). We believe that data represented in panel S6e-f is supported by the analysis performed in S6g.

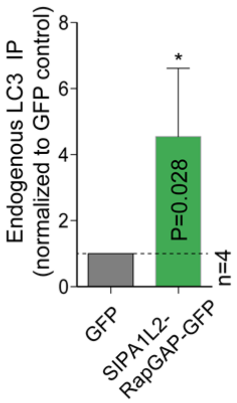

Regarding biochemical data, we have performed a quantification of the experiment depicted in Figure 4a that is shown here due to space limitations (left). This quantification adds to those already present in panel 4h-j-m (n numbers in figures correspond to number of independent experiments) and additionally supports our data. Please note that the panel in Figure 4a has been exchanged by a representative blot from the four novel experiments requested by the reviewer. Moreover, biochemical data are supported by imaging data with quantification. For instance, the association of SIPA1L2 with Snapin (Figure 3a), as well as TrkB binding mutant of SIPA1L2 in co-imunoprecipitation assay (Figure S4g) is supported with the co-recruitment study with line profiles (Figure 3b-c and Figure S4h accordingly).

We hope that we thereby could address the criticism of the reviewer.

The idea of showing the link between amphisome trafficking regulation and synaptic changes is really nice however, the quantification in terms of n# in figure 8 f and g is 10 fold different between sipa1l2+/+ + cherry and sipa1L2-/- + SIPA1L2Δ14-cherry. To make these conclusions more rigorous, this should be quantified by averaging the boutons per axon segment or coverslip ideally and averaging them, not just the averages of the boutons.

Reply: We agree with the reviewer. We have followed his/her suggestions and averaged the boutons per axonal segment and plotted the average from total axons analyzed per group. The panel and figure legend have been updated accordingly.

This applies also to the FM4-64 experiment detailed in fig 8 h-m. Further, this experiment compares the unloading of FM dye before and after a period of time allowing amphisome trafficking, however, unloading 1 and unloading 2 are not directly comparable since different vesicle pools are likely being monitored which could have different release rates. A better experiment would have been to reload with dye following the amphisome trafficking phase and then repeat the unload thus ensuring the same vesicle pools would be labelled. The rate of unloading is also a proxy of exocytosis and so a

more direct method looking at exocytosis specifically could have allowed specific conclusions about vesicle release to be made. The details of the n#s in terms of independent experiments have also been overlooked in the legend.

Reply: We included the missing information into the figure legend. Our experiment with FM4-64 was aimed to assess the effect of the amphisomal visits in presynaptic function. Therefore, we took advantage of FM4-64 technology since fluorescence decay is proportional to release probability (Newton et al., 2006) .

To allow for the recovery of synaptic terminals, we introduced a 10-minute interval between stimuli (unloading 1 and 2). Importantly, we did not observe any changes in unloading rates under control conditions i.e., in the absence of visits, the unloading rates recorded upon the second unloading stimulus remained unchanged when compared to those obtained in the same boutons upon the first stimulus. These control boutons belonged to axons were visits were reported. Therefore, we are confident that the differences we obtained between the unloading 1 and 2 are not due to the used protocol. Finally, it would be indeed interesting to further investigate whether the amphisomal visit and the presynaptic ERK activation directly alter the kinetics of exocytosis. However, we believe that this is out of the scope of this manuscript.

Minor points:
Since many of these figures are reliant on overexpression of tagged proteins it would be good to see that the levels of overexpression of wt is overexpressed to the same extent as the relevant mutants eg. Fig 5 Sipa1L2 wt and RapGAP dead mutant.

Reply: We believe that the expression levels are comparable between WT-SIPA1L2 and RapGAP mutant since we have used a similar amount of cDNA for transfection, a similar time window for protein expression, as well as similar settings /similar dynamic range for imaging. Unfortunately, there is no other way to detect the comparable expression of a fluorescently labeled protein in the single neuron within the culture rather than described above. Nevertheless, we provide below two snapshots from an axonal stretch from SIPA1L2-GFP and from SIPA1L2-N705A-GFP (RapGAP dead mutant) imaged during our experiments. Brightness and contrast are the same in both images. Scale bar is 2 µm.

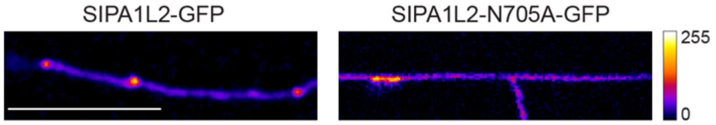

The characterisation of synaptic plasticity of the sipa1L2 KO presented in supplemental fig3 although technically not significantly different as per statistical tests, do not look overlaid either. Describing these as “normal” in the manuscript should be more cautiously described since although not significantly different, they do not look like “normal” either. It is not clear what the n# tested in this are? Are these individual animals, slices or sweeps and therefore is the experiment powered enough to detect significant differences?

Reply: Please see above the reply to reviewer 1: We believe that the observed differences are a result of interindividual variability and that these might appear larger than they really are due to the scale used in the graphs. More importantly, differences are not consistent among charts (see for instance Figure S3c-d). Therefore, we do not think that these statistically non-significant differences are of biological relevance. The n numbers stated in the figure correspond to slices.

The RapGAP activity assay uses a binding assay as a proxy for activity – a little more explanatory detail on the basis of the assay would make the experiment easier to interpret.

Reply: RapGAP activity for SIPA1L2 (including SIPA1L2 mutants) was assessed using active Rap1 detection kit (Cell Signaling) following the manufacturer instructions. The assay was firstly described in M'Rabet et al. (1998) where authors employed Rap1-binding domain (RBD) of RalGDS (RalGDS-RBD) as an activation-specific probe for Rap1 (see illustration in the panel a). Fused to GST-RalGDS-RBD allows to PD the amount of non-hydrolized Rap1 in the presence of RapGAP. Where space allowed us, for easier interpretation of the experiments (Figure 6g, Figure S5j) we have added explanatory cartoons into the timelines. In material and methods, we have now included more detailed information about the RapGAP activity assays. We have also added some additional explanations to the figure legend.

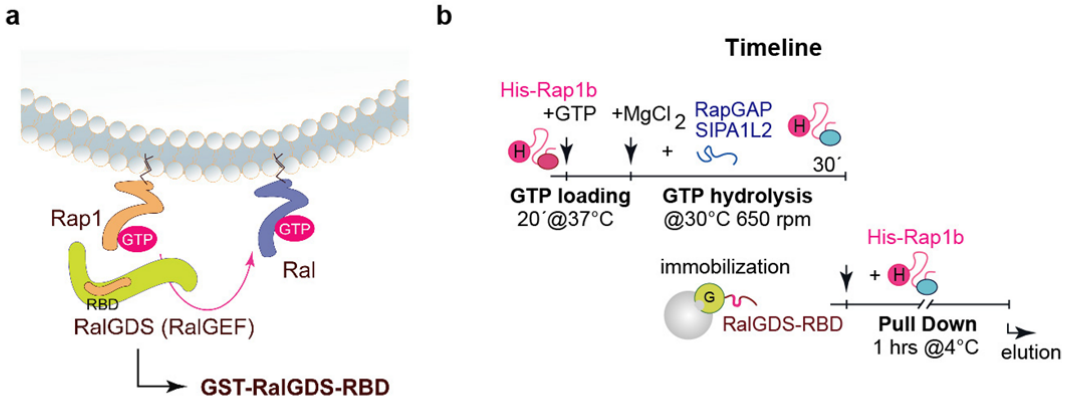

*Legend: (**a**) Cartoon represents the Rap1-binding domain of RalGDS (RalGDS-RBD) as an activation-specific probe for Rap1. (**b**) Example of an explanatory cartoon that we added to figure S5j where recombinant His-Rab1b was saturated with GTP in the loading buffer and subsequently loading was terminated by the addition of MgCl2. Untagged RapGAP domain of SIPA1L2 was added into the mixture for 30 minutes at 30°C on a shaker for GTP hydrolysis. Subsequently, GST-RapGDS-RBD was immobilized in Glutathione coated beads on a column. Protein mix was applied to the column where only non-hydrolized Rap1-GTP associated with RalGDS. By the amount of pulled-down Rap1b, we estimate the activity of RapGAP domain of SIPA1L2.*

The wt GFP-Snapin included in Fig 6 along with the phosphomimetics would have been a useful control to compare the difference with the phosphomimetics.

Reply: We think that the best control for the phosphomimetic version is the phosphordeficient Snapin mutant. In this set of experiments, we excluded the WT-Snapin for the reason that it is difficult if not impossible to control its phosphorylation state. We are therefore convinced that the addition of WT-Snapin might even obscure an effect of phosphorylation and would make it difficult to interpret the data.

Additionally, the stationary pool not compared to the wt or phosphonull.

Reply: The stationary pool of SIPA1L2 and wt GFP-Snapin is represented in the figure 3k.

The model proposed by the authors and illustrated in supplemental figure S7 is really nice – does it have to be in the supplemental? I think the discussion would benefit from this figure being in the main text.

Reply: We thank the reviewer for the positive comment and we included the cartoon in a separate main Figure 9.

References

BARANES, D., LOPEZ-GARCIA, J. C., CHEN, M., BAILEY, C. H. & KANDEL, E. R. 1996. Reconstitution of the hippocampal mossy fiber and associational-commissural pathways in a novel dissociated cell culture system. *Proc Natl Acad Sci U S A,* 93**,** 4706-11.

BERG, T. O., FENGSRUD, M., STROMHAUG, P. E., BERG, T. & SEGLEN, P. O. 1998. Isolation and characterization of rat liver amphisomes. Evidence for fusion of autophagosomes with both early and late endosomes. *J Biol Chem,* 273**,** 21883-92.

CAI, Q., LU, L., TIAN, J. H., ZHU, Y. B., QIAO, H. & SHENG, Z. H. 2010. Snapin-regulated late endosomal transport is critical for efficient autophagy-lysosomal function in neurons. *Neuron,* 68**,** 73-86.

CAZORLA, M., ARRANG, J. M. & PREMONT, J. 2011. Pharmacological characterization of six trkB antibodies reveals a novel class of functional agents for the study of the BDNF receptor. *Br J Pharmacol,* 162**,** 947-60.

LEE, K. J., QUEENAN, B. N., ROZEBOOM, A. M., BELLMORE, R., LIM, S. T., VICINI, S. & PAK, D. T. 2013. Mossy fiber-CA3 synapses mediate homeostatic plasticity in mature hippocampal neurons. *Neuron,* 77**,** 99-114.

LOPEZ-GARCIA, J. C., ARANCIO, O., KANDEL, E. R. & BARANES, D. 1996. A presynaptic locus for long-term potentiation of elementary synaptic transmission at mossy fiber synapses in culture. *Proc Natl Acad Sci U S A,* 93**,** 4712-7.

M'RABET, L., COFFER, P., ZWARTKRUIS, F., FRANKE, B., SEGAL, A. W., KOENDERMAN, L. & BOS, J. L. 1998. Activation of the small GTPase rap1 in human neutrophils. *Blood,* 92**,** 2133-40.

MANCIAS, J. D., WANG, X., GYGI, S. P., HARPER, J. W. & KIMMELMAN, A. C. 2014. Quantitative proteomics identifies NCOA4 as the cargo receptor mediating ferritinophagy. *Nature,* 509**,** 105-9.

NEWTON, A. J., KIRCHHAUSEN, T. & MURTHY, V. N. 2006. Inhibition of dynamin completely blocks compensatory synaptic vesicle endocytosis. *Proc Natl Acad Sci U S A,* 103**,** 17955-60.

PATEL, K. K., MIYOSHI, H., BEATTY, W. L., HEAD, R. D., MALVIN, N. P., CADWELL, K., GUAN, J. L., SAITOH, T., AKIRA, S., SEGLEN, P. O., DINAUER, M. C., VIRGIN, H. W. & STAPPENBECK, T. S. 2013. Autophagy proteins control goblet cell function by potentiating reactive oxygen species production. *EMBO J,* 32**,** 3130-44.

SCHILDT, S., ENDRES, T., LESSMANN, V. & EDELMANN, E. 2013. Acute and chronic interference with BDNF/TrkB-signaling impair LTP selectively at mossy fiber synapses in the CA3 region of mouse hippocampus. *Neuropharmacology,* 71**,** 247-54.

SPILKER, C., ACUNA SANHUEZA, G. A., BOCKERS, T. M., KREUTZ, M. R. & GUNDELFINGER, E. D. 2008. SPAR2, a novel SPAR-related protein with GAP activity for Rap1 and Rap2. *J Neurochem,* 104**,** 187-201.

WILLIAMS, M. E., WILKE, S. A., DAGGETT, A., DAVIS, E., OTTO, S., RAVI, D., RIPLEY, B., BUSHONG, E. A., ELLISMAN, M. H., KLEIN, G. & GHOSH, A. 2011. Cadherin-9 regulates synapse-specific differentiation in the developing hippocampus. *Neuron,* 71**,** 640-55.

WU, C. S., LIN, J. T., CHIEN, C. L., CHANG, W. C., LAI, H. L., CHANG, C. P. & CHERN, Y. 2011. Type VI adenylyl cyclase regulates neurite extension by binding to Snapin and Snap25. *Mol Cell Biol,* 31**,** 4874-86.

ZHOU, B., CAI, Q., XIE, Y. & SHENG, Z. H. 2012. Snapin recruits dynein to BDNF-TrkB signaling endosomes for retrograde axonal transport and is essential for dendrite growth of cortical neurons. *Cell Rep,* 2**,** 42-51.

REVIEWERS' COMMENTS:

Reviewer #1 (Remarks to the Author):

The authors addressed all my comments regarding the experiments in detail. There is still a lot of information within this one manuscript, but to me this is now more easily accessible in the revised version. I look forward to seeing this beautiful work published.

Reviewer #2 (Remarks to the Author):

The authors have addressed the critics I presented in my report to the original submission. The EM data the authors present in the rebuttal letter is not convincing, as also stated by the authors. It is better to omit the EM data from the manuscript.

A minor comment on nomenclature. The recommended name is 'autolysosome', not 'autophagolysosome'.

Reviewer #3 (Remarks to the Author):

I would like to thank the authors for including the new data and for modifying some of the previously included data. The paper does read more logically although I do still have concerns about the volume of data included. The expansion of the explanatory detail does help explain the techniques and results. The referees have addressed my specific points regarding additional experiments and my queries surrounding the n numbers and analysis have been acted on.

REVIEWERS' COMMENTS:

Reviewer #1 (Remarks to the Author):

The authors addressed all my comments regarding the experiments in detail. There is still a lot of information within this one manuscript, but to me this is now more easily accessible in the revised version. I look forward to seeing this beautiful work published.
Reply: We want to thank the reviewer for the positive comment and for his/her suggestions to the manuscript during the revision process.

Reviewer #2 (Remarks to the Author):

The authors have addressed the critics I presented in my report to the original submission.
Reply: We are happy to have addressed the reviewer´s comments with regard to the original submission and we are thankful to his/her suggestions during the revision of the manuscript.

The EM data the authors present in the rebuttal letter is not convincing, as also stated by the authors. It is better to omit the EM data from the manuscript.
Reply: We agree with the reviewer that the EM data are too preliminary and accordingly we omitted them from the manuscript.

A minor comment on nomenclature. The recommended name is 'autolysosome', not 'autophagolysosome'.
Reply: We have exchanged the name to “autolysosome” following the reviewer´s advice.

Reviewer #3 (Remarks to the Author):

I would like to thank the authors for including the new data and for modifying some of the previously included data. The paper does read more logically although I do still have concerns about the volume of data included. The expansion of the explanatory detail does help explain the techniques and results. The referees have addressed my specific points regarding additional experiments and my queries surrounding the n numbers and analysis have been acted on.
Reply: We would like to thank the reviewer for his/her positive comments and for his/her contribution to improve our manuscript with his/her suggestion. We indeed believe that our efforts to streamline the manuscript and include more explanatory details have significantly improved the logical flow and comprehensibility of our study.
